# The Nature of the Chemical Bond in Linear Three-Body Systems: From I_3_^−^ to Mixed Chalcogen/Halogen and Trichalcogen Moieties

**DOI:** 10.1155/2007/17416

**Published:** 2007-03-28

**Authors:** M. Carla Aragoni, Massimiliano Arca, Francesco A. Devillanova, Alessandra Garau, Francesco Isaia, Vito Lippolis, Annalisa Mancini

**Affiliations:** Dipartimento di Chimica Inorganica ed Analitica, Universitá degli Studi di Cagliari, S.S. 554 Bivio per Sestu, Monserrato, Cagliari 09042, Italy

## Abstract

The 3 centre-4 electrons (3c-4e) and the donor/acceptor or charge-transfer models for the description of the chemical bond in linear three-body systems, such as I_3_^−^ and related electron-rich (22 shell electrons) systems, are comparatively discussed on the grounds of structural data from a search of the Cambridge Structural Database (CSD). Both models account for a total bond order of 1 in these systems, and while the former fits better symmetric systems, the latter describes better strongly asymmetric situations. The 3c-4e MO scheme shows that any linear system formed by three aligned closed-shell species (24 shell electrons overall) has reason to exist provided that two electrons are removed from it to afford a 22 shell electrons three-body system: all combinations of three closed-shell halides and/or chalcogenides are considered here. A survey of the literature shows that most of these three-body systems exist. With some exceptions, their structural features vary continuously from the symmetric situation showing two equal bonds to very asymmetric situations in which one bond approaches to the value corresponding to a single bond and the second one to the sum of the van der Waals radii of the involved atoms. This indicates that the potential energy surface of these three-body systems is fairly flat, and that the chemical surrounding of the chalcogen/halogen atoms can play an important role in freezing different structural situations; this is well documented for the I_3_^−^ anion. The existence of correlations between the two bond distances and more importantly the linearity observed for all these systems, independently on the degree of their asymmetry, support the state of hypervalency of the central atom.

## 1. INTRODUCTION

**Scheme 1:**
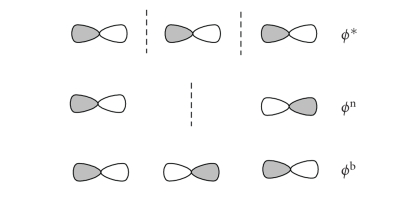
Rundle-Pimentel model for electron-rich 3c-4e systems.

**Scheme 2:**
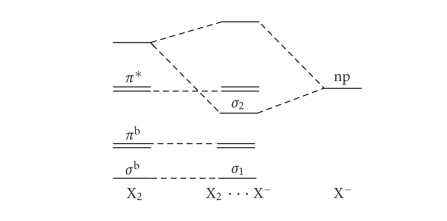
First approximation MO diagram for the donor/acceptor interaction between X^−^ and X_2_ fragments.

The chemical bond in linear three-body systems, such as trihalides, has been the object of many papers appeared very recently in the literature [1–5]. Among them, the paper on trihalides and hydrogen dihalides published by Hoffmann et al. [2], the book edited by Akiba [1] “Chemistry of hypervalent compounds” appeared in 1999, and the chapter on hypervalent chalcogen compounds by Nakanishi [4] in “Handbook of chalcogen chemistry” edited by F. A. Devillanova, represent authoritative contributions to this topic. In particular, the paper by Hoffman analyzes, on the basis of theoretical calculations, the various contributions to the stabilization of trihalides, by comparing the Rundle-Pimentel [6, 7] model for electron-rich 3-centre 4-electron systems (Scheme 1) with that describing the interhalogenic bond as a donor/acceptor or charge-transfer interaction between a halide and a dihalogen molecule (Scheme 2).

The commonly accepted 3 centre-4 electron bonding model considers the central halogen to be hypervalent. According to this model, a linear system formed for example by three aligned closed-shell I^−^ (24 shell electrons overall, I_3_^3−^) has no reason to exist since the three MOs generated by the combination of the three *p_z_*   orbitals, one from each interacting anion (Scheme 1), should be fully occupied by six electrons. However, the removal of two electrons from the antibonding MO causes an effective stabilization of the system and affords the well-known 22 shell electrons I_3_^−^ anion (Scheme 1). The stability of I_3_^−^ is determined by the occupancy of the lowest MO since the second filled MO is nonbonding in nature. The four electrons on the *σ* MOs plus the 6 electrons on the other three filled atomic orbitals equal a total of 10 electrons on the central I atom; this accounts for its description as an hypervalent species. However, a more formal counting indicates that only 8 electrons can be assigned to the central iodine since the HOMO is a combination out of phase of the *p_z_* orbitals of the external iodine atoms; consequently, no electron density coming from this MO can be assigned to the central iodine.

**Scheme 3:**
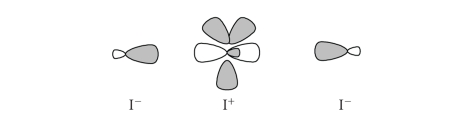
Donation of two lone pairs from two I^−^ into two empty hybrid orbitals around I^+^.

The alternative description of I_3_^−^ according to a Lewis model, which considers an *sp* ^2^ I^+^ cation having three lone pairs on the plane perpendicular to the bond direction and two hybrid orbitals able to linearly coordinate two I^−^ anions (Scheme 3), leads again to assign 10 electrons to the central iodine.

However, this model implies the combination of the *d^_z_^*2 and  atomic orbitals of the central I^+^ to afford two unfilled hybrid orbitals able to accommodate two lone pairs from the two I^−^ anions. According to this description, a simple notation, introduced by Arduengo et al. [5], identifies the central I atom as a 10−I−2 species, where “10” indicates the number of the electrons around the cental I, and “2” the number of the atoms or groups bonded to it. We must note that this formalism accounts very well for the hypervalent nature (expansion of the octet) of the central iodine, but does not account for the strength of the two bonds formed; in fact, according to such a model, each bond, arising from the localization of a pair of electrons from I^−^, should have a bond order of 1.

**Scheme 4:**
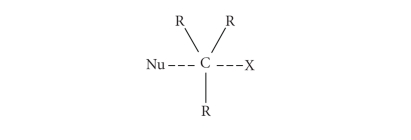
Transition state of an SN2_2_ type reaction at an *sp^3^* carbon atom (Nu = nuclephilic group, X = leaving group).

The simplest way to prepare a triiodide or in general a trihalide species considers the reaction between an X^−^ anion with an X_2_ molecule (X^−^ + X_2_→ X_3_^−^). In terms of chemical bond description, this corresponds to the commonly named donor/acceptor interaction (Scheme 2) since the bond is formed via a  *σ* donation from one of the four filled atomic orbitals of X^−^ (*np*) towards the empty  σ* antibonding molecular orbital of X_2_. As X^−^ formally approaches X_2_, the three lone pairs of the approached X atom of the X_2_ molecule are reoriented in order to be on the plane perpendicular to the bond direction when the symmetric three-body system X_3_^−^ is formed. It corresponds to the rearrangement of a *sp* ^3^ carbon atom during a nucleophilic attack in an SN_2_ type reaction (Scheme 4). The substantial difference between a trihalide and a penta-coordinated carbon resides in their different stabilities: while the penta-coordinated carbon represents a transition state, which finds its stabilization by removing one of the two apical groups so to allow the carbon to return to an *sp* ^3^ hybridization, X_3_^−^ is a stable species. In such systems, the counting of the electrons around the central halogen and carbon atoms agrees very well with the notation by Arduengo et al. [5] (10-X-2 and 10-C-5, resp.). In fact, since the starting diatomic species (X_2_ in the case of trihalides formation) obeys to the octet rule, every interaction with a donor (X^−^ in the case of trihalides formation) implies a transfer of electron density on X_2_, thus formally justifying a number of electrons higher than 8 on the central atom of the resulting three-body system.

**Scheme 5:**
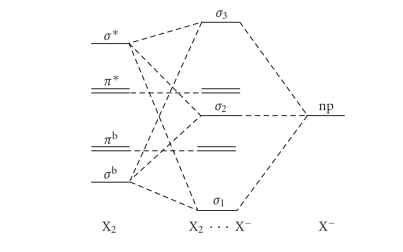
Second-order approximation MO diagram for the donor/acceptor interaction between X^−^ and X_2_ fragments (combination of np(X^−^) with both *σ* ^b^ and σ* of X_2_).

The simplified donor/acceptor first approximation MO diagram for the formation of a trihalide species (Scheme 2) becomes more complicate if the donor atomic orbital of X^−^ (*np*) is combined with both the σ* and the *σ* ^b^ MOs of X_2_. The result is a second approximation MO diagram having three new energy levels for the adduct, coming from the combination of these three orbitals (Scheme 5). ^1^

The difference between the first and second approximation MO diagrams (Schemes 2 and 5) resides in the nature of the first two MOs of the formed three-body system. In fact, the energy mixing in the second approximation diagram (Scheme 5) has the consequence of increasing the bonding nature of the lowest MO, moving the intermediate MO to higher energies towards a nonbonding nature. Now we can compare the 3c-4e bond model (Scheme 1) with the two MO diagrams for the donor/acceptor interaction between X^−^ and X_2_ (Schemes 2 and 5) to describe the chemical bond in X_3_^−^ anions. Since in all the three schemes, the highest MO is always an antibonding molecular orbital featuring a nodal plane between each couple of atoms, the differences between these models are mainly determined by the different nature of the lowest two molecular orbitals. According to the 3c-4e model, the stabilisation of electron-rich (22 shell electrons) three-body systems has to be ascribed only to the filling of the lowest MO, with a consequent bond order of 0.5 for each of the two bonds formed. According to the charge-transfer model, involving only the combination of a lone pair of X^−^ with the σ* MO of X_2_, the filling up of the lowest energy level corresponds to a bond order of 1 within the X_2_ fragment, while the filling up of the intermediate level accounts for the bond formation between the two interacting fragments and for a lengthening of the X−X bond in X_2_: a bond order of 0.5 for both bonds is reached in the symmetric situation X−X−X^−^. In the second approximation charge-transfer MO diagram (Scheme 5), the lone pair of X^−^ combines with both *σ* ^b^ and the σ* MOs of X_2_, consequently the bonding nature of first molecular orbital of the resulting three-body system is increased, and that of the second energy level is decreased, thus making this MO diagram intermediate between the 3c-4e and the first approximation charge-transfer MO diagrams (Schemes 1, 2, and 5).

## 2. DISCUSSION

The two bonding models (3c-4e and charge-transfer models) can be successfully employed to describe the chemical bond in numerous linear three-body systems featuring 22 shell electrons, formed by three aligned main group elements. The 3c-4e model describes linear three-body systems (electron-rich linear systems) in terms of interacting aligned closed-shell fragments; the stabilization is reached by removing a couple of electrons in order to leave unfilled the highest MO (Scheme 1). Scheme 1 shows the combination of three *p* orbitals lying at the same level of energy; in a more general scheme with different starting closed-shell fragments, the combined *p* orbitals lie at different energy levels with the consequence that they will contribute differently to each molecular orbital in the resulting three-body system. In particular, if the combined *p* orbital of one of the two external atoms lies at an energy level quite different from that of the *p* orbitals of the other two atoms, its contribution to the bonding MO will be poor with a consequent unbalancing of the two bonds. This case is normally better described with the charge-transfer model, which corresponds to the interaction between a donor and a 2c-2e bond system.

Another aspect that we must consider is the total charge brought by the final three-body system; it will depend only on the charges of the starting aligned closed-shell species. A very simple example is represented by the formation of the XeF_2_ molecule according to a 3c-4e model: the three-closed shell species to be considered are 2 F^−^ and Xe; by removing a couple of electrons the neutral XeF_2_ molecule is generated. When three equal or different X^−^ (X^−^ = halide) are aligned, the resulting three-body system will be a trihalide monoanion.

**Scheme 6:**
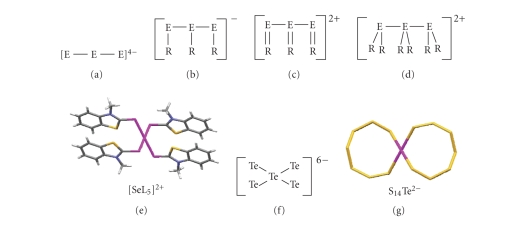
Different three-body systems featuring aligned chalcogen atoms. In (e) L=N-methylbenzothiazole-2(3H)-selone.

The situation is much more complex for the formation of three-body systems from closed-shell species of 16th group elements, since the closed-shell species which can be combined can be both charged (E^2−^, R–E^−^) and neutral (R_2_E and R=E, R = organic framework and E = chalcogen atom).^2^ In general, the alignment of three identical chalcogen species can afford three-body systems featuring very different charges, (a)–(d) in Scheme 6.

In principle, any combination of E^2−^, R–E^−^, R_2_E, and R=E species is possible, thus strongly increasing the variety of obtainable three-body systems. In addition, the central chalcogen atom of the three-body systems (a)–(d) reported in Scheme 6 can be aligned in turn to one or two other couples of closed-shell chalcogen species to form, after removal of 1 or 2 couples of electrons, systems featuring two ((e)–(g) in Scheme 6) or three orthogonal 3c-4e fragments, respectively. In this way we can explain the great variety of structural archetypes which contain a hypervalent chalcogen atom. The number of possible combinations further increases if mixed S, Se, and Te systems are also taken into account (see below).

Analogously to asymmetric trihalides, many asymmetric trichalcogen and mixed dichalcogen/halogen and chalcogen/dihalogen systems can be successfully described using the same charge-transfer model as that used for asymmetric trihalides. According to this model three-body systems arise from the interaction between a donor species (halide or chalcogen) and an acceptor species (dihalogen, dichalcogen, or chalcogen-halogen).^3^ Therefore, a trichalcogen arrangement derives from the n(E)→ σ*(E–E) interaction between one of the above-mentioned closed-shell chalcogen species acting as a donor and the empty σ* MO of a dichalcogen molecule acting as an acceptor. In the case of mixed halogen/chalcogen systems, depending on the starting species, different topologies of three-body systems can be obtained, such as E−X−Y, X−E−Y, E−E−X, and E−X−E (E = chalcogen, X,Y = halogen), which correspond to the well-known charge-transfer adducts between chalcogen donors and dihalogens (E−X−Y), “T-shaped” adducts of chalcogen donors (X−E−Y), dichalcogen molecules interacting with halides (E−E−X), and halogen(+) linearly coordinated by two chalcogen donor molecules (E−X−E).

## 3. TRIHALIDES

The Cambridge Structural Database (CSD) has been searched for discrete tr ihalides fragments contained in deposited crystal structures; the results of the search are collected in Table 1.^4^

**Figure 1:**
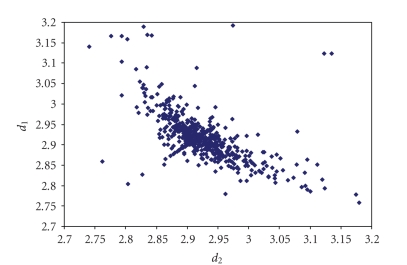
Scatter plot of  *d*_1_ versus *d*_2_  for linear (angle > 165°) triiodides from a search of the CSD (608 structures containing 815 fragments). The mean bond lengthening is 9.7% with respect to the sum of the covalent radii.

The triiodides are the most numerous and the scatter plot of the corresponding two I–I bond lengths is shown in Figure 1.

**Figure 2:**
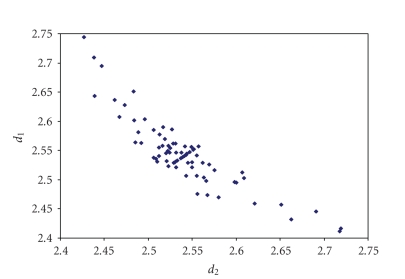
Scatter plot of *d*_1_ versus *d*_2_ for linear (angle > 165°) tribromides from a search of the CSD (71 structures containing 86 fragments). The mean bond lengthening is 11.3% with respect to the sum of the covalent radii.

**Figure 3:**
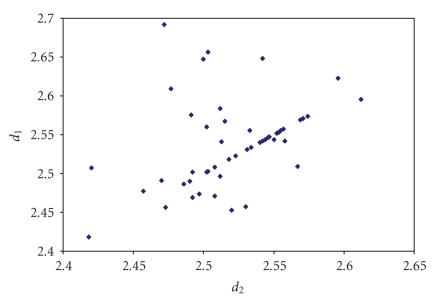
Scatter plot of *d*_1_ versus *d*_2_ for linear (angle > 165°) iododichlorides from a search of the CSD (46 structures containing 55 fragments). The mean bond lengthening is 9.2% with respect to the sum of the covalent radii.

**Figure 4:**
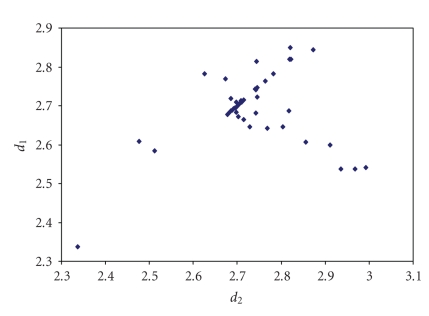
Scatter plot of *d*_1_ versus *d*_2_ for linear (angle > 165°) iododibromides from a search of the CSD (40 structures containing 56 fragments). The mean bond lengthening is 9.7% with respect to the sum of the covalent radii.

The literature related to triiodides has been omitted here and we refer to the paper by Svensson and Kloo [3]. Although several data are spread apart in the scatter plot,^5^ the majority of them are concentrated in the region corresponding to symmetric or weakly asymmetric triiodides. It is important to point out that an analogous correlation is found for Br_3_^−^anions (see Figure 2) [44–109] while for other trihalides, such as ICl_2_^−^ (Figure 3) [115–152] and IBr_2_^−^(Figure 4) [12–43], the corresponding scatter plots show a much less evident correlation. The structurally characterized Cl_3_^−^ fragments [154–160] are less than Br_3_^−^ and I_3_^−^ ones, and no bond length correlation diagram is presented for them. Except for the case reported by Gorge et al. [159] in which the two terminal chlorine atoms have significant contacts with two nitrogen atoms, in the other six reported structures containing the Cl_3_^−^ anion the two bonds are differently elongated, being 2.144/2.419 Å the bond distances found in the more asymmetric case (see Table 2). The number of structurally characterized mixed trihalides featuring two different terminal halogens (I–I–Br^−^ [8–11], I–I–Cl^−^ [110, 111], Cl–I–Br^−^ [112–114]) is very small, and a unique example of Cl–Br–Cl^−^ is reported in the literature (Tables 1 and 2) [153]. Among the considered fragments, we wish to emphasize the structural changes occurring on changing one of the terminal halogen from Cl, to Br and to I. Consider, for example, the I–Cl bond length in different trihalides: *d* (I–Cl) increases on passing from the symmetric Cl–I–Cl^−^ (mean value 2.53 Å) to Cl–I–Br^−^ (mean value 2.663 Å, Table 2) and Cl–I–I^−^ (mean value 2.889 Å, Table 2) indicating an increase in the ionic character of this bond when the other terminal halogen changes from Cl to Br and to I. However, in all cases, the I–Cl bond lengths are strongly elongated with respect to the sum of the covalent radii (2.39 Å) [161] but remain fairly shorter than the sum of the van der Waals radii (3.73 Å) [161]. The structural features of I–I–Cl^−^ and I–I–Br^−^ (Table 2) indicate that the bond distance of the central atom with the lighter halogen is always longer than the I–I distance, in accordance with a different ionic character of the two bonds. In terms of the 3c-4e model (Scheme 1), the *p*  orbitals of the two terminal halogens do not contribute equally to the three molecular orbitals of the three-body systems I–I–X^−^ (X = Cl, Br, I). In fact, the *p* orbital of the terminal atom featuring the better energy match with the *p* orbital of the central halogen will contribute more to the bonding MO; vice versa, the other halogen will mainly contribute to the nonbonding MO, thus carrying most of the negative charge. As a consequence, the bond orders of the two bonds diverge from the value of 0.5, one increasing towards the value of 1 (I–I), and the other decreasing towards the value of 0 (I–X). In terms of the charge-transfer model (Scheme 2), asymmetric trihalides of the type X–Z ⋯ Y^−^ derive from the donor/acceptor interaction between the halide (Y^−^) and the acceptor species (X–Z); the strength of this interaction will depend on the reciprocal energy levels of the combining orbitals (*p* of the halide and  σ* MO of the dihalogen molecule).

However, in all trihalides, independently of the different polarization of the two bonds the sum of the bond lengths (*d*_X−Z_ + *d*_Z−Y_) is always at least 9% longer than the sum of the covalent radii of the involved atoms, thus indicating a hypervalent state of the central halogen.

## 4. TRICHALCOGEN(IDE)S

Table 3 collects the occurrence of linear E–E′–E″ (E, E′, E″ = chalcogen atom) trichalcogen organic fragments found in structurally characterized compounds, as retrieved from a search of the Cambridge Structural Database (CSD) by imposing either the presence of two covalent bonds between the chalcogen atoms or the presence of one covalent bond and a nonbonding contact shorter than Σr_VdW_ – 0.3 Å (E ⋯ E′–E″ and E–E′ ⋯ E″ fragments). In both searches, the linearity of the fragment has been imposed (∠E–E′–E″ > 165^°^).

As one can see, some combinations of trichalcogen systems have never been reported and some others have been found only in a limited number of structures (Table 4).

**Figure 5:**
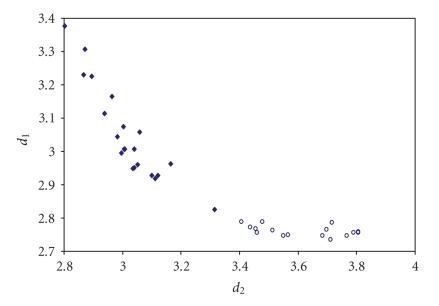
Scatter plot of *d*_1_ versus *d*_2_ for linear (angle > 165°) Te–Te–Te fragments from a search of the CSD. The symbol (♦) refers to the 23 Te–Te–Te fragments (19 structures) featuring bond distances ranging from Σr_cov_ to (Σr_VdW_–0.6); the symbol (°)  refers to the 16 Te ⋯ Te–Te fragments (12 structures) featuring Te ⋯ Te contact distances shorter than (Σr_VdW_–0.3). The mean bond lengthening within Te–Te–Te fragments is 11.5% (17.2% on Te ⋯ Te–Te fragments) with respect to the sum of the covalent radii.

**Figure 6:**
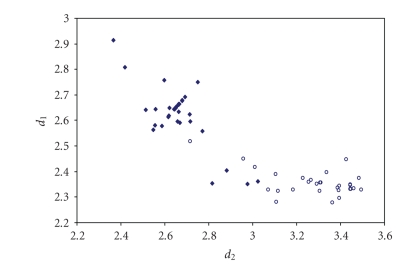
Scatter plot of *d*_1_ versus *d*_2_ for linear (angle > 165°) Se–Se–Se fragments from a search of the CSD. The symbol (♦) refers to the 35 Se–Se–Se fragments (22 structures) featuring bond distances ranging from Σr_cov_ to (Σr_VdW_ – 0.6); the symbol (°)  refers to the 29 Se ⋯ Se–Se fragments (21 structures) featuring Se Se contact distances shorter than (Σr_VdW_ – 0.3). The mean bond lengthening within Se–Se–Se fragments is 13.9% (21.5% on Se ⋯ Se–Se fragments) with respect to the sum of the covalent radii.

**Figure 7:**
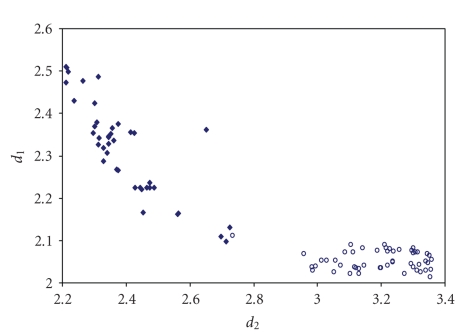
Scatter plot of *d*_1_ versus *d*_2_ for linear (angle > 165°) S–S–S fragments from a search of the CSD. The symbol (♦) refers to the 48 S–S–S fragments (40 structures) featuring bond distances ranging from Σr_cov_ to (Σr_VdW_ – 0.6); the symbol (°) refers to the 52 S ⋯ S–S fragments (24 structures) featuring S ⋯ S contact distances shorter than (Σr_VdW_ – 0.3). The mean bond lengthening within S–S–S fragments is 15.5% (28.6% on S ⋯ S–S fragments) with respect to the sum of the covalent radii.

**Figure 8:**
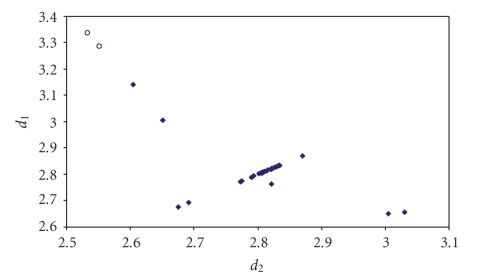
Scatter plot of *d*_1_ versus *d*_2_ for linear (angle > 165°) Se–Te–Se fragments from a search of the CSD. The symbol (♦) refers to the 39 Se–Te–Se fragments (22 structures) featuring bond distances ranging from Σr_cov_ to (Σr_VdW_ – 0.6); the symbol (°) refers to the 2 Se ⋯ Te–Se fragments (2 structures) featuring Se ⋯ Te contact distances shorter than (Σr_VdW_ – 0.3). The mean bond lengthening within Se–Te–Se fragments is 11.5% (16.1% on Se ⋯ Te–Se fragments) with respect to the sum of the covalent radii.

**Figure 9:**
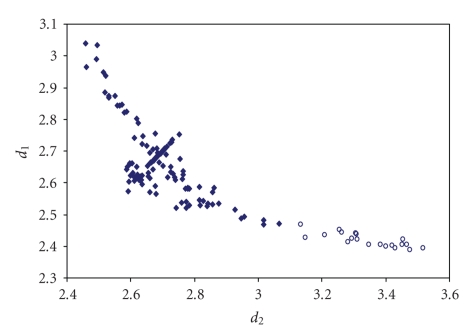
Scatter plot of *d*_1_ versus *d*_2_ for linear (angle > 165°) S–Te–S fragments from a search of the CSD. The symbol (♦) refers to the 187 S–Te–S fragments (127 structures) featuring bond distances ranging from Σr_cov_ to (Σr_VdW_ – 0.6); the symbol (°) refers to the 20 S ⋯ Te–S fragments (14 structures) featuring S ⋯ Te contact distances shorter than (Σr_VdW_ – 0.3). The mean bond lengthening within S–Te–S fragments is 12.6% (21.0% on S ⋯ Te–S fragments) with respect to the sum of the covalent radii.

The scatter plots of *d*(E–E′) versus *d*(E′–E″) for all trichalcogen fragments present in numerous crystal structures are shown in Figures 5–9.

Similar to what found for trihalides, linear trichalcogen systems can vary from symmetric to very asymmetric ones, but always feature strongly correlated *d*(E–E′) and *d*(E′–E″) bond lengths. This indicates that also in linear trichalcogen E–E′–E″ organic fragments the potential energy hole should be fairly flat, being the chemical surrounding of the chalcogen atoms and the crystal packing effects able to freeze different structural situations. As mentioned above, in the case of 16th group elements, different closed-shell chalcogen species can interact to afford different types of linear trichalcogen systems (Scheme 6). However, since the analysis of all linear trichalcogen systems would go beyond the aim of this work, we will focus our attention only on some of them. When the closed-shell species are three E^2−^ anions, the corresponding three-body systems will be E_3_^4−^. Indeed, the linear Te_3_^4−^, together with the “T-shaped” TeTe_3_^4−^, and the square-planar TeTe_4_^6−^ anions are considered fundamental building units of numerous polytellurides [359]. The Te–Te bond distances in such tellurides show elongation of about 13% with respect to the sum of the covalent radii and are typical for 3c-4e bonds [359]. A symmetric (Se_3_)^4−^ ion was identified for the first time in the samarium/selenide cluster [{(C_5_Me_5_)Sm}_6_Se_11_] [334], and considered a species isoelectronic to I_3_^−^. The Se–Se bond length in this system (2.749  Å) is much longer than the mean bond length in (Se_2_)^2−^ species (2.37  Å). This was justified by analogy with the couple I_2_/I_3_^−^. Linear [E–E ⋯ E]^4−^ systems (E= S,Se) have been found in Mo and W clusters [257, 335] containing two [M_3_(*μ*_3_-E)(*μ*-E_2_)_3_ (dtc)_3_]^+^ cores (M = Mo, E = S, Se; M = W, E = Se) linked via an E^2−^ anion; these arrangements have been described as *μ*−E_2_^2−^ dichalcogenides interacting with E^2−^ at significantly short distances. In the case of selenium clusters [335], the two Se–Se bonds are 2.355  Å  and 2.816  Å  for the Mo cluster and 2.38 Å  and 2.93 Å  (mean values) for the W one, the short distances being only slightly elongated with respect to the Se–Se bond length in diselenides (2.34 Å). As found for strongly asymmetric trihalides, which are better described as an X^−^ anion interacting with an X_2_ molecule (X^−^ ⋯ X_2_), the trichalcogen systems in which the two bonds assume very different bond orders should be better described as a chalcogen donor (in the present case E^2−^) interacting with the σ* antibonding molecular orbital of a dichalcogen species [n(E) → σ*(E–E), in the present case E_2_^2^^−^]. In other words, the interaction should occur between a chalcogen donor and a 2c-2e dichalcogen bond system. In general, depending on the starting chalcogen donor and dichalcogen acceptor species, these trichalcogen systems can carry a variable charge, from negative values as in the above cases, up to 2+ when the donor species is a neutral molecule and the acceptor a dichalcogen dication.

Several monoanionic structures of the type (R–E)_3_^−^, arising from three aligned R–E^−^ anions, for example, (Ph–Te)_3_^−^ [343, 356], or [(CN)Se–Se(Ph)–Se(CN)]^−^ [209], have been reported. Numerous are the hypervalent chalcogen compounds deriving from a neutral species interacting with two negatively charged monochalcogenides such as the case of 2,5-bis(morpholino-N)-4a-phenyl-1,3a,6,6a-tetrahydro-1,6,6a-triselena-4a*λ* ^4^-phospha-3,4-diazapentalene [332], or from a chalcogenide(2−) interacting with neutral molecules to form 1 or more 3c-4e systems, as in the case of the tetrakis(N-methylbenzo thiazole-2(3H)-selone)selenium(2+) dication reported by us in which two orthogonal 3c-4e fragments are present [342]. Particularly interesting are the two organic compounds FIKYON [251] and ZENJEH [252] (Table 4) containing the linear Se–Se–S arrangement. In fact, in both cases, the Se–Se bond is shorter than the Se–S one due to the poor energy match between the orbital of the central Se atom and that of the peripheral S atom. When the closed-shell species are S-, Se-, or Te-containing neutral molecules, dicationic species will be generated having the central atom in a hypervalent state. Among these systems, those having three S–S–S aligned sulphur atoms [265–314] have been found only in the class of the pincer-type molecules, with the central sulphur able to bind or to move apart the terminal ones by oxidation/reduction processes. It is noteworthy to observe that most of the molecules belonging to the E−E′−E pincer-type arrangments and many other trichalcogen systems are fairly symmetric, even if examples of strongly asymmetric situations are also numerous.

Although our discussion is limited to the structural features of linear trichalcogen fragments (various combinations of S, Se, and Te), we have also included in Table 4 the only known example of an organic dichalcogen dication system having a strong contact with an oxygen atom [357, 358]. X-ray analysis of this dication confirmed the linear geometry of the O–Se–Se moiety (165°) and an Se–Se bond (2.39 Å) which, similarly to what found in the above-described Mo and W clusters [257, 335], is only slightly elongated with respect to an Se–Se bond in diselenides (2.34 Å). This compound represents a good example of a hypervalent selenium compound having the two bonds strongly unbalanced (bond orders very far from the value of 0.5 expected for a balanced 3c-4e bond system). For this reason it resembles many other similar systems, such as the adduct of N,N′-dimethylimidazoline-2-selone with the *pseudo*-halogen ICN recently reported by us (see below in the last section); in both cases, one of the bonds tends to be a single bond, while the other bond is very elongated and tends to assume a purely ionic character.

## 5. DICHALCOGEN-HALIDES

Two chalcogen and one halogen atoms as closed-shell species can be aligned in only two possible ways: the halogen in the terminal (E−E′−X) or in the central (E−X−E′) position. Both arrangements are known and they will be discussed separately.

### 5.1. E−E′−X fragments

Table 5 shows the number of linear E−E′−X fragments crystallographically characterized from a search of the Cambridge Structural Database, by imposing the linearity of the system (∠E−E′−X angle > 165°) and either the presence of two covalent E−E′ and E′ – X bonds or the presence of one E−E′ covalent bond and one E′ ⋯ X nonbonding contact shorter than Σr_VdW_– 0.3 Å.

**Figure 10:**
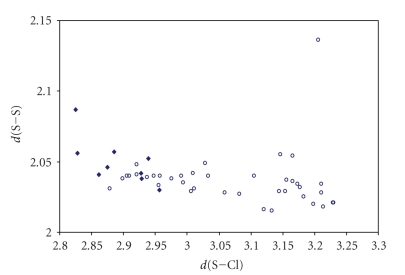
Scatter plot of *d*(S–S) versus *d*(S–Cl) for linear (angle > 165°) S–S–Cl fragments from a search of the CSD. The symbol (♦) refers to the 10 S–S–Cl fragments (4 structures) featuring bond distances ranging from Σr_cov_ to (Σr_VdW_ – 0.6); the symbol (°) refers to the 39 S–S ⋯ Cl fragments (17 structures) featuring S ⋯ Cl contact distances shorter than (Σr_VdW_ – 0.3). The mean bond lengthening within S–S–Cl fragments is 21.5% (26.1% on S–S ⋯ Cl fragments) with respect to the sum of the covalent radii.

**Figure 11:**
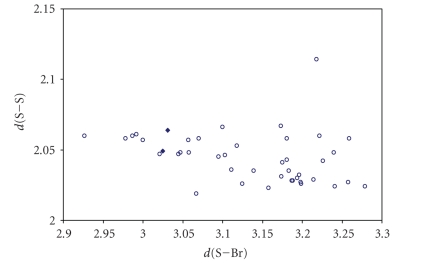
Scatter plot of (S–S) versus (S–Br) for linear (angle > 165°) S–S–Br fragments from a search of the CSD. The symbol  (°) refers to the 2 S–S–Br fragments (1 structure) featuring bond distances ranging from Σr_cov_ to (Σr_VdW_ – 0.6); the symbol  (°) refers to the 45 S–S ⋯ Br fragments (16 structures) featuring S ⋯ Br contact distances shorter than (Σr_VdW_ – 0.3). The mean bond lengthening within S–S–Br fragments is 21.1% (23.7% on S–S ⋯ Br fragments) with respect to the sum of the covalent radii.

**Figure 12:**
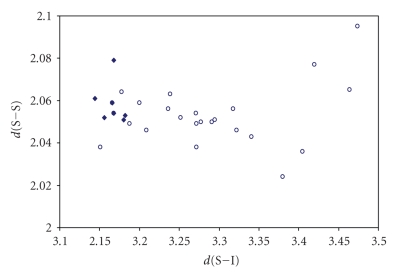
Scatter plot of *d*(S–S) versus *d*(S–I) for linear (angle > 165°) S–S–I fragments from a search of the CSD. The symbol (♦) refers to the 7 S–S–I fragments (4 structures) featuring bond distances ranging from Σr_cov_ to (Σr_VdW_–0.6); the symbol  (°) refers to the 25 S–S ⋯ I fragments (11 structures) featuring S ⋯ I contact distances shorter than (Σr_VdW_–0.3). The mean bond lengthening within S–S ⋯ I fragments is 19.2% (21.5% on S–S ⋯ I fragments) with respect to the sum of the covalent radii.

It is interesting to note that in the case of the S–S–X fragment (X = Cl, Br, I) the number of structures characterized by the presence of a linear S–S ⋯ X moiety is considerably higher than that featuring the S–S–X one (17 versus 4). Fragments having fairly covalent bonds have been found exclusively as part of some molybdenum clusters [390, 391, 399, 401, 445–447]. These clusters are very similar to those previously described in the discussion of trichalcogenides species, with the difference that the halide takes the place of the bridging E^2−^ anion. On the basis of their insolubility in water and their solubility in the common organic solvents, the authors concluded that the S–X bonds should be prevalently covalent in character. In fact, their structural features seem to be consistent with the presence of an [S–S–X]^3−^ anion, deriving from the removal of a couple of electrons from the aligned S^2−^, S^2−^, and X^−^ closed-shell species. The sum of the S–S and S–X bond lengths in these fragments is about 23% longer than the sum of the covalent radii and about 31% shorter than the sum of the van der Waals radii, in agreement with a 3c-4e bond model. The scatter plots of *d*(S–S) versu *d*(S–X) for both S–S–X and S–S ⋯ X (X = Cl, Br, I) fragments are shown in Figures 10, 11 and 12

It appears very clear from the figures that the points  (♦) related to the S–S–X fragments are not enough to establish the existence of a correlation between the two bond distances, and those (∘) related to the S–S ⋯ X fragments do not fit any correlation, since the S–S bond distances range in a very small interval of values around 2.05 Å (1.02 Å is the covalent radius of S), and the S ⋯ X contacts in a very wide interval of distances. These observations are more consistent with a description of these systems as deriving from donor/acceptor interactions between one of the *p* orbitals of X^−^ and the σ* antibonding MO on the S–S system. In these systems the energy-match between the lone pair of X^−^ and the σ* MO on S_2_^2−^ is very poor. The weak interaction is reflected in the corresponding very low lengthening of the S–S bond.

**Figure 13:**
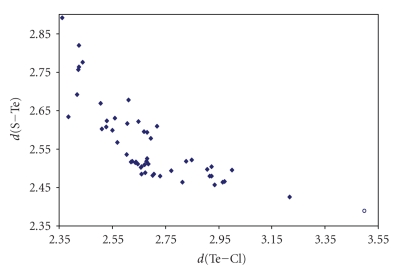
Scatter plot of *d*(S–Te) versus *d*(Te–Cl) for linear (angle > 165°) STeCl fragments from a search of the CSD. The symbol  (♦) refers to the 52 S–Te–Cl fragments (38 structures) featuring bond distances ranging from Σr_cov_ to (Σr_VdW_–0.6)(°)  refers to the 1 S–Te ⋯ Cl fragment (1 structure) featuring Te Cl contact distances shorter than (Σr_VdW_–0.3). The mean bond lengthening within S–Te–Cl fragments is 10.8% (24.5% on S–Te ⋯ Cl fragment) with respect to the sum of the covalent radii.

**Figure 14:**
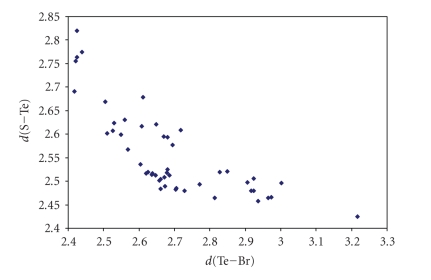
Scatter plot of *d*(S–Te)versus *d*(Te–Br) for the 52 linear (angle > 165°) S–Te–Br fragments (38 structures) featuring bond distances ranging from Σr_cov_ to (Σr_VdW_–0.6) from a search of the CSD. The mean bond lengthening within S–Te–Br fragments is 11.6% with respect to the sum of the covalent radii.

**Figure 15:**
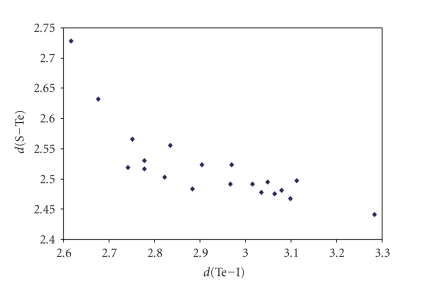
Scatter plot of *d*(S–Te) versus *d*(Te–I) for the 20 linear (angle > 165°) S–Te–Br fragments (19 structures) featuring bond distances ranging from Σr_cov_ to (Σr_VdW_–0.6) from a search of the CSD. The mean bond lengthening within STeBr fragments is 7.3% with respect to the sum of the covalent radii.

Numerous crystal structures have been reported in the literature that feature linear S–Te–X (X = Cl, Br, I) systems (references are collected in Table 5). Contrary to what found on searching the CSD for S–S–X fragments, for these linear arrangements, almost all the fragments feature covalent S–Te and Te–X bonds (S–Te–X systems). Only one structure containing an S–Te ⋯ Cl [244] moiety has been found by searching for S–Te ⋯ X systems [Σr_cov_ − 0.6 < *d*(T ⋯ X) < (Σr_VdW_ − 0.3)]. As shown in Figures 13, 14, and 15, for these three series of compounds the two bonds are strictly correlated in wide ranges of variability.

The sum of S–Te and Te–X bond distances within these three-body systems is 10.8%, 11.6%, and 7.3% longer than the sum of the covalent radii for X = Cl, Br, and I, respectively, in good agreement with the hypervalent nature of the central tellurium atom. The other mixed dichalcogen fragments bonded to a halide, characterized by X-ray diffraction, are very few and they are collected in Table 6.

Differently from S–S–X, the S–Se–X fragments have been found in some dimeric structures with bridging halides [383, 386]. Also in these cases the sum of the S–Se and Se–X bond lengths shows elongation (∼19%) with respect to the sum of the covalent radii, and shortening (∼30%) with respect to the sum of the van der Waals radii. A certain number of structures characterized by the linear Se–Te–X system have also been found. It is noteworthy that the S–Te and Se–Te bonds get shortened as the Te–X bond becomes more ionic (on changing X from I to Br and to Cl, see the examples reported in Table 7), their bond orders approaching the value of 1.

In the case of the chloroderivatives E–Te ⋯ Cl (E = S, Se, Table 7), the S–Te and Se–Te bonds are only 0.077 Å and 0.080 Å longer than the sum of the covalent radii, making the structural features of these compounds similar to those of the fragments Se–Se ⋯ O [358] and NC–Se ⋯ I (see below in the last section). Finally, five structures containing the linear Te–Te–I arrangement have been reported in the literature; two of them [437] are inserted in molybdenum clusters in a fashion similar to that found for the S–S–X and Se–Se–X groups, two are arranged to form (Ph–Te–I)_4_ tetramers [435, 436] and only one, (Mes)_2_Te–Te(Mes)–I [434], can be considered as derived from the three aligned closed-shell Mes_2_Te, MesTe^−^, and I^−^ species, by the removal of a couple of electrons. The analysis of the structural features of all these fragments is consistent with their description as three-body systems, the central Te atom being hypervalent.

### 5.2. E–X–E′ fragments

Table 8 collects the structural features of all the linear E–X–E′ (E, E′ = chalcogen atom; X = halogen) fragments found by searching the Cambridge Structural Database.

Systems of this type have been found with all the three halogens (Cl, Br, and I), and all the fragments have the same chalcogen (E = E′) atom at the two sides of the halogen; no mixed species (E≠E′) have been reported until now. Moreover, from the data in Table 8 it is interesting to note that with the exception of RIWDUW [473] which is polymeric and shows three different couples of fairly asymmetric Te–Cl bonds, all the other compounds feature the two chalcogen atoms bound to the central halogen in symmetric or only slightly asymmetric fashion, and most of the angles are very close to 180°. In all cases, the lengthening of the E–X bond with respect to the sum of the covalent radii (the mean S−I bond length calculated from the structural data of all six compounds characterized by the S−I−S group is elongated of about 17%), the shortening with respect to the van der Waals radii (the mean S−I bond length is shortened by ∼ 30%) and the linearity of the systems are consistent with the hypervalency of the central atom.

## 6. CHALCOGEN-DIHALIDES

Analogously to dichalcogen-halides, there are only two possibilities to build chalcogen-dihalides moieties: the chalcogen can be in the terminal (E–X–Y) or in the central (X–E–Y) position. These two arrangements correspond to the well-known CT and “T-shaped” adducts between chalcogen donors and dihalogens, respectively, and will be discussed separately.

### 6.1. E–X–Y fragments

For a more detailed discussion on this class of compounds the reader is referred to the review by Lippolis and Isaia [474]. The number of linear E–X–Y CT fragments crystallographically characterized from a search on the Cambridge Structural Database is reported in Table 9.

As one can see most of the adducts are obtained between sulphur donors (D) and diiodine, on the contrary, no compounds of this type are known with Te donors (the only reported structures featuring a Te–I–I arrangement are characterized by long I ⋯ I contacts). The n*σ*(D)→σ*(XY) charge-transfer model accounts very well for the chemical bond in these E–X–Y systems. Scheme 2 can be easily adapted to any type of donor/acceptor couple [with the substitutions of n *p* with n*σ*(D) and σ*(X_2_) with σ*(XY)], bearing in mind that each couple will have a proper match of energy between the interacting orbitals. We will focus our attention on the adducts between sulfur donors and I_2_, since for them it is possible to fine tune the lone pair energy of the donor atom by changing its chemical surrounding; therefore any type of adduct from very weak to extremely strong can be obtained. In the case of very weak interactions, each fragment holds its identity with a small reciprocal perturbation; the effect of such perturbation on the halogen molecule consists in the lowering to some extent of its bond order. In terms of the simplified MO diagram reported in Scheme 2, weak adducts correspond to a poor energy match between the interacting n*σ*(D) and σ*(I_2_) MO orbitals. Most of adducts between sulfur compounds and I_2_ belong to the class of weak adducts. Since the stabilization of the adduct only depends on the in-phase combination of the interacting orbitals, which is bonding between the donor atom and the central iodine, and antibonding between the two iodine atoms, the two bond lengths are strictly correlated and a shortening in the D ⋯ I bond distance is accompanied by a lengthening in the I–I one. Without doubt, such types of adducts must be considered two-coordinate hypervalent compounds of iodine, like I_3_^−^. However, there is a substantial difference between an I_3_^−^ and a D–I–I system; while in the case of I_3_^−^ the introduction of an asymmetry, by increasing removal of one terminal iodine as I^−^, generates in the limit case a strongly asymmetric I^−^ ⋯ I_2_ system, in the case of the charge-transfer adducts, two different asymmetric systems can be generated depending on which bond, D ⋯ I or I ⋯ I, is the weakest one. They correspond to two different charge-transfer adducts: n*σ*(D)→σ*(I_2_) and n*σ*(I^−^)→σ*(I–D). It is possible to pass almost continuously from a balanced situation with the two bonds having a bond order value of about 0.5 [10-I-2 “hypervalent system” for analogy to I_3_^−^], to the two different limit cases in which one bond assumes an increasingly ionic character. Consequently, also these limit cases featuring a strong asymmetry between the two bonds must be included among the 10-I-2 hypervalent compounds D–I^+^ ⋯ I^−^ and D ⋯ I–I.

**Figure 16:**
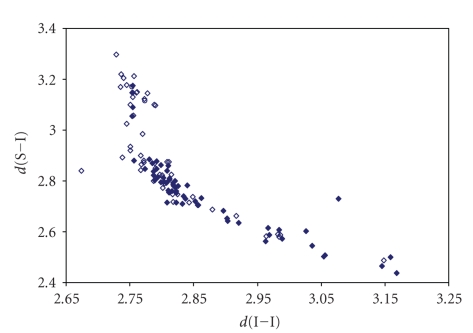
Scatter plot of *d*(S–I) versus *d*(I–I) for linear (angle > 165°) S–I–I fragments from a search of the CSD. The symbol (♦) refers to the 67 S–I–I fragments (50 structures) featuring bond distances ranging from Σr_cov_ to (Σr_VdW_–0.6); the symbol (◊) refers to the 50 S ⋯ I–I fragments (38 structures) featuring S ⋯ I contact distances shorter than (Σr_VdW_–0.3); the symbol  (°) refers to the 3 S–I ⋯ I fragments (3 structures) featuring I ⋯ I contact distances shorter than (Σr_VdW_–0.3). The mean bond lengthening within S–I–I fragments is 12.2% (19.2% on S ⋯ I–I, and 14.1% on S–I ⋯ I fragments, resp.) with respect to the sum of the covalent radii.

The scatter plot of *d*(S–I) versus *d*(I–I) relative to all the reported adducts between sulphur donors and diiodine is reported in Figure 16.

**Figure 17:**
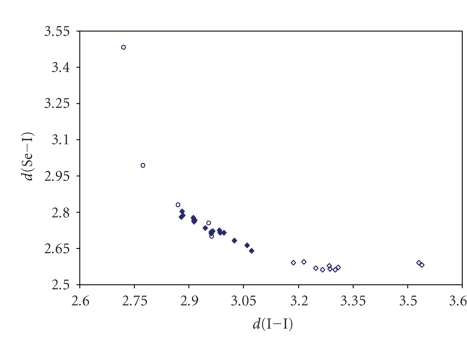
Scatter plot of *d*(Se–I) versus *d*(I–I) for linear (angle > 165°) Se–I–I fragments from a search of the CSD. The symbol (♦) refers to the 16 Se–I–I fragments (12 structures) featuring bond distances ranging from Σr_cov_ to (Σr_VdW_–0.6)(◊) refers to the 10 Se ⋯ I–I fragments (5 structures) featuring Se ⋯ I contact distances shorter than (Σr_VdW_–0.3). the symbol (°)  refers to the 6 Se–I ⋯ I fragments (6 structures) featuring I ⋯ I contact distances shorter than (Σr_VdW_–0.3). The mean bond lengthening within Se–I–I fragments is 10.5% (12.4% on Se ⋯ I–I, and 14.4% on Se–I ⋯ I fragments, resp.) with respect to the sum of the covalent radii.

**Figure 18:**
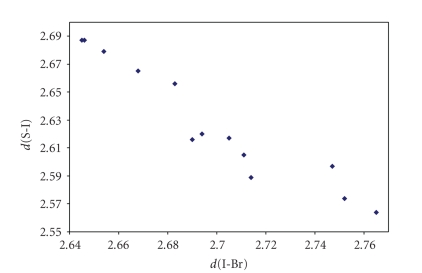
Scatter plot of *d*(I–Br) versus *d*(S–I) for the 13 linear (angle > 165°) S–I–Br fragments (11 structures) featuring bond distances ranging from Σr_cov_ to (Σr_VdW_–0.6) from a search of the CSD. The mean bond lengthening within S–I–Br fragments is 10.5% with respect to the sum of the covalent radii.

Apart for some dispersion of the data, which was also found for the other examined three-body systems, it clearly appears that the two bond lengths are strictly correlated in a wide range of values.^6^ Similar correlations have been found in the case of adducts of selenium donors with diiodine (Figure 17) and sulfur donors with IBr (Figure 18), well represented in the literature. Due to the paucity of experimental data, no correlation is evident in the analogous scatter plots for the linear adducts of chalcogen donors with the other dihalogen/interhalogens molecules, including the case of *d*(Te–I) versus *d*(I ⋯ I). The structural features of less common linear adducts between chalcogen donors and dihalogen/interhalogens molecules are collected in Table 10.

### 6.2. X–E–Y fragments

This arrangement corresponds to the well-known “T-shaped” adducts between chalcogen-donors and dihalogens. The numbers of linear X–E–Y fragments crystallographically characterized and found by searching the Cambridge Structural Database are reported in Table 11.

**Figure 19:**
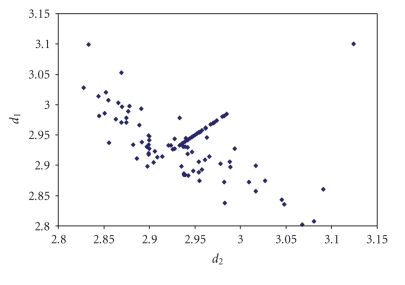
Scatter plot of  *d*_1_ versus *d*_2_  of the 113 linear (angle > 165°) I–Te–I fragments (71 structures) featuring bond distances ranging from Σr_cov_ to (Σr_VdW_–0.6) from a search of the CSD. The mean bond lengthening within I–Te–I fragments is 9.3% with respect to the sum of the covalent radii.

**Figure 20:**
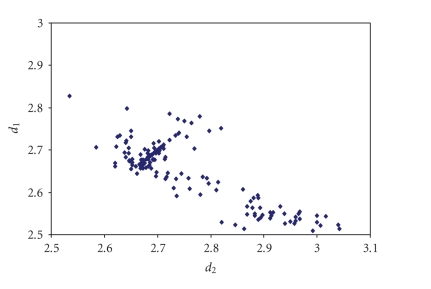
Scatter plot of  *d*_1_ versus *d*_2_  of the 170 linear (angle > 165°) Br–Te–Br fragments (84 structures) featuring bond distances ranging from Σr_cov_ to (Σr_VdW_–0.6) from a search of the CSD. The mean bond lengthening within Br–Te–Br fragments is 8.1% with respect to the sum of the covalent radii.

**Figure 21:**
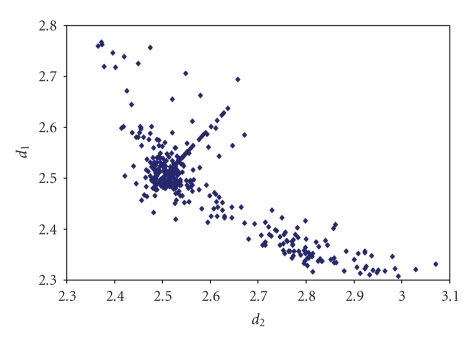
Scatter plot of *d*_1_ versus *d*_2_  for the 405 linear (angle > 165°) Cl–Te–Cl fragments (174 structures) featuring bond distances ranging from Σr_cov_ to (Σr_VdW_–0.6) from a search of the CSD. The mean bond lengthening within Cl–Te–Cl fragments is 8.0% with respect to the sum of the covalent radii.

**Figure 22:**
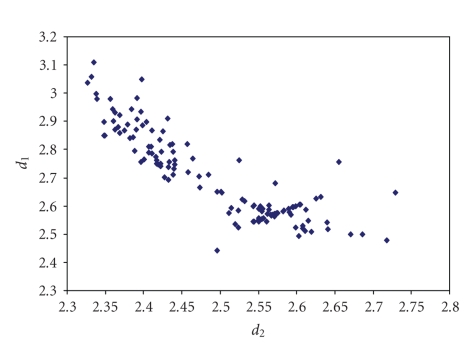
Scatter plot of  versus  for the 141 linear (angle > 165°) Br–Se–Br fragments (63 structures) featuring bond distances ranging from Σr_cov_ to (Σr_VdW_–0.6) from a search of the CSD. The mean bond lengthening within Br–Se–Br fragments is 12.9% with respect to the sum of the covalent radii.

**Figure 23:**
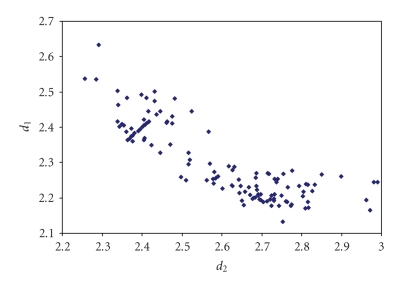
Scatter plot of  versus  for the 130 linear (angle > 165°) Cl–Se–Cl fragments (51 structures) featuring bond distances ranging from Σr_cov_ to (Σr_VdW_–0.6) from a search of the CSD. The mean bond lengthening within Cl–Se–Cl fragments is 13.8% with respect to the sum of the covalent radii.

While all the dihalogens/interhalogens combinations with selenium and tellurium have been reported in the literature, only few X–S–X moieties (X = Cl, Br)^7^, and no X–S–Y  (X ≠ Y = halogen atoms) arrangemets with sulphur as central atom are known. Indeed, sulphur donors show a preference to form linear charge-transfer type arrangement with the halogens (Table 9), absolutely unknown for the tellurium donors. Since several types of linear X–E–Y fragments are very numerous, the corresponding structural data are given as scatter plots of the two X–E and E–Y bond lengths (Figures 19, 20, 21, 22, and 23).

As one can see, there is a high dispersion of points in the scatter plots; however, in all the analyzed three-body systems the two bond lengths can be considered correlated and both strongly asymmetric and symmetric fragments can be found.

In Table 12, the structural features of less common X–E–Y fragments are reported; there are six examples of hypervalent chalcogen atoms bonded to two different halogen atoms, and, as already said, none of them features a central sulphur atom. It is interesting to note that in such systems the bond between the chalcogen and the lighter halogen is much more elongated with respect to the sum of the covalent radii (more ionic bond) than that involving the heavier halogen. In SUSMIC and in IDAZUI, Se–Cl and Se–Br are even longer than Se–Br and Se–I, respectively.

## 7. CHALCOGEN · XCN (X = HALOGEN) ADDUCTS

As reported before, we wish also to consider in this discussion “T-shaped” adducts obtained from the reaction between chalcogen donors and *pseudo*-halogens X–CN (X=Cl, Br and I). Some compounds characterized by X-ray diffraction analysis and featuring X–E–CN moieties (X = halogen, E = chalcogen) are collected in Table 13.

The compound CYMIMB, reported by Arduengo and Burgess [800], has been included in the table for its strict similarity with EZUZII, reported by us [832]. Both compounds have a “T-shaped” arrangement around the chalcogen atom and are characterized by very different E–X and E–CN bond lengths; the chalcogen-carbon bond is only sligthly elongated with respect to the sum of covalent radii (bond order close to 1) and the chalcogen-halogen bond is close to be a completely ionic bond. These compounds closely resemble many asymmetric systems above decribed and in particular the pincer-type molecule bearing the O ⋯ Se–Se group (Table 4). The closeness of the chalcogen–CN and the Se–Se bond distances to the corresponding single bonds, respectively, and the long chalcogen-halogen and selenium-oxygen distances, strongly support the analogy between these two classes of compounds. According to the 3c-4e model, the different energy levels of the three combined *p* orbitals (there is a good overlap between the orbitals from E and C due to a good match of their energies) produce a bonding MO having a small contribution of the  *p* orbital of the halogen, which vice versa mainly contributes to the nonbonding orbital, thus carrying most of the negative charge. In terms of the charge-transfer model, all the compounds of this type can be properly described as originated by a very weak donation from one halide orbital to the E–CN antibonding orbital (e.g., n*σ*(I^−^)→σ*(E–CN)); the weak interaction has the consequence of a small lengthening in the E–CN bond distance, exactly as verified in numerous adducts between weak S donors and diiodine.

## 8. CONCLUSION

On the basis of this overview on the structural features of linear three-body systems, involving 16th and 17th group elements, the following conclusions can be drawn.

- The Rundle-Pimentel model for electron-rich 3-centre 4-electron systems and the charge-transfer model represent two different approaches able to account for the structural features of these linear three-body systems.
- The Rundle-Pimentel model can be adapted to any set of three aligned atoms, positioning the combining orbitals at the appropriate levels of energy.
- Since three aligned closed-shell atoms can find stabilization only if two electrons are removed from the system, ideally any type of sequence of atoms could be obtained.
- The variability of starting molecules is reflected in the great variety of obtainable structural archetypes. Since a starting molecule can be also a species containing a hypervalent atom, its alignment with other closed-shell species produces molecules in which two or three orthogonal 3c-4e systems are simultaneous present. This is for example the case of anions such as Ph–SeBr_4_^−^ or SeBr_6_^2−^.
- The Rundle-Pimentel model very well accounts for the 0.5 bond order in symmetric three-body systems since only the lowest MO contributes to the bond formation. In addition, the Rundle-Pimentel model elegantly explains why the two terminal atoms carry more negative charge (or less positive charge for positively charged systems) even in the cases of three identical atoms [such as I_3_^−^ or E(R_2_) E(R_2_)E(R_2_)^2+^ dications].
- In these three-body systems, the energy match between the  *p* orbital of the central atom and those of the terminal ones influences the polarization of the formed bonds. This is very important for systems having different terminal atoms: each  *p* orbital will contribute differently to the three molecular orbitals with the consequence of an increased unbalance of the two bonds as the electronegativity difference between the involved elements increases. In such cases, the bond orders of the two bonds diverge from the value of 0.5, one approaching the value of 1 and the other that of 0.
- The strict analogy among all these systems, including the strongly asymmetric ones as the “T-shaped” adduct between the N,N′-dimethylimidazoline-2-selone and ICN, supports the hypervalent nature of the selenium atom in this compound in spite of the fact that the bond orders of the two bonds are very different.
- The charge-transfer model explains very well all the very asymmetric systems since this model corresponds to the interaction of two stable fragments (as a dihalogen molecule with a halide, or as chalcogen donor with a dichalcogen dication).
- The energy match between the interacting orbitals of the two fragments (such as a hybrid orbital of the donor and the σ* antibonding molecular orbital of the acceptor) determines the entity of the interaction.
- In the CT model, the bond order of 0.5 for the two bonds is reached when the interacting orbitals are at the same level of energy. This corresponds to the introduction of 1 electron on the σ* MO of the acceptor, with the consequent reduction of the bond order from 1 to 0.5.
- An aspect to be emphasized is the fact that in all the structures of these families of compounds, including the very asymmetric systems, the three-body system is always linear, with angles generally larger than 170° . The directionality of the bond is maintained also in presence of strongly unbalanced bonds indicating a valuable contribution of covalence, due to the n*σ*_donor_ → σ*_acceptor_ charge-transfer interaction and supporting the hypervalent character of the central chalcogen atom, independently on the entity of the asymmetry.
- Finally, it is interesting to observe that with only few exception, the systems having different terminal atoms (see, e.g., trihalides X–Z–Y with X≠Y or trichalcogenides E−E′−E″ with E≠ E″) are less common than the symmetric ones.

## Figures and Tables

**Table 1:** Occurrence of linear isolated trihalide X−Y−Z fragments crystallographically characterized from a search of the Cambridge Structural Database (number of crystal structures in parentheses).

		Y–Z =					

		I–I	I–Br	I−Cl	Br–Br	Br–Cl	Cl–Cl
	I	809 (608)^(a)^	∗	∗	—	—	—
X =	Br	5^(b)^	56 (40)^(c)^	∗	86 (71)^(d)^	—	—
	Cl	2^(e)^	4^(f)^	55 (46)^(g)^	—	1^(h)^	6^(i)^

^(a)^For the references of triiodides see [3]. ^(b)^References [8–11]. ^(c)^References [12–43]. ^(d)^References [15, 44– 109]. ^(e)^References [10, 110, 111]. ^(f)^References [112–114]. ^(g)^References [32, 35, 43, 110, 111, 114–152]. ^(h)^Reference [153]. ^(i)^References [154–160]. *These fragments are already considered in the table.

**Table 2:** Structural features of all the less common X–Z–Y linear trihalides characterized by X-ray diffraction analysis.

Compound reference code	X	Z	Y	*d*(X–Z)(Å)	*d*(Z–Y)(Å)	∠X–Z–Y(°)*	References
CUPTIQ	Cl	Cl	Cl	2.182	2.394	177.7	[154, 155]
DEGLIK	Cl	Cl	Cl	2.248	2.338	177.5	[156]
PHASCL	Cl	Cl	Cl	2.227	2.306	177.4	[157]
UHUQAP	Cl	Cl	Cl	2.144	2.419	178.1	[158]
ZEHTIP	Cl	Cl	Cl	2.262	2.307	178.4	[160]
TEACBR	Cl	Br	Cl	2.379	2.401	176.8	[153]
DOBTUJ	Cl	I	Br	2.648	2.651	179.6	[112]
DOBTUJ04	Cl	I	Br	2.670	2.675	179.4	[113]
DOBTUJ07	Cl	I	Br	2.673	2.665	179.6	[114]
DOBTUJ08	Cl	I	Br	2.670	2.662	179.8	[114]
BEQXEA	I	I	Cl	2.737	3.040	172.1	[110]
LACPUB	I	I	Cl	2.765	2.739	179.3	[111]
EKIHEL	I	I	Br	2.890	2.906	178.7	[8]
EYOVAP	I	I	Br	2.857	2.950	179.3	[9]
LACQAI	I	I	Br	2.775	2.856	178.7	[10]
LACQUEM	I	I	Br	2.780	2.857	176.6	[10]
WOPGOX	I	I	Br	2.786	2.794	179.2	[11]

*The angle values are rounded off to the first decimal digit.

**Table 3:** Occurrence of linear trichalcogen E−E′−E″, E ⋯ E′E″, and E−E′ ⋯ E″ fragments crystallographically characterized from a search of the Cambridge Structural Database (number of crystal structures in parentheses).

		E′–E″ =					

		Te–Te	Te–Se	Te–S	Se–Se	Se–S	S–S
	S	3 (2)^(a)^	4 (3)^(b)^	207 (141)^(c)^	12 (7)^(d)^	16 (9)^(e)^	100 (64)^(f)^
E =	Se	2 (2)^(g)^	41 (24)^(h)^	∗	64 (43)^(i)^	∗	—
	Te	39 (27)^(1)^	∗	∗	—	—	—

^(a)^References [162, 163]. ^(b)^Reference [164]. ^(c)^References [164–250]. ^(d)^References [251–256]. ^(e)^References [251, 256–264]. ^(f)^References [265–314]. ^(g)^References [315, 316]. ^(h)^References [162, 165, 174, 181, 183, 209, 216, 223, 228, 238, 317–323]. ^(i)^References [201, 324–342]. ^(1)^References [343–356]. *These fragments are already considered in the table.

**Table 4:** Structural features of less common E−E′−E″, E ⋯ E′−E″, or E−E′ ⋯ E″ trichalcogenides characterized by X-ray diffraction analysis.

Compound reference code	E	E′	E″	*d*(E–E′)(Å)	*d*(E′–E″) (Å)	∠E–E′–E″(°)^(b)^	References
BUWZUO	S	Se	S	2.266^(a)^	3.001^(a)^	172.2^(a)^	[259]
CEQKUE	S	Se	S	2.549	2.549	180.0	[260]
CUNWAJ	S	Se	S	2.534^(a)^	2.534^(a)^	180.0^(a)^	[261]
DUBKUG	S	Se	S	2.846^(a)^	2.295^(a)^	173.7^(a)^	[262]
FIKYUT	S	Se	S	2.467	2.371	170.0	[251]
KARZIM	S	Se	S	2.896^(a)^	2.282^(a)^	172.0^(a)^	[263]
SETIOP	S	Se	S	2.446	2.446	169.7	[264]
WAXMAJ	S	Se	S	3.302^(a)^	2.229^(a)^	169.2^(a)^	[256]
ZZZELOW01	S	Se	S	3.341	2.210	169.6	[257]
SOSNIX	S	Se	Se	3.002^(a)^	2.308^(a)^	167.3^(a)^	[253]
SOSNOD	S	Se	Se	2.977^(a)^	2.312^(a)^	167.3^(a)^	[253]
NPHSET	S	Se	Se	2.244	3.492	165.4	[254]
WADVOM	S	Se	Se	2.223	2.985	168.6	[255]
WAXMAJ	S	Se	Se	2.189	3.404	165.9	[256]
FIKYON	S	Se	Se	2.508	2.472	171.3	[251]
ZENJEH	S	Se	Se	2.498	2.466	173.6	[252]
FEZHIB	S	Te	Se	3.163	2.536	167.9	[162]
FEZHUN	S	Te	Se	2.592	2.872	175.3	[162]
FEZJEZ	S	Te	Se	3.002^(a)^	2.609^(a)^	173.4^(a)^	[164]
JOXYIE	S	Te	Te	3.508^(a)^	2.734^(a)^	170.4^(a)^	[162]
SISQUG	S	Te	Te	2.473	3.347	169.2	[163]
SEURBR	Se	Se	Se	2.712	2.624	173.9	[337]
SEURSL	Se	Se	Se	2.664	2.634	168.3	[339]
SECLUR	Se	Se	Se	2.717	2.597	173.8	[337]
BAWFUA	Se	Te	Te	2.561	3.611	176.1	[315]
YOMRIB	Se	Te	Te	2.468	3.559	173.3	[316]
ZONWOO	O	Se	Se	2.427^(d)^	2.391	165.0	[357, 358]

^(a)^Mean values. ^(b)^The angle values are rounded off to the first decimal digit. ^(c)^Triselenourea dications with different counterions. ^(d)^ *d*(O ⋯ Se).

**Table 5:** Occurrence of linear E−E′−X and E−E′ ⋯ X fragments crystallographically characterized from a search of the Cambridge Structural Database (number of crystal structures in parentheses).^(a)^

		E–E′ =					

		Te–Te	Se–Te	S–Te	Se–Se	S–Se	S–S
	Cl	6 (3)^(b)^	5 (5)^(c)^	53 (39)^(d)^	11 (8)^(e)^	7 (6)^(f)^	49 (21)^(g)^
X =	Br	—	17 (11)^(h)^	35 (26)^(i)^	23 (11)^(j)^	4 (2)^(k)^	47 (17)^(l)^
	I	19 (7)^(m)^	4 (4)^(n)^	20 (19)^(o)^	5 (3)^(p)^	3 (1)^(q)^	32 (19)^(r)^

^(a)^ Most of the structures have been found by imposing the presence of at least a contact between the E–E′ and X fragments (E–E′ ⋯ X) shorter than (Σr_VdW_ – 0.6). ^(b)^References [360–362]. ^(c)^References [317, 318, 363–365]. ^(d)^References [173, 174, 188–190, 194, 200, 204, 205, 212, 213, 235, 244, 317, 363, 366–381]. ^(e)^References [38, 360, 382–385]. ^(f)^References [260, 386–389]. ^(g)^References [390–410]. ^(h)^References [317, 363, 364, 370, 411, 412]. ^(i)^References [182, 196, 207, 212, 213, 232, 235, 317, 363, 365, 367, 372, 373, 377, 379, 413–418]. ^(j)^References [38, 398, 419–425]. ^(k)^Reference [386]. ^(l)^References [398, 405, 426–433]. ^(m)^References [434–438]. ^(n)^References [214, 364, 439, 440]. ^(o)^References [200, 213, 214, 235, 317, 372, 373, 378, 440–443]. ^(p)^References [38, 444]. ^(q)^Reference [383]. ^(r)^References [399, 445–458].

**Table 6:** Structural features of the less common E−E′−X and E−E′⋯X (E,E′ = S,Se, X = halogen) linear three-body systems and of some selected E−E′−X (E = S,Se, E′ = Te, X = halogen) fragments.

Compound reference code	E	E′	X	*d*(E–E′) (Å)	*d*(E′–X)(Å)	∠ E–E′–X(°)^§^	References
BOYXAO10	S	S	Cl	2.040*	2.915*	169.6*	[390]
FAVDUB	S	S	Cl	2.053*	2.863*	166.9*	[391]
KOJHOG	S	S	Cl	2.047*	2.933*	170.4*	[399]
PIGWIL	S	S	Cl	2.087	2.825	165.1	[401]
				2.179	2.573	168.3	
KOJHUM	S	S	Br	2.056*	3.028*	171.5*	[399]
CIKHUZ10	S	S	I	2.057*	3.168*	171.2*	[445]
JAKWAT	S	S	I	2.057*	3.150*	173.1*	[446]
KOJJEY	S	S	I	2.066*	3.175*	172.6*	[399]
PEHHOZ	S	S	I	2.051	3.180	172.8	[447]
QADHOS	S	Se	I	2.218*	3.149*	168.3*	[383]
MURXOM	S	Se	Br	2.285*	3.007*	175.0*	[386]
MURYAZ	S	Se	Br	2.258*	3.094*	174.3*	[386]
MURXIG	S	Se	Cl	2.273*	2.920*	174.8*	[386]
MURXUS	S	Se	Cl	2.252	2.976	172.8	[386]
CEQKOY	S	Se	Cl	2.215	3.276	178.5	[260]
KAXWEL	S	Se	Cl	2.293	3.237	168.8	[387]
NEDBAZ	S	Se	Cl	2.136	3.212	171.9	[389]
TAVXET	Se	Se	Cl	2.440	2.778	172.1	[38]
TAVXIX	Se	Se	Br	2.424	2.830	166.7	[38]
PEBPUH	Se	Se	Br	2.403	3.036	174.2	[419]
WOHDUS	Se	Se	Br	2.529*	2.689*	174,4*	[420]
EZOYIB	Se	Te	I	2.906	2.889	177.7	[439]
FOBCEE	Se	Te	I	2.618	3.251	173.5	[364]
ISEUTE	Se	Te	I	2.679	3.095	177.3	[440]
ROMXEW	Se	Te	I	2.721	2.967	177.5	[214]
BSEUTE	Se	Te	Br	2.616	3.054	175.6	[370]
DEVHAN	Se	Te	Br	2.769	2.761	175.2	[363]
FOBBIH	Se	Te	Br	2.678	2.898	173.9	[317]
FOBBIH01	Se	Te	Br	2.673*	2.907*	173.7*	[317]
FOBCAA	Se	Te	Br	2.572*	3.096*	172.9*	[364]
FOBCAA01	Se	Te	Br	2.582	3.086	174.0	[364]
FOBCAB	Se	Te	Br	2.648	2.854	174.8	[364]
KIKPID	Se	Te	Br	2.496*	3.244*	168.6*	[411]
NAHWIC	Se	Te	Br	2.704*	2.810*	175.0*	[412]
NAWOI	Se	Te	Br	2.763	2.744	177.0	[412]
FOWMAF	Se	Te	Br	2.540	3.289	174.3	[318]
DEVGUG	Se	Te	Cl	2.783	2.600	174.7	[363]
FOBBED	Se	Te	Cl	2.678	2.752	172.8	[317]
FOBBUT	Se	Te	Cl	2.664	2.701	175.6	[364]
GANHIM	Se	Te	Cl	2.592	2.972	171.6	[365]
BETDAG	Te	Te	I	3.283	2.814	166.7	[434]
HOJJEV	Te	Te	I	3.163	2.831	176.1	[435]
HOJJEV01	Te	Te	I	3.158	2.817	175.6	[436]
HOSCAT	Te	Te	I	2.669*	3.369*	169.0*	[437]
HOSCEX	Te	Te	I	2.644*	3.329*	167.8*	[437]

^§^The angle values are rounded off to the first decimal. *Mean values.

**Table 7:** Examples of the shortening of the S–Te and Se–Te bonds on passing from I to Br and to Cl derivatives.

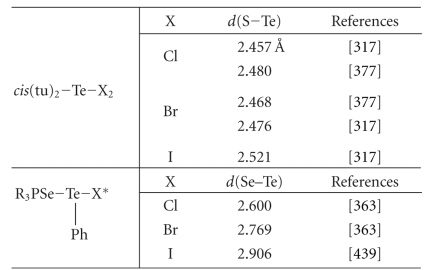

*R = morpholino for X = Cl and Br; R = butyl for X = I.

**Table 8:** Structural features of all the dichalcogen-halogen (E–X–E′) fragments determined by X-ray diffraction analysis (the E–X ⋯ E′ fragments have not been reported).

Compound reference code	E	X	E′	*d*(E−X) (Å)	*d*(X−E′) (Å)	∠E−X−E′(°)^§^	References
HAKJAE	S	I	S	2.601	2.634	175.0	[459]
IBOCUX	S	I	S	2.644	2.685	171.9	[460]
IOENCO	S	I	S	2.610	2.610	173.0	[461]
ISUREA10	S	I	S	2.629	2.629	180.0	[462]
LOPQAI	S	I	S	2.638*	2.618*	179.0*	[463]
XORVRAB	S	I	S	2.654	2.654	180.0	[464]
GIGBED	S	I	S	2.406	3.211	175.6	[465]
DIJYUQ	Se	I	Se	2.767	2.737	170.3	[466]
EZOXUM	Se	I	Se	2.765	2.765	180.0	[467]
HAKHUW	Se	I	Se	2.800	2.719	178.0	[459]
CEMFAB10	Te	I	Te	3.124	3.100	189.0	[468]
LAQZEI^$^	Se	Cl	Se	2.537	2.805	175.8	[469]
GANGIL**	Se	Br	Se	2.608	2.606	175.9	[470]
VIYRIE**	Se	Br	Se	2.615	2.573	176.1	[471]
MUHGUR	Se	Br	Se	3.089*	3.083*	178.8*	[472]
RIFNUP	Te	Cl	Te	2.755	2.755	180.0	[142]
ZUNJAT	Te	Cl	Te	2.857	2.829	171.4	[362]
RIWDUW^‡^	Te	Cl	Te	2.664*	2.988*	172.3*	[473]

^§^The angle values are rounded off to the first decimal. *Mean values. **The Se–Br–Se arrangement is part of the Br_14_Se_4_^2−^ anion. ^‡^Polymeric structure. ^§^The Se–Cl–Se arrangement is part of the SeCl_5_^−^ anion.

**Table 9:** Occurrence of linear E–X–Y CT fragments crystallographically characterized from a search of the Cambridge Structural Database (number of crystal structures in parentheses).

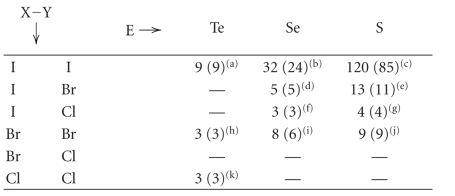

^(a)^Only contacts. References [476–483]. ^(b)^References [42, 444, 466, 484–498]. ^(c)^References [47, 69, 108, 463, 464, 474, 490, 493, 496, 499–549]. ^(d)^References [30, 42, 148]. ^(e)^References [25, 47, 69, 510, 550–554]. ^(f)^References [148, 555, 556]. ^(g)^References [510, 553–555]. ^(h)^Only contacts. References [480, 557, 558]. ^(i)^Only the structure of [559] is a CT adduct. References [50, 559–561]. ^(j)^Only two structures are of the CT type. References [45, 63, 475, 532, 562–564]. ^(k)^Only contacts. References [360, 483, 565].

**Table 10:** Structural features of less common E–X–Y linear chalcogendihalides of the CT type, characterized by X-ray diffraction analysis.

Compound reference code	E	X	Y	E–X (Å)	X–Y (Å)	∠ E–X–Y(°)^(a)^	References
HAMCII	S	I	Cl	2.534	2.761	176.4	[510]
LIFXIH	S	I	Cl	2.556	2.604	179.9	[555]
NAHQIX	S	I	Cl	2.575	2.558	176.1	[553]
SIBJOC	S	I	Cl	2.641	2.586	174.9	[554]
RORNIV^(b)^	S	Br	Br	2.299	2.717	175.0	[562]
RORNIV01^(b)^	S	Br	Br	2.328	2.705	176.0	[63]
IRABEI^(c)^	Se	Br	Br	2.645	2.358	174.2	[559]
LIGFIQ	Se	I	Cl	2.625	2.690	178.9	[555]
LIGFIQ01	Se	I	Cl	2.618	2.690	178.7	[148]
OXSEIC	Se	I	Cl	2.630	2.731	175.8	[556]
NOWLOA	Se	I	Br	2.808	2.641	177.3	[30]
NOWLUG	Se	I	Br	2.664	2.797	175.8	[30]
WIPPAM	Se	I	Br	2.636	2.813	177.1	[148]
YEYFIR	Se	I	Br	2.689	2.908	176.9	[42]

^(a)^The angle values are rounded off to the first decimal. ^(b)^Polymorphs. ^(c)^This is the unique example of CT type adduct between a selenium donor with bromine: the formation of a Br–Se–Br group determines very favorable electronic and steric effects to prevent the formation of the same arrangement on the second selenium atom and to promote the CT type adduct. It must be noted that the Se ⋯ Br interaction is enough weak to determine a lengthening of the Br–Br bond of only 0.078 Å.

**Table 11:** Occurrence of linear X–E–Y fragments crystallographically characterized from a search of the Cambridge Structural Database.

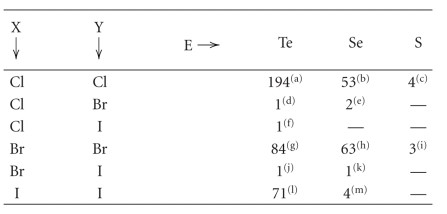

^(a)^References[142,169,173,180,181,184,188–190,203,206,217,219,233,240,360–362,368,373,374,376,381,473,480,481,565–699 ^(b)^References [260, 325, 381, 469, 700–729]. ^(c)^References [730–732]. ^(d)^Reference [733]. ^(e)^Reference [723]. ^(f)^Reference [580]. ^(g)^References [61, 183, 217, 220, 232, 233, 240, 318, 373, 411, 417, 480, 481, 558, 569, 574, 607, 615, 625, 627, 635, 651, 669, 675, 688, 691, 734–777]. ^(h)^References [50, 76, 96, 419, 421, 470, 471, 700, 495, 559– 561, 718, 723, 724, 727, 752, 778– 799]. ^(i)^References [47, 788, 800]. ^(j)^Reference [801]. ^(k)^Reference [802]. ^(l)^References [183] [207] [220, 233, 240, 317, 373, 435, 442, 468, 476–483, 593, 635, 672, 680, 753, 770, 801, 803–829]. ^(m)^References [486, 498, 830].

**Table 12:** Structural features of the less common X–E–Y linear chalcogendihalides characterized by X-ray diffraction analysis.

Compound reference code	X	E	Y	*d*(X−E) (Å)	*d*(E−Y) (Å)	∠ X–E–Y(°)^(a)^	References
CFMBXT	Cl	S	Cl	2.126	2.552	167.6	[730]
CLPHSC10	Cl	S	Cl	2.256	2.322	174.9	[731]
TOSXII	Cl	S	Cl	2.341	2.384	166.3	[732]
TOSXOO	Cl	S	Cl	2.295	2.365	175.9	[732]
BIMMAL	Br	S	Br	2.437	2.495	171.6	[47]
MIMZDB	Br	S	Br	2.451	2.538	176.9	[800]
OBUQEH	Br	S	Br	2.493	2.493	179.4	[788]
SUSMIC	Cl	Se	Br	2.802^(b)^	2.412^(b)^	173.3^(b)^	[723]
SUSNAV	Cl	Se	Br	2.466	2.571	176.2	[793]
IDAZUI^(c)^	Br	Se	I	2.831^(b)^	2.618^(b)^	174.6^(b)^	[802]
GEPPUM	I	Se	I	2.756	2.850	176.3	[486]
HELDUX	I	Se	I	2.768	2.854	175.4	[830]
ZOBDID	I	Se	I	2.738	2.886	178.6	[498]
ZOBDUP	I	Se	I	2.743	2.900	177.5	[498]
XAGVIK	Cl	Te	Br	2.659	2.577	169.9	[733]
CEFREX	Br	Te	I	2.868	2.903	177.9	[580]

^(a)^The angle values are rounded off to the first decimal. ^(b)^Mean values. ^(c)^This compound is the unique example of Se-hypervalent compound with IBr. Note that the mean value of the Se–I bond length is shorter than the Se–Br one.

**Table 13:** Structural features of all the T-shaped compounds containing the X ⋯ E–CN fragment (E = chalcogen; X = halogen) from a search of the Cambridge Structural Database.

Compound reference code	X	E	X ⋯ E (Å)	E–CN (Å)	∠ X ⋯ E–Y(°)	References
BOJPUL	Cl	Te	2.924	2.140	167.9	[831]
BOJRAT	Br	Te	3.100	2.131	167.6	[831]
BOJREX	I	Te	3.299	2.143	170.9	[831]
CYMIMB^(a)^	Br	S	3.588^(a)^	1.757	159.8	[800]
EZUZII	I	Se	3.300	1.885	174.8	[832]

^(a)^This compound has not been found searching the Cambridge Structural Database, but has been included in the table for the strict similarity with EZUZII. In CYMIMB, the shorter S ⋯ Br distance (3.270 Å) is that of the bromide in *trans* position with respect to the pentaatomic ring of the donor and not to the CN group.

## Footnotes

^1^However, in the construction of the simplified MO diagram for the donor/acceptor interaction (first approximation; Scheme 2), the combination of the donor orbital with only the *σ** MO of X_2_, leaving unchanged the *σ* ^b^ level, is justified fairly well by a good match of energy between the lone pair of X^−^ and the *σ** MO of X_2_.

^2^In the case of 17th group elements, not only X^−^but also R′ − X (R′ represents a generic fragment featuring whichever element bonded to the halogen such as C–X, P–X,…) closed-shell species could be considered in the formation of three-body systems; however, these cases are not considered here in order to limit the discussion.

^3^As an exception, we will also consider the *pseudo*-halogens X–CN (X= halogen atom, see below in the last section).

^4^Only covalent bonded linear X–Y–Z fragments (angles > 165°) have been considered in subsequent analyses.

^5^A discussion on the factors responsible for the spreading of some data out of the correlation in Figure 1 is out of the aim of this work.

^6^The points having the S–I distance higher than 3.0 Å refer to quite different systems having the diiodine molecule bridging two donor molecules.

## References

[1] Akiba K-Y (1999). *Chemistry of Hypervalent Compounds*.

[2] Landrum GA, Goldberg N, Hoffmann R (1997). Bonding in the trihalides (X_3_
^−^), mixed trihalides (X_2_Y^−^) and hydrogen bihalides (X_2_H^−^). The connection between hypervalent, electron-rich three-center, donor-acceptor and strong hydrogen bonding. *Journal of the Chemical Society, Dalton Transactions*.

[3] Svensson PH, Kloo L (2003). Synthesis, structure, and bonding in polyiodide and metal iodide-iodine systems. *Chemical Reviews*.

[4] Nakanishi W, Devillanova FA (2006). Hypervalent chalcogen compounds. *Handbook of Chalcogen Chemistry, New Perspectives in Sulfur, Selenium and Tellurium*.

[5] Perkins CW, Martin JC, Arduengo AJ, Law W, Alegrie A, Kocki JK (1980). An electrically neutral σ-sulfuranyl radical from the homolysis of a perester with neighboring sulfenyl sulfur: 9-S-3 species. *Journal of the American Chemical Society*.

[6] Hackand RJ, Rundle RE (1951). The structure of tetramethylammonium Pentaiodide^1, 1a^. *Journal of the American Chemical Society*.

[7] Pimentel GC (1951). The bonding of trihalide and bifluoride ions by the molecular orbital method. *The Journal of Chemical Physics*.

[8] Mas-Torrent M, Ribera E, Tkacheva V (2002). New molecular conductors based on ETEDT-TTF trihalides: from single crystals to conducting layers of nanocrystals. *Chemistry of Materials*.

[9] Kazheva ON, Aleksandrov GG, D'yachenko OA, Chernov'yants MS, Simonyan SS, Lykova EO (2003). Crystal and molecular structures of 3-carboxypropyltriphenylphosphonium diiodobromide. *Koordinatsionnaya Khimiya*.

[10] Shilov GV, Kazheva ON, D'yachenko OA (2002). The synthesis, structure, and stability of N-cetylpyridinium interhalides: an experimental and quantum-chemical study. *Zhurnal Fizicheskoj Khimii*.

[11] Baker PK, Drew MGB, Meehan MM (2000). The first iodine induced halide abstraction reaction: synthesis and molecular structure of 
*fac*-[Re(CO)_3_(NCMe)_3_] ⋅ BrI_2_. *Inorganic Chemistry Communications*.

[12] Domercq B, Devic T, Fourmigué M, Auban-Senzier P, Canadell E (2001). Hal ⋯ Hal interactions in a series of three isostructural salts of halogenated tetrathiafulvalenes. Contribution of the halogen atoms to the HOMO-HOMO overlap interactions. *Journal of Materials Chemistry*.

[13] Williams JM, Wang HH, Beno MA (1984). Ambient-pressure superconductivity at 2.7 K and higher temperatures in derivatives of (BEDT-TTF)_2_IBr_2_: synthesis, structure, and detection of superconductivity. *Inorganic Chemistry*.

[14] Endres H, Hiller M, Keller HJ (1985). *Zeitschrift für Naturforschung, Section B*.

[15] Shibaeva RP, Lobkovskaya RM, Simonov MA, Yagubskii EB, Ignat'ev AA (1986). *Crystallography Reports*.

[16] Parlow A, Hartl H (1985). Syntheses and structure analyses of polyhalides in the system iodine/bromine. *Zeitschrift für Naturforschung*.

[17] Svensson PH, Kioo L (2000). A vibrational spectroscopic, structural and quantum chemical study of the triiodide ion. *Journal of the Chemical Society, Dalton Transactions*.

[18] Gardberg AS, Yang S, Hoffman BM, Ibers JA (2002). Synthesis and structural characterization of integrally oxidized, metal-free phthalocyanine compounds: [H_2_(pc)][IBr_2_] and [H_2_(pc)]_2_[IBr_2_]Br ⋅ C_10_H_7_Br. *Inorganic Chemistry*.

[19] Terzis A, Papavassiliou G, Kobayashi H, Kobayashi A (1989). Structure of the conducting salt of pyrazinoethylenedithiotetrathiafulvalene (PEDTTTF): α−(PEDTTTF)_2_IBr_2_, at 98 K. *Acta Crystallographica, Section C*.

[20] Terzis A, Psycharis V, Hountas A, Papavassiliou G (1988). *Acta Crystallographica, Section C*.

[21] Honda K, Goto M, Kurahashi M, Anzai H, Tokumoto M, Ishiguro T (1988). The crystal structure of a charge-transfer complex of tetrakis(methylthio)tetrathiafulvalenium dibromoiodate, TMT-TTF-IBr_2_. *Bulletin of the Chemical Society of Japan*.

[22] Bigoli F, Deplano P, Mercuri ML (1998). Novel oxidation and reduction products of the neutral nickel-dithiolene Ni(Pr_2_
^*i*^timdt)_2_ (Pr_2_
*^i^*timdt is the monoanion of 1,3-diisopropylimidazolidine-2,4,5-trithione). *Inorganica Chimica Acta*.

[23] Raptis RG, Murray HH, Staples RJ, Porter LC, Fackler JP (1993). Structural isomers of [Au(CH_2_)_2_PPh_2_]_2_Br_4_ 2. Crystal structures of *cis/cis*-[Au(CH_2_)_2_PPh_2_]_2_Br_4_ and the cationic A-frame [(*μ*−Br)(Au(CH_2_)_2_PPh_2_)_2_Br_2_][IBr_2_]. *Inorganic Chemistry*.

[24] Kini AM, Parakka JP, Geiser U (1999). Tetraalkyl- and dialkyl-substituted BEDT-TTF derivatives and their cation-radical salts: synthesis, structure, and properties. *Journal of Materials Chemistry*.

[25] Cau L, Deplano P, Marchiò L (2003). New powerful reagents based on dihalogen/N,N′- dimethylperhydrodiazepine-2,3-dithione adducts for gold dissolution: the IBr case. *Dalton Transactions*.

[26] Shibaeva RP, Rozenberg LP, Korotkov VE (1989). *Crystallography Reports*.

[27] Lykova EO, Chernov'yants MS, Kazheva ON, Chekhlov AN, D'yachenko OA (2004). The synthesis, structure, and stability of diiodobromides of N-substituted isoquinolinium derivatives. *Zhurnal Fizicheskoj Khimii*.

[28] Akutagawa T, Abe Y, Hasegawa T (1999). Crystal structures and optical properties of cation radical salts of a tetrathiafulvalene trisannulated macrocycle. *Journal of Materials Chemistry*.

[29] Blake AJ, Gilby LM, Gould RO, Lippolis V, Parsons S, Schröder M (1998). Macrocyclic thioether complexes of palladium with dibromoiodide anions. *Acta Crystallographica, Section C*.

[30] Godfrey SM, McAuliffe CA, Pritchard RG, Sarwar S (1997). Structural characterization of the diorganoselenium interhalogen compounds R_2_SeIBr (R=Ph or Me) and the ionic compound [Me_3_Se][IBr_2_]. *Journal of the Chemical Society, Dalton Transactions*.

[31] Bekaert A, Barberan O, Kaloun EB (2003). Crystal structure of tetrakis(N,N-dimethylacetamide-O)borane tris(dibromoiodide), {B[CH_3_CON(CH_3_)_2_)]_4_}(Br_2_I)_3_. *Zeitschrift für Kristallographie: New Crystal Structures*.

[32] Naito T, Tateno A, Udagawa T (1994). Synthesis, structures and electrical properties of the charge-transfer salts of 4,5-ethylenedithio-4′,5′-(2-oxatrimethylenedithio)diselenadithiafulvalene (EOST) with linear anions [I_3_
^−^
,IBr_2_
^−^,ICl_2_
^−^,I_2_Br^−^,AuBr_2_
^−^,Au(CN)_2_
^−^]. *Journal of the Chemical Society, Faraday Transactions*.

[33] Minkwitz R, Berkei M, Ludwig R (2001). Preparation and crystal structure of tetraphenylphosphonium triiodotetrabromide [PPh_4_][I_3_
Br_4_]. *Inorganic Chemistry*.

[34] Breneman GL

[35] Dautel OJ, Fourmigué M, Canadell E (2001). Activation of C-H ⋯ halogen (Cl, Br, and I) hydrogen bonds at the organic/inorganic interface in fIuorinated tetrathiafulvalenes salts. *Chemistry - A European Journal*.

[36] Wang HH, Montgomery LK, Geiser U (1989). Syntheses, structures, selected physical properties and band electronic structures of the bis(ethylenediseleno)tetrathiafulvalene salts, (BEDSe-TTF)_2_X, X^−^ = I_3_
^−^, AuI_2_
^−^, and IBr_2_
^−^. *Chemistry of Materials*.

[37] Terzis A, Hountas A, Papavassiliou GC, Hilti B, Pfeiffer J (1990). Structures and conductivities of the synthetic metal salts of ethylenedithiotetrathiafulvalene (EDTTTF) and ethylenedithiodiselenadithiafulvalene (EDTDSDTF): 211-(EDTTTF)_2_IBr_2_
, 211-(EDTDSDTF)_2_IBr_2_ and 212-(EDTTTF)_2_AuBr_2_. *Acta Crystallographica, Section C*.

[38] Bigoli F, Demartin F, Deplano P (1996). Synthesis, characterization, and crystal structures of new dications bearing the -Se-Se- bridge. *Inorganic Chemistry*.

[39] Müller U (1979). *Zeitschrift für Naturforschung, Section B*.

[40] Janczak J, Kubiak R (2003). Sandwich-type niobium(V) diphthalocyaninato complexes ‘stapled’ by two inter-ligand C—C σ-bonds. Synthesis and structural investigations of two new phthalocyaninato complexes: [NbPc_2_](IBr_2_) and [NbPc_2_](IBr_2_) ⋅ I_2_. *Polyhedron*.

[41] Korotkov VE, Shibaeva RP (1991). *Crystallography Reports*.

[42] Cristiani F, Demartin F, Devillanova FA, Isaia F, Lippolis V, Verani G (1994). Charge-transfer complexes of N-Methylthiazolidine-2(3H)-selone (1) and
N-Methylbenzothiazole-2(3H)-selone (2) with 
I_2_
and IBr: crystal structures of 11 ⋅ I_2_, 1.Br_0.75_,
2⋅ 2I_2_, and 2 ⋅ 2IBr. *Inorganic Chemistry*.

[43] Tateno A, Udagawa T, Naito T, Kobayashi H, Kobayashi A, Nogami T (1994). Crystal structures and electrical properties of the radical
salts of the unsymmetrical donor EOTT
[4,5-ethylenedithio-4′,5′-(2-oxatrimethylenedithio)tetrathiafulvalene]. *Journal of Materials Chemistry*.

[44] Hoskins BF, Robson R, Williams GA (1976). Complexes of binucleating ligands. VIII. The preparation, structure and properties of some mixed valence cobalt(II)—cobalt(III) complexes of a macrocyclic
binucleating ligand. *Inorganica Chimica Acta*.

[45] Wolmershauser G, Kruger C, Tsay Y-H (1982). *Chemische Berichte*.

[46] Bogaard MP, Rae AD (1982). *Crystal Structure Communications*.

[47] Bricklebank N, Skabara PJ, Hibbs DE, Hursthouse MB, Malik KMA (1999). Reaction of thiones with dihalogens; comparison of the solid state structures of 4,5-bis(methylsulfanyl)-1,3-dithiole-2-thione-diiodine, -dibromine and -iodine monobromide. *Journal of the Chemical Society, Dalton Transactions*.

[48] Maas G, Hoge R (1980). *Liebigs Annalen*.

[49] Andresen O, Rømming C (1962). *Acta Chemica Scandinavica*.

[50] Boyle PD, Cross WI, Godfrey SM, McAuliffe CA, Pritchard RG, Teat SJ (1999). Reaction of dimethylselenourea and selenourea with dibromine to produce selenourea-dibromine, the ‘T’-shaped 1:1 molecular adduct N,N-dimethyl-2-selenourea-dibromine, its solvent of crystallisation-containing analogue and the unusual ionic compound. *Journal of the Chemical Society, Dalton Transactions*.

[51] Burford N, Chivers T, Rao MNS, Richardson JF (1984). Oxidative addition of halogens to 1,3- and 1,5-(Ph_2_PN)_2_(SN)_2_: X-ray crystal structure of 1,5-(Ph_2_PN)_2_(NSBr)_2_ and a comparison of the crystal structures of the 12-membered ring 1,3,7,9-(Ph_2_PN)_2_(SN)_2_ and the corresponding dication (Ph_2_PN)_4_
(SN)_2_
^2+^. *Inorganic Chemistry*.

[52] Abramovitch RA, Ooi GHC, Sun H-L, Pierrot M, Baldy A, Estienne J (1984). *Chemical Communications*.

[53] Estienne J (1986). Structure d'un dérivé de la saccharine: le tribromure de bis(diéthylamino)-1,3 diméthyl-2,4 (trioxo-1,1,3, 2H-benzothiazol-1λ^6^,3 yl-2)-2 cyclobuténium. La géométrie de l'ion tribromure. *Acta Crystallographica, Section C*.

[54] Wieghardt K, Backes-Dahmann G, Herrmann W, Weiss J (1984). A Binuclear, mixed-valence Mo^VI/V^-complex; the crystal structure of [(C_9_H_21_N_3_)_2_Mo_2_
^VI^O_5_](Br_3_)_2_. *Angewandte Chemie International Edition*.

[55] Allwood BL, Moysak PI, Rzepa HS, Williams DJ (1985). *Chemical Communications*.

[56] Slebocka-Tilk H, Ball RG, Brown RS (1985). The question of reversible formation of bromonium ions during the course of electrophilic bromination of olefins. 2. The crystal and molecular structure of the bromonium ion of adamantylideneadamantane. *Journal of the American Chemical Society*.

[57] Bruce MI, Humphrey MG, Koutsantonis GA, Nicholson BK (1985). Reactions of transition metal acetylide complexes IV. Synthesis and X-ray structure of a bromovinylidene complex, [Ru{C=CBr(C_6_
H_4_Br-4)}(PPh_3_)_2_(*η*−
C_5_H_5_)][Br_3_] ⋅ CHCl_3_. *Journal of Organometallic Chemistry*.

[58] Cotton FA, Lewis GE, Schwotzer W (1986). Preparation and properties of the tribromide of trans-dibromotetrakis(acetonitrile)vanadium(III), 
[VBrI_2_(CH_3_CN)_4_]Br_3_. A symmetric tribromide ion. *Inorganic Chemistry*.

[59] Endres H (1986). *Zeitschrift für Naturforschung, Section B*.

[60] Boeyens JCA, Denner L, Howard AS, Michael JP (1986). *South African Journal of Chemistry*.

[61] Detty MR, Luss HR (1986). Tellurapyrylium dyes. 3. Oxidative halogen addition and tellurium-halogen exchange. *Organometallics*.

[62] Aragoni MC, Arca M, Devillanova FA (2005). Self-assembly of supramolecular architectures based on polybromide anions: crystal structure of 
[(H_4_tppz^4+^)(Br^−^)_2_(Br_4_
^2^
^−^)] [tppz = tetra(2-pyridyl)pyrazine]. *Inorganic Chemistry Communications*.

[63] Vaughan GBM, Mora AJ, Fitch AN, Gates PN, Muir AS (1999). A high resolution powder X-ray diffraction study of the products of reaction of dimethyl sulfide with bromine; crystal and molecular structures of (CH_3_)_2_
SBr_n_(n = 2,2.5 or 4). *Journal of the Chemical Society, Dalton Transactions*.

[64] Vogt H, Wulff-Molder D, Ritschl F, Mücke M, Skrabei U, Meisel M (1999). Tris(dialkylamino)benzylphosphonium bromides - Phosphonium salts with three N atoms configurated nearly planar. *Zeitschrift für anorganische und allgemeine Chemie*.

[65] Zimmerman HE, Wang P (2002). Inter- and intramolecular stereoselective protonation of enols. *Journal of Organic Chemistry*.

[66] Bashore CG, Samardjiev IJ, Bordner J, Coe JW (2003). Twisted amide reduction under Wolff-Kishner conditions: synthesis of a benzo-1-aza-adamantane derivative. *Journal of the American Chemical Society*.

[67] Nolte J, Neubauer P, Vogt H, Meisel M (1999). Syntheses and crystal structures of tris(di-n-propylamino)-p-bromobenzyl-phosphonium bromides: [(C_3_H_7_)_2_N]_3_PCH_2_C_6_H_4_Br^+^Br^−^ and [(C_3_
H_7_)_2_
N]_3_PCH_2_C_6_H_4_
Br^+^Br_3_
^−^. *Zeitschrift für Naturforschung, Section B*.

[68] Ruiz J, Riera V, Vivanco M, García-Granda S, Díaz MR (1998). Reactivity of bis(diphenylphosphino)methanide complexes of manganese(I) toward halogens and Pseudohalogens. *Organometallics*.

[69] Cross WI, Godfrey SM, Jackson SL, McAuliffe CA, Pritchard RG (1999). The reaction of the tertiary phosphine sulfides R_3_PS(R=Ph,Me_2_ or C_6_H_11_) with X_2_(X_2_
=I_2_,Br_2_, IBr or ICl); structural characterization of the CT complexes (Me_2_N)_3_PSI_2_ and Ph_3_PS(I_0.89_Br_0.11_)Br and the ionic compound [{(Me_2_N)_3_PS}_2_S]^2+^
2[Br_3_]^−^. *Journal of the Chemical Society, Dalton Transactions*.

[70] Rovnaník P, Kapička L, Taraba J, Černík M (2004). Base-induced dismutation of POCl_3_ and POBr_3_: synthesis and structure of ligand-stabilized dioxophosphonium cations. *Inorganic Chemistry*.

[71] Pickardt J, Schumann H, Mohtachemi R (1990). Structure of decamethylferrocenium tribromide. *Acta Crystallographica, Section C*.

[72] Coleman AW, Means CM, Bott SG, Atwood JL (1990). Air-stable liquid clathrates. 1. Crystal structure of [NBu_4_][Br_3_
] and reactivity of the [NBu_4_][Br_3_] ⋅ 5C_6_H_6_ liquid clathrate. *Journal of Chemical Crystallography*.

[73] Liu J-C, Ishizuka T, Osuka A, Furuta H (2003). Modulation of axial coordination in N-confused porphyrin-antimony(v) dibromide complex by proton stimulus. *Chemical Communications*.

[74] Bekaert A, Lemoine P, Viossat B, Jouan M, Gemeiner P, Brion JD (2005). Synthesis, crystal structure, IR and Raman properties of 1,2-diacetamidocyclohexane and its complexes with ZnBr_2_ and HBr_3_. *Journal of Molecular Structure*.

[75] Atwood JL, Junk PC, May MT, Robinson KD (1994). Synthesis and X-ray structure of [H_3_
O^+^⋅18-crown-6] [Br-Br-Br^−^]; a compound containing both H_3_O^+^ and a linear and symmetrical Br_3_
^−^ ion crystallized from aromatic solution. *Journal of Chemical Crystallography*.

[76] Janickis V (1999). Syntheses and crystal structures of phenyltrimethylammonium salts of a mixed Hexabromoselenate/tellurate(IV), [C_6_H_5_(CH_3_)_3_N]_2_[Se_0.75_Te_0.25_Br_6_], and a mixed catena-poly[(Di-*μ*-bromobis{tetrabromoselenate/tellurate(IV)}
)-*μ*-bromine], [C_6_H_5_(CH_3_)_3_
N]_2n_. *Acta Chemica Scandinavica*.

[77] Bekaert A, Barberan O, Kaloun EB (2001). Crystal structure of N-methylpyrrolidone-2-one-N-methylpyrrolidine-2-onium perbromide, C_10_H_19_Br_3_N_2_O_2_. *Zeitschrift für Kristallographie: New Crystal Structures*.

[78] Hubig SM, Lindeman SV, Kochi JK (2000). Charge-transfer bonding in metal-arene coordination. *Coordination Chemistry Reviews*.

[79] Lawton SL, Hoh DM, Johnson RC, Knisely AS (1973). Crystal structure of 4-methylpyridinium nonabromoantimonate(V),
(4−C_6_
H_7_NH)_2_Sb^V^Br_9_. *Inorganic Chemistry*.

[80] Spandl J, Daniel C, Brüdgam I, Hartl H (2003). Synthesis and structural characterization of redox-active dodecamethoxoheptaoxohexavanadium clusters. *Angewandte Chemie International Edition*.

[81] Le Gall B, Conan F, Cosquer N (2001). Unexpected behaviour of copper(I) towards a tridentate Schiff base: synthesis, structure and properties of new Cu(I)-Cu(II) and Cu(II) complexes. *Inorganica Chimica Acta*.

[82] Vogt H, Quaschning V, Ziemer B, Meisel M (1997). Synthesis and crystal structures of tris(diethylamino)benzylphosphonium bromides: [(C_2_H_5_)_2_
N]_3_PCH_2_C_6_
H_5_
^+^Br^−^
⋅CH_3_CN and [(C_2_H_5_)_2_
N]_3_PCH_2_C_6_H_5_
^+^
Br_3_
^−^. *Zeitschrift für Naturforschung, Section B*.

[83] Hughes BB, Haltiwanger RC, Pierpont CG, Hampton M, Blackmer GL (1980). Synthesis and structure of a 12-crown-4 sandwich complex of manganese(II), bis(1,4,7,10-tetraoxacyclododecane)manganese(II) tribromide. *Inorganic Chemistry*.

[84] Calleri M, Ferguson G (1972). *Crystal Structure Communications*.

[85] Lawton SL, Jacobson RA (1968). The crystal structure of *α*-Picoliniunn Nonabrsmoantimonate(V), (C_6_H_7_NH)_2_Sb^v^Br_9_. *Inorganic Chemistry*.

[86] Brenčič JV, Chernega AN, Rotar R (1999). Structural identification of rans-[Mo^III^Br_2_py_4_]
Br_3_(py=pyridine,C_5_H_5_N). *Acta Chimica Slovenica*.

[87] Breneman GL

[88] Lawton SL, McAfee ER, Benson JE, Jacobson RA (1973). Crystal structure of quinolinium hexabromoantimonate(V) tribromide, (C_9_H_7_NH)_2_Sb^v^Br_9_. *Inorganic Chemistry*.

[89] Robertson KN, Bakshi PK, Cameron TS, Knop O (1997). Polyhalide anions in crystals. 3. The Br_8_
^2−^ anion in diquinuclidinium octabromide, the crystal structures of Me_4_PBr_3_ and quinuclidinium tribromide, and Ab initio calculations on polybromide anions. *Zeitschrift für anorganische und allgemeine Chemie*.

[90] Bock H, Rauschenbach A, Näther C, Kleine M, Bats JW (1996). Einkristall-molekülstrukturen 107^1,2^: strukturänderungen von thianthren, 2,3,6,7-tetramethoxythianthren und 2,3,6,7-tetramethoxyselenanthren bei komplexbildung mit elektronenakzeptoren sowie bei einelektronenoxidation zu den radikalkationen. *Phosphorus, Sulfur and Silicon and Related Elements*.

[91] Hübner J, Wulff-Molder D, Vogt H, Meisel M (1997). Synthesis and crystal structures of (Benzyl)triphenylphosphonium Bromides, [C_6_
H_5−_C*H*
_2_P(C_6_H_5_)_3_]^+^Br^−^ and [C_6_
H_5_
_−_CH_2_
P(C_6_H_5_)_3_]^+^Br_3_
^−^. *Zeitschrift für Naturforschung, Section B*.

[92] Gingl F, Strahle J (1989). *Zeitschrift für Naturforschung, Section B*.

[93] Gubin AI, Buranbaev MZh, Kostynyuk VP, Kopot' OI, Ii'in AI (1988). *Crystallography Reports*.

[94] Mikhailov VA, Yufit DS, Struchkov YuT (1992). *Russian Journal of General Chemistry*.

[95] Vogt H, Trijanov SI, Rybakov VB (1993). *Zeitschrift für Naturforschung, Section B*.

[96] Hauge S, Marøy K (1996). Syntheses and crystal structures of phenyltrimethylammonium salts of hexabromoselenate(IV), [C_6_H_5_(CH_3_)_3_N]_2_[SeBr_6_], and catena-poly[(Di-*μ*-bromobis{tetrabromotellurate(IV)})-*μ*-bromine], [C_6_
H_5_(CH_3_)_3_
N]_2n_[Se_2_Br_10_ ⋅ Br_2_]_n_. *Acta Chemica Scandinavica*.

[97] Rotar R, Leban I, Brenčič JV (1996). Trans-dichlorotetrakis(pyridine-N)-molybdenum(III) tribromide. *Acta Crystallographica, Section C*.

[98] Bakshi PK, James MA, Cameron TS, Knop O (1996). Polyhalide anions in crystals. Part 1. Triiodides of the Me_4_N^+^, Me_4_P^+^, quinuclidinium, 1-azoniapropellane, and 1,4-diazoniabicyclo[2.2.2]octane (DabcoH_2_
^2+^) cations, and 1,10-phenanthrolinium(1+) tribromide. *Canadian Journal of Chemistry*.

[99] Field LD, Hambley TW, He T, Humphrey PA, Lindall CM, Masters AF (1996). The syntheses of [M(C_5_PH_5_)_2_]^n+^
(n = 0,1) complexes of nickel, iron and chromium. The structures of the decaphenylmetallocenium cations of nickel and iron. *Australian Journal of Chemistry*.

[100] Ollis J, James VJ, Ollis D, Bogaard MP (1976). *Crystal Structure Communications*.

[101] Bogaard MP, Peterson J, Rae AD (1979). *Crystal Structure Communications*.

[102] Vogt H, Wulff-Molder D, Meisel M (1996). Synthesis and crystal structures of (p-bromobenzyl)triphenylphosphonium bromides, [(p-Br-C_6_H_4_CH_2_)P(C_6_H_5_)_3_]^+^Br^−^; [(p-Br-C_6_H_4_−CH_2_)P(C_6_
H_5_)_3_]^+^Br_2_
^−^. *Zeitschrift für Naturforschung, Section B*.

[103] Bekaert A, Barberan O, Kaloun EB (2002). *Zeitschrift für Kristallographie: New Crystal Structures*.

[104] Markovskii LN, Pashinnik VE, Tovstenko VI (1991). *Russian Journal of Organic Chemistry*.

[105] Zürcher S, Petrig J, Gramlich V (1999). Charge-transfer salts of octamethylferrocenyl thioethers with organic acceptors (TCNQ and TCNQF_4_) and trihalides (Br_3_
^−^ and I_3_
^−^
). Synthesis, structure, and physical properties. *Organometallics*.

[106] Bekaert A, Barberan O, Kaloun EB (2002). Crystal structure of hexakis(N,N-dimethylformamide-O)aluminium(III) tris(tribromide), Al[(CH_3_)_2_N(CH)O]_6_(Br_3_)_3_. *Zeitschrift für Kristallographie: New Crystal Structures*.

[107] Arnáiz FJ, Miranda MJ, Aguado R, Maháa J, Maestro MA (2002). Uranyl polyhalides. Molecular structure of [UO_2_(OAsPh_3_)_4_]
(Br_3_)_2_ and [UO_2_(OPPh_3_)_4_]
(I_3_)_2_. *Polyhedron*.

[108] Kuhn N, Bohnen H, Henkel G (1994). *Zeitschrift für Naturforschung, Section B*.

[109] Vogt H, Frauendorf C, Fischer A, Jones PG (1995). *Zeitschrift für Naturforschung, Section B*.

[110] Wang Y-Q, Wang Z-M, Liao C-S, Yan C-H (1999). Bis(1,10-phenanthrolin-1-ium) chlorodiiodide(1-) dichloroiodide(1-). *Acta Crystallographica, Section C*.

[111] Shilov GV, Kazheva ON, D'yachenko OA (2002). The synthesis, structure, and stability of N-cetylpyridinium interhalides: an experimental and quantum-chemical study. *Zhurnal Fizicheskoj Khimii*.

[112] Kobayashi H, Kato R, Kobayashi A (1986). The crystal structure of *β*′-(BEDT-TTF)_2_ICl_2_. A modification of the organic superconductor, *β*-(BEDT−TTF)_2_I_3_. *Chemistry Letters*.

[113] Laukhina E, Vidal-Gancedo J, Khasanov S (2000). New organic conductor and a novel structural phase transition in the BEDT-TTF trihalide family. *Advanced Materials*.

[114] Laukhina E, Vidal-Gancedo J, Laukhin V (2003). Multistability in a BEDT-TTF based molecular conductor. *Journal of the American Chemical Society*.

[115] Archer EM, van Schalkwyk TGD (1953). The crystal structure of benzene iododichloride. *Acta Crystallographica*.

[116] Carey JV, Chaloner PA, Hitchcock PB, Neugebauer T, Seddon KR (1996). *Journal of Chemical Research*.

[117] Wang Z-M, Wang Y-Q, Liao C-S, Yan C-H (1999). 1,10-Phenanthrolinium(2+) dichloroiodide(1-) chloride. *Acta Crystallographica, Section C*.

[118] Parlow A, Hartl H (1979). 2,2′-Bichinolinium-dijodtrichlorid. *Acta Crystallographica, Section B*.

[119] Minkwitz R, Berkei M (1999). A new method for preparation and crystal structure of (trifluoromethyl)iodine dichloride. *Inorganic Chemistry*.

[120] Kobayashi H, Kato R, Kobayashi A (1986). *Chemistry Letters*.

[121] Emge TJ, Wang HH, Leung PCW (1986). New cation-anion interaction motifs, electronic band structure, and electrical behavior in *β*-(ET)_2_X salts (X=
IC1_2_
^−^ and BrIC1^−^). *Journal of the American Chemical Society*.

[122] Shibaeva RP, Rozenberg LP, Yagubskii EB, Ignat'ev AA, Kotov AI (1987). *Doklady Akademii Nauk SSSR*.

[123] Laukhina E, Tkacheva V, Chekhlov A (2004). Polymorphism of a new bis(ethylenedithio)tetrathiafulvalene (BEDT-TTF) based molecular conductor; novel transformations in metallic BEDT-TTF layers. *Chemistry of Materials*.

[124] Chekhlov AN (2004). *Journal of Structural Chemistry*.

[125] Nikiforov VA, Karavan VS, Miltsov SA (2003). *ARKIVOC*.

[126] Bozopoulos A, Kavounis CA (2004). *Zeitschrift für Kristallographie: New Crystal Structures*.

[127] Williams JM, Emge TJ, Firestone MA (1987). *Molecular Crystals and Liquid Crystals*.

[128] Shibaeva RP, Rozenberg LP, Simonov MA, Kushch ND, Yagubskii EB (1988). *Crystallography Reports*.

[129] Bandoli G, Clemente DA, Nicolini M (1978). Crystal and molecular structure of hexadecyltrimethylammonium dichloroiodide, an antiseptic agent. *Journal of Chemical Crystallography*.

[130] Grebe J, Geiseler G, Harms K, Dehnicke K (1999). Synthesis and crystal structure of PPh_4_
[PhICl_3_]. *Zeitschrift für Naturforschung, Section B*.

[131] Belaj F (1995). Structure and thermal motion of tetrakis(trichlorophosphazeno)phosphonium dichloroiodate(I), [P(NPCl_3_)_4_]^+^[ICl_2_]^−^.2[(CCl_4_)_*x*_(CHCl_3_)_1_
_−_
_*x*_],= 0.67(2). *Acta Crystallographica, Section B*.

[132] March FC, Ferguson F (1975). Stereochemistry of some organic derivatives of group Vb elements. Part VIII. Crystal and molecular structure of *μ*-chloro-bis[hydroxytriphenylarsenic](1+) dichloroiodate(1–). *Journal of the Chemical Society, Dalton Transactions*.

[133] Zhdankin VV, Callies JA, Hanson KJ, Bruno J (1999). New alkyliodonium derivatives stabilized by ammonium or phosphonium groups. *Tetrahedron Letters*.

[134] Sosonyuk SE, Bulanov MN, Leshcheva IF, Zyk NV (2002). *Russian Chemical Bulletin*.

[135] Visser GJ, Vos A (1964). The length of the I-Cl bond in tetramethylammonium dichloroiodide. *Acta Crystallographica*.

[136] El Essawi M, Tebbe K-F (1998). Studies on polyhalides, XXXIV. Cesium(18-crown-6)dichloroiodate, [Cs(C_12_H_24_O_6_]ICl_2_ICl_2_. *Zeitschrift für Naturforschung, Section B*.

[137] Grebe J, Weller F, Dehnicke K (1997). *Zeitschrift für Kristallographie: New Crystal Structures*.

[138] Caira MR, de Wet JF (1981). 4,4,5,5-Tetramethyl-2-phenyl-1, 3-dioxolan-2-ylium dichloroiodate(I). *Acta Crystallographica, Section B*.

[139] Rømming C (1958). *Acta Chemica Scandinavica*.

[140] Lang ES, Burrow RA, Diniz J (2000). *β*-Pyridinium dichloroiodide. *Acta Crystallographica, Section C*.

[141] Chitsaz S, Folkerts H, Grebe J (2000). Crystal structures of a series of compounds with cations of the type [R_3_PNH_2_]^+^, [R_3_PN(H)SiMe_3_]^+^, and [R_3_PN(SiMe_3_)_2_]^+^. *Zeitschrift für anorganische und allgemeine Chemie*.

[142] Hauge S, Marøy K (1996). Syntheses and crystal structures of phenyltrimethylammonium salts of hexachlorotellurate(IV), [C_6_
H_5_(CH_3_)_3_N] [TeCl_6_], catena-poly[(Di-*μ*-chlorobis{tetrachlorotellurate (IV)})-*μ*-bromine], [C_6_
H_5_(CH_3_l_3_N] [Te_2_Cl_10_ ⋅ Br_2_]_*n*′_. *Acta Chemica Scandinavica*.

[143] Baenziger NC, Buckles RE, Simpson TD (1967). Complexes of p-anisylethylenes. III. Crystal structure of the dichloroiodate(I) salt of the tetra-p-anisylethylene dication. *Journal of the American Chemical Society*.

[144] Irsen SH, Dronskowski R (2002). Synthesis and X-ray crystal structure determination of thiotrithiazyl iododichloride, S_4_N_3_ICl_2_. *Zeitschrift für Naturforschung, Section B*.

[145] Schultz AJ, Geiser U, Kini AM (1988). Preparation and characterization of two structural phases of (EPT)_2_ICl_2_. *Synthetic Metals*.

[146] Gubin AI, Ill'in AI, Pugina EG, Kostinyuk VP, Buranbaev MZh (1990). *Crystallography Reports*.

[147] Carmalt CJ, Norman NC, Farrugia LJ (1993). The syntheses and structures of two large iodoantimonate anions. *Polyhedron*.

[148] Boyle PD, Cross WI, Godfrey SM, McAuliffe CA, Pritchard RG, Teat S (1999). The reaction of N-methylbenzothiazole-2-selone with the interhalogens iodine monobromide and iodine monochloride. *Journal of the Chemical Society, Dalton Transactions*.

[149] Minkwitz R, Berkei M (2001). Crystal structure of Tetraphenylphosphonium dichloroiodate(I) [PPh_4_]
[ICl_2_]. *Zeitschrift für Naturforschung, Section B*.

[150] Protasiewicz JD (1995). *Chemical Communications*.

[151] Mishra AK, Olmstead MM, Ellison JJ, Power PP (1995). Detailed structural characterization of the polyvalent iminoiodinanes ArINTs (Ar=C_6_
H_5_ or 2,4,6-Me_3_C_6_H_2_; Ts = SO_2_C_6_H_4_-4-Me) and the aryldichloroiodinane 2,4,6-i-Pr_3_C_6_H_2_ICl_2_. *Inorganic Chemistry*.

[152] Grebe J, Harms GK, Weller F, Dehnicke K (1995). Reaktionen silylierter Phosphanimine mit Iodmonochlorid und Iodtrichlorid. Die Kristallstrukturen von [Me_3_SiNPMe_3_ ⋅ ], [Ph_3_PNCl ⋅ ICl] und [Me_3_PN(H)PMe_3_][ICl_2_]_2_. *Zeitschrift für anorganische und allgemeine Chemie*.

[153] Gabes W, Olie K (1974). *Crystal Structure Communications*.

[154] Boere RT, Cordes AW, Oakley RT, Reed RW (1985). *Chemical Communications*.

[155] Boeré RT, Cordes AW, Craig SL, Oakley RT, Reed RW (1987). Stereochemistry of oxidation of 1,5,2,4,6,8-dithiatetrazocines. Preparation and crystal structures 
of 
[(Me_2_E)_2_C_2_
E_2_E_2_
Cl]^+^X^−^(X^−^
= PF_6_
^−^,Cl_6_
^−^)
and (Me_2_N)_2_
C_2_N_4_
S_2_(O)_2_[N(CF_3_
)_2_]_2_. *Journal of the American Chemical Society*.

[156] Chivers T, Richardson JF, Smith NRM (1985). Reaction of Dimethylcyanamide with sulfur dichloride:
X-ray crystal structures of the
N,N′
- (Chlorosulfoniumylidene)bis(N^1^,N^1^-dimethylchloroformamidine) complexes
[(Me_2_NC(Cl)N)_2_
SCl]^+^X^−^(X^−^=Cl^−^,Cl_3_
^−^) and the Hydrolysis product
[Me_2_NC(Cl)NH_2_
]^+^Cl^−^ ⋅ H_2_O. *Inorganic Chemistry*.

[157] Bogaard MP, Peterson J, Rae AD (1981). Tetraphenylarsonium trichloride. *Acta Crystallographica, Section B*.

[158] Taraba J, Zak Z (2003). Diphenyldichlorophosphonium trichloride-chlorine solvate 1:1, [PPh_2_Cl_2_]^+^Cl_3_
^−^ ⋅ Cl_2_: an ionic form of diphenyltrichlorophosphorane. Crystal structures. *Inorganic Chemistry*.

[159] Gorge A, Patt-Siebel U, Müller U, Dehnicke K (1988). *Zeitschrift für Naturforschung, Section B*.

[160] Jansen M, Strojek S (1995). *Zeitschrift für Naturforschung, Section B*.

[161] Bondi A (1964). van der Waals volumes and radii. *The Journal of Physical Chemistry*.

[162] Becker JY, Bernstein J, Dayan M, Shahal L (1992). *Chemical Communications*.

[163] Kumar RK, Aravamudan G, Seshasayee M, Sivakumar K, Fun H-K, Goldberg I (1998). Synthesis and structural characterization of two interesting sandwich and double sandwich type mixed-valent tellurium-dithiocarbamate complexes. *Polyhedron*.

[164] Foss O, Henjum J, Maartmann-Moe K, Maroy K (1987). *Acta Chemica Scandinavica*.

[165] Sekar P, Ibers JA (2003). Synthesis and characterization of HN(SP^i^PR_2_
)(SePPh_2_) and [Te{N(SP^i^Pr_2_)(SePPh_2_
)}_2_]. *Inorganic Chemistry*.

[166] Dodds CA, Kennedy AR, Reglinski J, Spicer MD (2004). Pushing the frontiers of hard and soft scorpionate chemistry. *Inorganic Chemistry*.

[167] Canseco-Melchor G, García-Montaivo V, Toscano RA, Cea-Olivares R (2001). New mixed ligand organotellurium(IV) compounds containing dithiocarbamates and the more flexible imidotetraphenyldithiodiphosphinates. The crystal structures of C_8_H_8_
Te(S_2_CNEt_2_)[(SPPh_2_)_2_
N] ⋅ H_2_
O,C_8_
H_8_Te(S_2_
CNC_5_H_10_
)[(SPPh_2_)_2_
N], and C_8_H_8_
Te(S_2_
CNC_4_
H_8_
S)[(SPPh_2_)_2_
N]. *Zeitschrift für anorganische und allgemeine Chemie*.

[168] Ase K (1969). *Acta Chemica Scandinavica*.

[169] von Deuten K, Schnabel W, Klar G (1979). *Crystal Structure Communications*.

[170] Müller U, Bubenheim W (1999). Synthese und Kristallstrukturen von (NEt_4_)_2_[TeS_3_], (NEt_4_)_2_[Te(S_5_)(S_7_)] und (NEt_4_)_4_[Te(S_5_)_2_][Te(S_7_)_2_]. *Zeitschrift für anorganische und allgemeine Chemie*.

[171] Rout GC, Seshasayee M, Aravamudan G, Radha K (1984). Molecular structure of thiocyanatotris (2,2′-iminodiethanoldithiocarbamato) tellurium(IV) monohydrate. *Journal of Chemical Crystallography*.

[172] Rout GC, Seshasayee M, Aravamudan G, Sowrirajan S (1984). Synthesis and structure of *trans*-bis[2(3*H*)-benzimidazolethione]bis(thiourea)tellurium(II) chloride, [Te(CH_4_
N_2_
S)_2_(C_7_
H_6_N_2_
S)_2_]Cl_2_. *Acta Crystallographica, Section C*.

[173] Novosad J, Tornroos KW, Necas M, Slawin AMZ, Woollins JD, Husebye S (1999). Reaction of large-bite ligands with various tellurium compounds. Synthesis and structural characterization of [Te_2_(*μ* −Cl)_2_{(SPPh_2_)_2_
*N*}_2_], [(4
−
MeOC_6_H_4_
TeCl_3_)_2_{*μ* −Ph_2_
P(S)CH_2_CH_2_
P(S)Ph_2_}] and [Te_2_(*μ* −Ph_2_PS_2_)_2_] representing novel types of tellurium complexes. *Polyhedron*.

[174] Birdsall DJ, Novosad J, Slawin AMZ, Woollins JD (2000). Synthesis and crystal structures of tellurium complexes containing imidophosphinate ligands. *Journal of the Chemical Society, Dalton Transactions*.

[175] Rout GC, Seshasayee M, Aravamudan G, Sowrirajan S (1984). Synthesis and structure of tetrakis(phenylenethiourea)tellurium(II) chloride dihydrochloride, C_28_H_26_N_8_S_4_Cl_4_Te. *Polyhedron*.

[176] Wieber M, Schmidt E, Burschka C (1985). Dimethyl-tellur-bis(alkylxanthogenate). *Zeitschrift für anorganische und allgemeine Chemie*.

[177] Rout GC, Seshasayee M, Aravamudan G, Sowrirajan S (1985). Crystal and molecular structure of tetrakis-(phenylenethiourea)tellurium(II) perchlorate hexahydrate, C_28_H_24_N_8_O_8_S_4_Cl_2_Te ⋅ _6_H_2_O. *Journal of Chemical Crystallography*.

[178] Valle G, Calogero S, Russo U (1980). *Crystal Structure Communications*.

[179] Elder RC, Marcuso T, Boolchand P (1977). The crystal structure of tetrakis(ethylenethiourea)tellurium(II) chloride dihydrate: a novel (+ − + −) square-planar conformer. *Inorganic Chemistry*.

[180] Ault HK, Husebye S (1978). *Acta Chemica Scandinavica, Series A*.

[181] Briand GG, Chivers T, Schatte G (2002). Redox chemistry of tellurium bis(tert-butylamido)cyclodiphosph(V)azane disulfide and diselenide systems: a spectroscopic and structural study. *Inorganic Chemistry*.

[182] Chadha RK, Drake JE, McManus NT, Quinlan BA, Sarkar AB (1987). Synthesis and characterization of aryldihalo(dialkyl dithiophosphato)tellurium(IV) and diarylbis(dialkyl dithiophosphato)tellurium(IV). Crystal structures of *p* − MoC_6_H_4_TeBr_2_[S_2_P(OMe)_2_] and Ph_2_Te[S_2_P(OMe)_2_]_2_. *Organometallics*.

[183] Foss O, Maartmann-Moe K, Maroy K (1986). *Acta Chemica Scandinavica, Series A*.

[184] Husebye S, Törnroos KW (2000). *trans*-Tetrachlorobis(*N*,
*N*
′;-dimethylimidazolidine-2-thione)tellurium(IV), a thiourea complex of tellurium with asymmetric Te-S bonds. *Acta Crystallographica, Section C*.

[185] Dakternieks D, di Giacomo R, Gable RW, Hoskins BF (1988). Crystal structures of Ph_2_Te(S_2_P(OEt)_2_)_2_ and of two modifications of Ph_2_Te(S_2_CNEt_2_)_2_. *Journal of Organometallic Chemistry*.

[186] Newton MG, King RB, Haiduc I, Silvestru A (1993). A unique supramolecular structure of catena-poly[bis(*μ*-diphenylphosphinodithioato)ditellurium(I) (Te–Te)], [Te_2_(S_2_PPh_2_)_2_]_n_, containing Te–Te ⋯ Te–Te ⋯ chains. *Inorganic Chemistry*.

[187] Drake JE, Khasrou LN, Mislankar AG, Ratnani R (1994). *Canadian Journal of Chemistry*.

[188] Williams DJ, Bevilacqua VLH, Morson PA, Pennington WT, Schimek GL, Kawai NT (2000). Main group metal halide complexes with sterically hindered thioureas. Part XVII. The crystal and molecular structures of two new tellurium chloride complexes with 1,3-dimethyl-2(3*H*)-imidazolethione. *Inorganica Chimica Acta*.

[189] Necas M, Novosad J, Husebye S (2001). Tellurium complexes with new, large-bite dithio ligands. The crystal structures of *cis*- and *trans*- [Te{Ph_2_P(S)-N-P(S)(OPh)_2_}_2_] and [(4-MeOC_6_H_4_TeCl_3_)_2_{*μ*−^*i*^Pr_2_P(S)-Fc-P(S)^i^Pr_2_}]. *Journal of Organometallic Chemistry*.

[190] Husebye S, Törnroos KW, Zhu H (2001). *Cis-trans* isomerism in square planar [TeCl_2_(stu)_2_] complexes with bulky substituted thiourea (stu) ligands syntheses and structures of four new tellurium(II) complexes. *Zeitschrift für anorganische und allgemeine Chemie*.

[191] Canseco-Melchor G, Garcia-Montalvo V, Toscano RA, Cea-Olivares R (2001). Synthesis and spectroscopic characterization of new mixed ligand organotellurium(IV) compounds employing dithiocarbamates and imidotetraphenyldithiodiphosphinates. Crystal structure of [C_4_H_8_Te(S_2_CNEt_2_){(SPPh_2_)_2_N}], [C_4_H_8_Te(S_2_CNC_5_H_10_){(SPPh_2_)_2_N}] and [C_4_H_8_Te(S_2_
CNC_4_
H_8_
S){(SPPh_2_)_2_N}]. *Journal of Organometallic Chemistry*.

[192] Bjornevag S, Husebye S, Maartmann-Moe K (1982). *Acta Chemica Scandinavica, Series A*.

[193] Dakternieks D, di Giacomo R, Gable RW, Hoskins BF (1988). Synthesis, NMR spectroscopic investigation, and crystal structures of 1,3-dihydro-2*λ*
^4^-benzotellurole-2,2-diyl bis( diethyldithiocarbamate), C_8_H_8_Te[S_2_CNEt_2_]_2_; S,S′- 1, 3-dihydro-2*λ*
_4_-benzotellurole-2,2-diyl *O*,*O*,*O*′,*O*′-tetraethyl bis(dithiophosphate), C_8_H_8_
Te[S_2_P(OEt)_2_]_2_; and 1, 3-dihydro-2*λ*
^4^-2X4-benzotellurole-2,2-diyl bis( 0-ethyl xanthate), C_8_H_8_Te[S_2_COEt]_2_. *Journal of the American Chemical Society*.

[194] Bailey JHE, Drake JE, Sarkar AB, Wong MLY (1989). Preparation and characterization of diphenylbis(*N*,*N*-dialkyldithiocarbamato)tellurium (IV) and chlorodiphenyl(*N*,*N*-dialkyldithiocarbamato)tellurium(IV). Crystal structures of Ph_2_
Te[S_2_
CNMe_2_]_2_, Ph_2_TeCl[S_2_CNEt_2_], and Ph_2_TeCl[S_2_
CN(*i*−Pr)_2_]. *Canadian Journal of Chemistry*.

[195] Husebye S, Maartmann-Moe K, Mikalsen O (1990). *Acta Chemica Scandinavica*.

[196] Bailey JHE, Drake JE, Wong MLY (1991). Preparation and characterization of a series of bromodiphenyl(*N*,*N*-dialkyldithiocarbamato)tellurium(IV) compounds where R=Me, Et, *i*-Pr, Bu, and of chlorodiphenyl(*N*,*N*-dibutyldithiocarbamato)tellurium(IV) and diphenylbis(*N*,*N*-dibutyldithiocarbamato)tellurium(IV). Crystal structure of Ph_2_
TeBr[S_2_CNEt_2_] and Ph_2_Te[S_2_CNBu_2_]_2_. *Canadian Journal of Chemistry*.

[197] Alcock NW, Culver J, Roe SM (1992). Secondary bonding. Part 15. Influence of lone pairs on co-ordination: comparison of diphenyl-tin(IV) and -tellurium(IV) carboxylates and dithiocarbamates. *Journal of the Chemical Society, Dalton Transactions*.

[198] Bogason JO, Dakternieks D, Husebye S, Maartmann-Moe K, Zhu H (1992). *Phosphorus, Sulfur, and Silicon and the Related Elements*.

[199] Husebye S, Maartmann-Moe K, Mikalsen O (1990). *Acta Chemica Scandinavica*.

[200] Drake JE, Drake RJ, Silvestru A, Yang J (1999). Tetraorganodichalcogenoimidodiphosphinato derivatives of dimethyltellurium(IV) compounds. Crystal structures of Me_2_Te[(SPPh_2_)_2_
N]_2_, Me_2_TeCl[(SPPh_2_)_2_N], Me_2_
Tel[(SPPh_2_)_2_
N
], and Me_2_TeCl[(OPPh_2_)(SPPh_2_)N]. *Canadian Journal of Chemistry*.

[201] Huang S-P, Dhingra S, Kanatzidis MG (1992). Synthesis and properties of the homo- and heteropolychalcogenide [A(Q_5_)_2_]^2−^ family (A=Te, Q=S, Se; A=Se, Q=Se). Crystal structures of (Ph_4_
P)_2_[Te(S_5_)_2_] and *β* − (Ph_4_
P)_2_[Se(Se_5_)_2_]. *Polyhedron*.

[202] Bubenheim W, Frenzen G, Müller U (1994). Synthese und Kristallstrukturen von (PPh_4_)_2_[Te(S_3_)] ⋅ 2CH_3_CN und (PPh_4_)_2_[Te(S_5_)_2_]. *Zeitschrift für anorganische und allgemeine Chemie*.

[203] Fleischer H, Schollmeyer D (2002). *Trans*-bis(1*H*-benzimidazole-1-thione−S)tetrachlorotellurium methanol disolvate. *Acta Crystallographica, Section E*.

[204] Drake JE, Ratnani R, Yang J (2001). Diphenyltellurium(IV) monothiocarbamates. The molecular structures of Ph_2_Te[SCONEt_2_]_2_, Ph_2_TeCl[SCONEt_2_], Ph_2_TeCl[SCONCH_2_(CH_2_)_2_(CH_2_)], and Ph_2_TeCl[SCONCH_2_(CH_2_)_3_(CH_2_)]. *Inorganica Chimica Acta*.

[205] Husebye S, Törnroos KW, Zhu H-Z (2001). Chlorotris(*N*,*N*
′-dicyclohexylthiourea-*S*)tellurium(II) chloride, a tellurium complex with a TeClS_3_ coordination sphere. *Acta Crystallographica, Section C*.

[206] Drake JE, Khasrou LN, Mislankar AG, Ratnani R (2000). The X-ray crystal structures of (O,O−2,3−dimethylbutylene and O,O−2−dimethylpropylene dithiophosphato)diphenyltellurium(IV) derivatives, Ph_2_Te[S_2_POCMe_2_CMe_2_O]_2_ and 2Ph_2_Te[S_2_POCH_2_
CMe_2_CH_2_O]_2_ ⋅ 2Ph_2_TeCl_2_ ⋅ CS_2_ and those of (O,O−2,3−dimethylbutylene and O,O-2-dimethylpropylene dithiophosphoric acids, HS_2_
POCMe_2_CMe_2_O and HS_2_POCH_2_CMe_2_CH_2_O. *Polyhedron*.

[207] Husebye S, Mughannam D, Törnroos KW (2003). *Cis-trans* isomerism in square planar [TeX_2_(stu)_2_] complexes (X=Br⋅,I⋅) with bulky substituted thiourea (stu) ligands. Syntheses and structures of four new tellurium(II) complexes. *Phosphorus, Sulfur, and Silicon and the Related Elements*.

[208] Foss O, Maroy K (1966). *Acta Chemica Scandinavica*.

[209] Hauge S, Vikane O (1975). *Acta Chemica Scandinavica, Series A*.

[210] Foss O, Husebye S, Törnroos KW, Fanwick PE (2004). Synthesis and X-ray crystal structures of six [TeL_4_][MF_6_] salts, where L is ethylene- or trimethylene-thiourea, and M is Si, Ge or Sn. Five Te(II) complexes with a novel type of structure linked together by unusual N—H—F hydrogen bonds. *Polyhedron*.

[211] Drake JE, Khasrou LN, Mislankar AG, Ratnani R (1999). Dimethyltellurium(IV) derivatives with mixed 1,1-dithio ligands. Crystal structures of Me_2_Te[S_2_CNMe_2_][S_2_COEt] and Me_2_Te[S_2_CNEt_2_][S_2_COMe]. *Canadian Journal of Chemistry*.

[212] Drake JE, Drake RJ, Khasrou LN, Mislankar AG, Ratnani R, Yang J (1996). Synthesis, spectroscopic and structural studies of *O*-methyl and *O*-isopropyl monothiocarbonate (monoxanthate) derivatives of dimethyl-and diphenyltellurium(IV). Crystal structures of Me_2_
Te[SCO_2_(i−Pr)]_2_, Ph_2_Te[SCO_2_(i−Pr)]_2_, Me_2_TeCl[SCO_2_Me], and Me_2_TeBr[SCO_2_(*i*−pr)]. *Canadian Journal of Chemistry*.

[213] Drake JE, Yang J (1997). Synthesis and spectroscopic characterization of pyrrolidyl and piperidyl dithioformate derivatives of dimethyltellurium(IV) compounds. Crystal structures of Me_2_
Te[S_2_CN(CH_2_)_3_CH_2_]_2_, Me_2_
Te[S_2_CN(CH_2_)_4_
CH_2_]_2_, Me_2_
Te{[S_2_
CN(CH_2_)_3_
CH_2_][S_2_
CN(CH_2_)_4_
CH_2_]}, Me_2_
TeCl[S_2_
CN(CH_2_)_3_
CH_2_], Me_2_
TeBr[S_2_
CN(CH_2_)_3_CH_2_], Me_2_
TeI[S_2_CN(CH_2_)_3_
CH_2_], Me_2_
TeCl[S_2_CN(CH_2_)_4_
CH_2_], and Me_2_
TeI[S_2_CN(CH_2_)_4_
CH_2_]. *Inorganic Chemistry*.

[214] García-Montalvo V, Zamora-Rosete MK, Gorostieta D, Cea-Olivares R, Toscano RA, Hernández-Ortega S (2001). Organotellurium(IV) derivatives of tetraphenyldichalcogenoimidodiphosphinates - the crystal and molecular structure of [C_4_
H_8_TeI{Ph_2_(Se)PNP(Se)Ph_2_
}], [C_4_H_8_TeI{Ph_2_
(S)PNP(S)Ph_2_}], [C_4_
H_8_Te{Ph_2_(S)PNP(S)Ph_2_
}_2_], [C_8_
H_8_
TeI{Ph_2_(S)PNP(S)Ph_2_}], and [O(TeC_4_
H_8_)_2_
{Ph_2_(O)PNP(O)Ph_2_}]_2_[I,I_3_
] representing a novel type of ring systems. *European Journal of Inorganic Chemistry*.

[215] Bailey JHE, Drake JE (1993). Synthesis and characterization of dimethyl- and dimethoxyphenylbis(
*N*,*N*-dialkyldithiocarbamato)tellurium(IV) and chlorodimethyl- and chlorodimethoxyphenyl-(*N*,*N*-dialkyldithiocarbamato)tellurium(IV). Crystal structures of Me_2_
Te[S_2_
CNMe_2_]_2_ and (*p*−MeOC_6_
H_4_)_2_
Te[S_2_
CNMe_2_]_2_. *Canadian Journal of Chemistry*.

[216] Novosad J, Lindeman SV, Marek J, Woollins JD, Husebye S (1998). Synthesis and structural characterization of [Te{(SePPh_2_)2
N}2] and [4-MeOPhTe{(SPPh_2_)_2_
N}]_2_. *Heteroatom Chemistry*.

[217] Husebye S, George JW (1969). Crystal and molecular structure of trans-tetrabromo- and trans-tetrachlorobis(tetramethylthiourea)tellurium(IV). *Inorganic Chemistry*.

[218] Ase K, Roti I (1974). *Acta Chemica Scandinavica, Series A*.

[219] Esperas S, George JW, Husebye S, Mikalsen O (1975). *Acta Chemica Scandinavica, Series A*.

[220] Foss O, Kjoge HM, Maroy K (1965). *Acta Chemica Scandinavica*.

[221] Ase K, Maartmann-Moe K, Solheim JO (1971). *Acta Chemica Scandinavica*.

[222] Foss O, Maroy K, Husebye S (1965). *Acta Chemica Scandinavica*.

[223] Ase K, Boyum K, Foss O, Maroy K (1971). *Acta Chemica Scandinavica*.

[224] Foss O, Lyssandtrae N, Maartmann-Moe K, Tysseland M (1973). *Acta Chemica Scandinavica*.

[225] Fosheim K, Foss O, Scheie A, Solheimsnes S (1965). *Acta Chemica Scandinavica*.

[226] Foss O, Maartmann-Moe K (1987). *Acta Chemica Scandinavica, Series A*.

[227] Anderson OP (1971). *Acta Chemica Scandinavica*.

[228] Ase K, Foss O, Roti I (1971). *Acta Chemica Scandinavica*.

[229] Foust AS (1980). Structures of bis(*μ*-thiourea-*S*)-bis[bis(thiourea-*S*)tellurium(II)] cations. *Inorganic Chemistry*.

[230] Foss O, Hauge S (1965). *Acta Chemica Scandinavica*.

[231] Beno MA, Sundell R, Williams JM (1984). *Croatica Chemica Acta*.

[232] Kumar V, Aravamudan G, Seshasayee M (1990). Interaction of heterocyclic thioamides with tellurium(II) and tellurium(IV). Syntheses and crystal structures of bis[2-(2-thioxo-1,3- thiazolidin-3-yl)-4,5-dihydro-1,3- thiazolium]hexabromotellurate(IV) and tris(1- methylimidazole-2-(3*H*)-thione)tellurium(II) bromide. *Polyhedron*.

[233] Asahara M, Tanaka M, Erabi T, Wada M (2000). Bis(2,6-dimethoxyphenyl)telluriium dihalides (Cl, Br or I) and dithiocyanate: crystal structure and temperature-dependent NMR spectra. *Journal of the Chemical Society, Dalton Transactions*.

[234] Drutkowski U, Strauch P (2001). Bis(1,2-dithiosquarato)tellurate(II) a new chalcogenochalcogenate. *Inorganic Chemistry Communications*.

[235] Drake JE, Khasrou LN, Mislankar AG, Ratnani R (1994). Synthesis and characterization of *N*,*N*-diethyl, pyrrolidyl, and piperidyl monothiocarbamate derivatives of dimethyltellurium(IV). Crystal structures of Me_2_
Te[SCONEt_2_]_2_, Me_2_
TeCI[SCONEt_2_], Me_2_
TeBr[SCONEt_2_], and Me_2_
TeI[SCONEt_2_]. *Inorganic Chemistry*.

[236] Bailey JHE, Drake JE, Khasrou LN, Yang J (1995). Synthesis and spectroscopic characterization of O-alkyl dithiocarbonate (Xanthate) derivatives of dimethyl- and diphenyltellurium(IV). Crystal structures of 
Me_2_
Te[S_2_COEt]_2_
and Ph_2_
Te[S_2_COEt]_2_. *Inorganic Chemistry*.

[237] Silvestru A, Haiduc I, Breunig HJ, Ebert KH (1995). Diphenyltellurium(IV) bis(diorganophosphinodithioates). X-ray crystal structure of Ph_2_Te(S_2_
PPh_2_)_2_ ⋅ 0.5CHCl_3_ and and a multinuclear NMR study of the decomposition process of Ph_2_
Te(S_2_PR_2_)_2_
to Ph_2_
Te^II^ and [R_2_
P(S)S]_2_. *Polyhedron*.

[238] Chung D-Y, Huang S-P, Kim K-W, Kanatzidis MG (1995). Discrete complexes incorporating heteropolychalcogenide ligands: ring and cage structures in [Au_2_(TeS_3_)_2_]^2−^
, [Ag_2_Te(TeS_3_)_2_]^2
−^, and [Ag_2_
Te(TeSe_3_
)_2_]^2−^. *Inorganic Chemistry*.

[239] Barnard PWC, Donaldson JD, Grimsey RMA, Dennes G, Russo U, Calogero S (1981). A study of square-planar tellurium(II) complexes with thiourea type ligands. *Inorganica Chimica Acta*.

[240] Foss O, Maartmann-Moe K (1986). *Acta Chemica Scandinavica, Series A*.

[241] Silvestru A, Haiduc I, Ebert KH, Breunig HJ (1994). Novel coordination pattern of dithiophosphorus ligands. Crystal and molecular structure of (diphenylphosphinodithioato)phenyltellurium(II), PhTeS_2_PPh_2_. Supramolecular association through monodentate biconnective dithiophosphorus ligands. *Inorganic Chemistry*.

[242] Silvestru A, Haiduc I, Ebert KH, Breunig HJ, Sowerby DB (1994). New aryltellurium(II) diorganophosphinodithioates. Crystal structure of red (294 K) and yellow (173 K) ^∞1^[PhTeS(S)PPh_2_] a supramolecular polymer displaying an unusual coordination pattern of the phosphinodithioato ligand. *Journal of Organometallic Chemistry*.

[243] Fleischer H, Stauf S, Schollmeyer D (1999). Experimental investigations and ab initio studies of tellurium(II) dithiolates, Te(SR)_2_. *Inorganic Chemistry*.

[244] Fleischer H, Schollmeyer D (2002). Conformation versus coordination: synthesis and structural investigations of tellurium(II) dithiolates derived from *β*-donor-substituted thiols. *Inorganic Chemistry*.

[245] Edelmann FT, Fischer A, Haiduc I (2003). Two structurally differing (heterogeometric) mesityltellurium(II) phosphor-1,1-dithiolates: the first monomeric dicoordinate MesTeS(S)PPh_2_ and a self-assembled tricoordinate [MesTeS(S)P(OPr^i^)_2_]_x_. *Inorganic Chemistry Communications*.

[246] Fleischer H, Mitzel NW, Schollmeyer D (2003). Tellurium(II) dialkanethiolates: n_p_(S)-*σ**(Te−S′) orbital interactions determine the ^125^
Te NMR chemical shift, and the molecular and crystal structure. *European Journal of Inorganic Chemistry*.

[247] Husebye S, Maartmann-Moe K, Mikalsen O (1989). *Acta Chemica Scandinavica*.

[248] Husebye S, Maartmann-Moe K, Mikalsen O (1989). *Acta Chemica Scandinavica*.

[249] Husebye S (1966). *Acta Chemica Scandinavica*.

[250] Refaat LS, Maartmann-Moe K, Husebye S (1984). *Acta Chemica Scandinavica, Series A*.

[251] Allen C, Boeyens JCA, Briggs AG (1987). Reaction of 3,4-dimethyl-1-oxa-6,6a*λ*
^4^-diselena-2-azapentalene with tetraphosphorus decasulphide: a new molecular rearrangement. X-Ray crystal structures of 3,4-dimethyl-1-oxa-6,6a*λ*
^4^-diselena-2-azapentalene, 2,4-dimethyl-1-thia-6,6a*λ*
^4^-diselena-3-azapentalene, and 2,4-dimethyl-1,6-dithia-6a*λ*
^4^-selena-3-azapentalene. *Journal of the Chemical Society, Chemical Communications*.

[252] Billing DG, Levendis DC, Reid DH, Rose BG (1995). 3,5-diphenyl-2-(2-pyridylamino)-1-thia-6,6a*λ*
^4^-diselenapentalene. *Acta Crystallographica, Section C*.

[253] Fedin VP, Sokolov MN, Geras'ko OA, Virovets AV, Podberezskaya NV, Fedorov VYe (1991). Triangular M_3_
Se_7_ and M_3_Se_4_ complexes (M = Mo, W). An X-ray study of Mo_3_
Se_7_(Et_2_
NCS_2_)_4_ and W_3_
Se_7_(Et_2_NCS_2_)_4_. *Inorganica Chimica Acta*.

[254] Meinwald J, Dauplaise D, Clardy J (1977). Peri-bridged naphthalenes. 2. Unsymmetrical diatomic chalcogen bridges. *Journal of the American Chemical Society*.

[255] Fedin VP, Sokolov MN, Virovets AV, Podberezskaya NV, Federov VYe (1992). Synthesis and crystal structure of [Mo_3_(*μ* − S)(*μ*−SSe)_3_(dtc)_3_]SeCN. An example of formation of unusual polymeric chains by cation and anion chalcogen atoms. *Polyhedron*.

[256] Fedin VP, Mironov YuV, Sokolov MN, Virovets AV, Podberezskaya NV, Federov VE (1992). *Russian Journal of Inorganic Chemistry*.

[257] Refaat LS, Maartmann-Moe K, Husebye S (1984). *Acta Chemica Scandinavica, Series A*.

[258] Bergholdt AB, Kobayashi K, Horn E (1998). Crystal structures and *ab initio* calculations of new dicationic telluranes (*λ*
^4^-tellane), 
[10-Te-4(C_2_
X_2_)]^2+^
(X = S, Se): positively charged hypervalent bonding systems. *Journal of the American Chemical Society*.

[259] Husebye S, Maartmann-Moe K (1983). *Acta Chemica Scandinavica, Series A*.

[260] Maaninen A, Chivers T, Parvez M, Pietikäinen J, Laitinen RS (1999). Syntheses of THF solutions of SeX_2_ (X = Cl, Br) and a new route to selenium sulfides Se_*n*_S_8−n_
(*n* = 
1−5): X-ray crystal structures of SeCl_2_(tht)_2_ and SeCl_2_
⋅ tmtu. *Inorganic Chemistry*.

[261] Sowrirajan S, Aravamudan G, Seshasayee M, Rout GC (1985). Synthesis and structure of a square-planar complex of selenium(II), tetrakis [*N*,*N*
′-(*o*-phenylene)thiourea]selenium(II) dichloride dihydrochloride, Se(C_7_H_6_
N_2_S)_4_
⋅ 2HCl. *Acta Crystallographica, Section C*.

[262] Chidambaram Sp, Aravamudan G, Rout GC, Seshasayee M (1986). Crystal structure of tris(*o*-phenylenethiourea)selenium(II) bromide pentahydrate, C_21_
H_18_
N_6_S_3_
Br_2_
Se ⋅ 5H_2_O. *Canadian Journal of Chemistry*.

[263] Chidambaram Sp, Aravamudan G, Seshasayee M (1989). Structure of bis[*μ*−*N*,*N*′-(*o*-phenylene)thiourea-*S*]-bis{bis[*N*,*N*′-(*o*-phenylene)thiourea-*S*]selenium(II)} perchlorate hexahydrate. *Acta Crystallographica, Section C*.

[264] Hordvik A, Julshamn K (1971). *Acta Chemica Scandinavica*.

[265] Llaguno EC, Paul IC (1976). Structure of a 3-aminothiathiophthen : *X*-ray analysis of 3-amino-2-methylthio-5-phenyl-6a-thiathiophthen {3-amino-2-methylthio-5-phenyl [1,2]dithiolo[1,5-*b*][1,2]dithiole-7-*S*
^*IV*^}. *Journal of the Chemical Society, Perkin Transactions 2*.

[266] Hordvik A, Oftedal P (1981). *Acta Chemica Scandinavica, Series A*.

[267] Hansen LK (1982). *Acta Chemica Scandinavica, Series A*.

[268] Reinke H, Kleist M (1999). *Private Communication*.

[269] Yokoyama M, Shiraishi T, Hatanaka H, Ogata K (1985). Synthesis and structure determination of [1,2]dithiolo[1,5-*b*][1,2,4]dithiazole-4-*S*
*^IV^* derivatives. *Journal of the Chemical Society, Chemical Communications*.

[270] Hordvik A, Saethre LJ (1972). *Acta Chemica Scandinavica*.

[271] Hordvik A (1971). *Acta Chemica Scandinavica*.

[272] Birknes B, Hordvik A, Saethre LJ (1982). *Acta Chemica Scandinavica, Series A*.

[273] Birknes B, Hordvik A, Saethre LJ (1975). *Acta Chemica Scandinavica, Series A*.

[274] Johnson PL, Llaguno EC, Paul IC (1976). A symmetrically substituted thiathiophthen with unequal sulphur-sulphur bond lengths: crystal and molecular structure of 3,4-diphenyl-6a-thiathiophthen {3,4-diphenyl[1,2]dithiolo[1,5-*b*][1,2]dithiole-7-*S*
*^IV^*}. *Journal of the Chemical Society, Perkin Transactions 2*.

[275] Meienberger MD, Hegetschweiler K, Rüegger H, Gramlich V (1993). The reactivity of complexes containing the [Mo_3_(*μ*
_3_
S)(*μ*S_2_)_3_]^4+^ core. Ligand substitution, sulfur elimination and sulfide binding. *Inorganica Chimica Acta*.

[276] Stelander B, Viehe HG, van Meerssche M, Germain G, Declercq JP (1977). *Bulletin des Societes Chimique Belges*.

[277] Wang Yu, Chen MJ, Wu CH (1988). Deformation density study of 2,4-diphenyl-6a-thiathiophthene. *Acta Crystallographica, Section B*.

[278] Hordvik A, Sletten E, Sletten J (1969). *Acta Chemica Scandinavica*.

[279] Wang Yu, Yeh SK, Wu SY, Pai CT, Lee CR, Lin KJ (1991). Deformation-density studies of thiathiophthenes. II. 2,4-diphenyl-6a-thiathiophthene. *Acta Crystallographica, Section B*.

[280] Hordvik A, Julshamn K (1971). *Acta Chemica Scandinavica*.

[281] Saethre LJ, Hordvik A (1975). *Acta Chemica Scandinavica, Series A*.

[282] Wang Yu, Wu SY, Cheng AC (1990). Deformation-density studies of thiathiophthenes. I. 2,5-dimethyl-6a-thiathiophthene. *Acta Crystallographica, Section B*.

[283] Sletten J (1974). *Acta Chemica Scandinavica, Series A*.

[284] Hordvik A, Milje LM (1973). *Acta Chemica Scandinavica*.

[285] Busetti V, Valle G, Piazzesi AM (1978). *Crystal Structure Communications*.

[286] Graubaum H, Tittelbach F, Lutze G (1997). Novel crown ethers with a trithiadiazapentalene-trithiotriuret redox system. *Angewandte Chemie International Edition*.

[287] Hansen LK, Hordvik A (1973). *Acta Chemica Scandinavica*.

[288] Iwasaki F, Manabe N, Nishiyama H (1997). Crystal and molecular structures of hypervalent thia/selena-pentalenes. *Bulletin of the Chemical Society of Japan*.

[289] Hordvik A (1971). *Acta Chemica Scandinavica*.

[290] Johnson SM, Newton MG, Paul IC (1969). Crystal and molecular structure of an unsymmetrical 6a-thiathiophthen: single-crystal *X*-ray analysis of 3-benzoyl-5-*p*-bromophenyl-2-methylthio-6a-thiathiophthen. *Journal of the Chemical Society B*.

[291] Gloe K, Graubaum H, Wüst M, Rambusch T, Seichter W (2001). Macrocyclic and open-chain ligands with the redox switchable trithiadiazapentalene unit: synthesis, structures and complexation phenomena. *Coordination Chemistry Reviews*.

[292] Sotiropoulos J-M, Lamazouere A-M, el Batouti N, Sotiropoulos J, Dahan F, Jaud J (1990). *Phosphorus, Sulfur and Silicon and the Related Elements*.

[293] Fabian J, Gloe K, Wüst M, Krüger-Rambusch T, Rademacher O, Graubaum H (1998). The structure of 3,4-diaza-1,6,6a*λ*
^4^-trithiapentalenes- a combined experimental and theoretical study. *Phosphorus, Sulfur and Silicon and Related Elements*.

[294] Yih K-H, Lin Y-C, Lee G-H, Wang Yu (1995). Synthesis and crystal structure of the first 6a-thiathiophthen metal complex [Mo(CO)_5_PPh_2_]_2_(*μ*−C_5_H_2_S_3_). *Journal of the Chemical Society, Chemical Communications*.

[295] Meyer B, Wunderlich H (1982). *Zeitschrift für Naturforschung, Section B*.

[296] Llusar R, Uriel S, Vicent C (2004). Single-component magnetic conductors based on Mo_3_S_7_ trinuclear clusters with outer dithiolate ligands. *Journal of the American Chemical Society*.

[297] Zeying Z, Youqi T, Chenggang D, Zhuang T (1985). *Science in China, Series B*.

[298] Coucouvanis D, Hadjikyriacou A (1987). Synthesis and structural characterization of the Et_4_
N^+^ salts of the new [[(S_2_)_2_
MoO]_2_S]^2−^
and [[(S_2_)_2_
MoO]_2_S]_2_
^2−^ oxo-disulfido-molybdate(VI) anions. *Inorganic Chemistry*.

[299] Hadjikyriacou AI, Coucouvanis D (1989). Synthesis, structural characterization, and properties of the [Mo_2_
O_2_S_9_]^2−^
thio anion and the [Mo_4_
O_4_
S_18_]^2−^
, [Mo_2_O_2_
S_8_(SCH_3_)]^−^, and [Mo_2_
O_2_
S_8_Cl]^−^ derivatives. *Inorganic Chemistry*.

[300] Bottcher P, Buchkremer-Hermanns H (1987). *Zeitschrift für Naturforschung, Section B*.

[301] Closs F, Srdanov G, Wudl F (1989). 3-Oxo-4-thioxo-1,2,5,6-tetrathiapentalene (OTTP): a novel thiocarbon with an unusual chalcogen network in its solid state structure. *Journal of the Chemical Society, Chemical Communications *.

[302] Yang X, Rauchfuss TB, Wilson S (1990). The chemistry of C_6_
S_10_: a channel structure for C_6_
S_10_(CS_2_)_0.5_ and access to the versatile DMAD ⋅C_3_
S_4_
O (DMAD = dimethylacetylenedicarboxylate). *Journal of the Chemical Society, Chemical Communications *.

[303] Simonnet-Jegat C, Jourdan N, Robert F, Bois C, Sécheresse F (1994). Evidence of the **W**(**O**)(S_2_)_2_ core as an intermediate in the acidification of WS_4_
^2^
^−^
. Structural characterization of W(O)(S_2_)_2_(bpy) and W(O)(S_2_)_2_(phen). *Inorganica Chimica Acta*.

[304] Clegg W, Mohan N, Müller A, Neumann A, Rittner W, Sheldrick GM (1980). Crystal and molecular structure of [N(CH_3_)_4_]_2_[Mo_2_O_2_
S_2_(S_2_)_2_]: a compound with two S_2_
^2^
^−^ ligands. *Inorganic Chemistry*.

[305] Shibahara T, Iwai N, Sasaki M, Sakane G (1997). Photochromism of dinuclear molybdenum complexes with disulfur and ethylene-1,2-dithiolate ligands. *Chemistry Letters*.

[306] Zhu H-P, Chen C-N, Liu Q-T, Chen J-T (1998). A triangular [Mo_3_
S_7_]^4+^
complex: tris-(diethyldithiocarbamato-*S*,*S*′)tris(*μ*
_2_−*η*
^2^-disulfido)(*μ*
_3_-sulfido)trimolybdenum(IV)-(3
*M*
*o*−*M*
*o*) diethyldithiocarbamate. *Acta Crystallographica, Section C*.

[307] Yu R-M, Lu S-F, Huang X-Y, Wu Q-J, Huang J-Q (1998). A new ionic trimolybdenum cluster compound: Mo_3_
S_7_(S_2_
P(ipro)_2_)_3_[S_2_
P(ipro)_2_]. *Chinese Journal of Structural Chemistry*.

[308] Bensch W, Schur M (1997). Crystal structure of bis(tetramethylammonium) trismolybdenumtridecasulfide, [N(CH_3_)_4_]_2_Mo_3_
S_13_. *Zeitschrift für Kristallographie: New Crystal Structures*.

[309] Lu S, Ke Y, Li J, Zhang Y (2002). Synthesis, structure and characterizations of a molybdenum sulfide complex with inorganic cluster [Mo_3_
S_13_]^2−^
and organic amine Me_4_N
^+^. *Crystal Research and Technology*.

[310] Ellermeier J, Bensch W (2001). Solvothermal syntheses, crystal structures and properties of thiomolybdates with complex transition metal cations. *Zeitschrift für Naturforschung, Section B*.

[311] Yang Yu, Liu Q, Wu D (1993). The first triangular V_3_S_7_
^2^
^+^ complex. Synthesis and structure of (Et_4_N)[V_3_S_7_(Me_2_dtc)_3_]⋅CH_3_CN. *Inorganica Chimica Acta*.

[312] Akasaka T, Nakano M, Tamura H, Matsubayashi G-E (2002). Preparation and properties of tin(IV) complexes with the sulfur-rich dithiolate C_3_S_5_ and C_8_H_4_S_8_ ligands and their oxidation. *Bulletin of the Chemical Society of Japan*.

[313] Lu S-F, Wu Q-J, Chen H-B, Yu R-M, Huang J-Q (1994). *Chinese Journal of Structural Chemistry*.

[314] Ellermeier J, Bensch W (2002). Solvothermal synthesis, crystal structure and properties of Mn_2_(tren)_3_[Mo_2_OS_6_]_2_⋅1.3H_2_O exhibiting the new polymeric [Mn_2_(tren)_3_]_*n*_
^4^
^+^ chain. *Transition Metal Chemistry*.

[315] Dereu NLM, Zingaro RA, Meyers EA (1981). *Crystal Structure Communications*.

[316] Bollinger JC, Ibers JA (1995). Reactions of [M(Se_4_)_2_]^2−^ anions with TePEt_3_: ^77^Se and ^125^Te spectra of [MTe_*n*_Se_8_
_−_
_*n*_]^2−^ (M = Zn, Cd, Hg; = 0−4) and preparation and crystal structure of [PPh_4_]_2_[Hg(Te_2_Se_2_)_2_]. *Inorganic Chemistry*.

[317] Foss O, Maartmann-Moe K (1987). *Acta Chemica Scandinavica, Series A*.

[318] Foss O, Maartmann-Moe K (1987). *Acta Chemica Scandinavica, Series A*.

[319] Sekar P, Ibers JA (2004). Syntheses and characterization of some mixed Te/Se polychalcogenide anions [Te_*m*_Se_*n*_]^2−^. *Inorganic Chemistry*.

[320] Hummel H-U, Fischer T, Wolski A (1994). Strukturen neuer Se^II^ und Te^II^-Komplexe mit 2,2-Dicyanethylen-1,1-dithiolat, 2,2-Dicyanethylen-1,1-thioselenolat und 2,2-Dicyanethylen-1,1-diselenolat. *Zeitschrift für anorganische und allgemeine Chemie*.

[321] Hauge S, Tysseland M (1971). *Acta Chemica Scandinavica*.

[322] Pathirana HMKK, Zingaro RA, Meyers EA, Reibenspies JH, Dufner DC (1990). Substituted selenourea-tellurium tetrahalide adducts: precursors to selenium-tellurium alloys. *Heteroatom Chemistry*.

[323] Zagler R, Eisenmann B (1991). *Zeitschrift für Naturforschung, Section B*.

[324] Kanatzidis MG, Huang S-P (1989). Unanticipated redox transformations in gold polyselenides. Isolation and characterization of [Au_2_
Se_2_(Se_4_)_2_]^2−^ and [Se_11_]^2−^. *Inorganic Chemistry*.

[325] Krebs B, Luhrs E, Willmer R, Ahlers F-P (1991). Chloro- und polyselenoselenate(II) darstellung, struktur und eigenschaften von [Ph_3_(C_2_
H_4_OH)P]_2_[SeCl_4_] ⋅ MeCN, [Ph_4_P]_2_[Se_2_Cl_6_] und [Ph_4_P]_2_[Se(Se_5_)_2_]. *Zeitschrift für anorganische und allgemeine Chemie*.

[326] Cea-Olivares R, Canseco-Melchor G, García-Montalvo V, Hernández-Ortega S, Novosad J (1998). Synthesis of the first neutral spiro selenium(II) complex containing a true square planar Se(Se_4_) core—preparation and crystal structure of Bis[*N*-(diphenylphosphanylselenoyl)-*P*,*P*-diphenylphosphanylselenoic amidato-*Se*, *Se*′]selenium(II). *European Journal of Inorganic Chemistry*.

[327] Billing DG, Ferg EE, Lai L-L, Levendis DC, Reid DH (1993). Structures of 2,3-diethyl-6,7-dihydro-5*H*-2a*λ*
^4^-selena-2,3,4a,7a-tetraazacyclopent[*c*
*d*]indene-1(2*H*),4(3*H*)-diselone and 1,4-bis(ethylimino)-5,6-dihydro-2,2a*λ*
^4^,3-triselena-4a,6a-diazacyclopenta[*c*
*d*]pentalene. *Acta Crystallographica, Section C*.

[328] Richter R, Sieler J, Hansen LK, Kohler R, Beyer L, Hoyer E (1991). *Acta Chemica Scandinavica*.

[329] Batchelor RJ, Einstein FWB, Gay ID, Gu J-H, Pinto BM, Zhou X-M (1990). Electron-transfer reaction of a selenium coronand-copper(II) complex. Formation of the stable 1,5,9,13-tetraselenacyclohexadecane dication. *Journal of the American Chemical Society*.

[330] Batchelor RJ, Einstein FWB, Gay ID, Gu J-H, Pinto BM, Zhou X-M (2000). Redox chemistry of the selenium coronand, 1,5,9,13-tetraselenacyclohexadecane, and a mechanistic study of the electron transfer reaction of its Cu(II) complex. *Canadian Journal of Chemistry*.

[331] Hummel H-U, Fischer E, Fischer T, Gruss D, Franke A, Dietzsch W (1992). *Chemische Berichte*.

[332] Bhattacharyya P, Slawin AMZ, Woollins JD (2000). Reaction of [{PhP(Se)(*μ*−Se)}_2_] with dialkyl cyanamides: X-ray crystal structures of the phosphorus-containing triselenapentalenes [Me_2_N-C(Se)=N]_2_P(Se)Ph and [O(CH_2_CH_2_)_2_N-C(Se)=N]_2_P(Se)Ph. *Angewandte Chemie International Edition*.

[333] Nakahodo T, Takahashi O, Horn E, Furukawa N (1997). First molecular and electronic structure determination of the dicationic salt of 1,11-(methanoselenomethano)-5H,7H-dibenzo[b,g] [1.5]diselenocin by X-ray crystallographic analysis and ab initio calculation. *Chemical Communications*.

[334] Evans WJ, Rabe GW, Ansari MA, Ziller JW (1994). Polynuclear lanthanide complexes: formation of a selenium-centered Sm_6_ complex, [{(C_5_Me_5_)Sm}_6_Se_11_]. *Angewandte Chemie International Edition*.

[335] Almond MJ, Drew MGB, Redman H, Rice DA (2000). A new simple synthetic route to M_3_Se_7_ (M = Mo or W) core containing complexes: crystal structure and characterisation of [M_3_(*μ*
_3_−Se)(*μ*−Se_2_)_3_(dtc)_3_]_2_Se. *Polyhedron*.

[336] Kornienko AY, Emge TJ, Brennan JG (2001). Chalcogen-rich lanthanide clusters: cluster reactivity and the influence of ancillary ligands on structure. *Journal of the American Chemical Society*.

[337] Hauge S, Opedal D, Arskog J (1975). *Acta Chemica Scandinavica, Series A*.

[338] Hordvik A, Julshamn K (1971). *Acta Chemica Scandinavica*.

[339] Hauge S (1979). *Acta Chemica Scandinavica, Series A*.

[340] Dietz J, Müller U, Müller V, Dehnicke K (1991). *Zeitschrift für Naturforschung, Section B*.

[341] Hordvik A, Porten JA (1973). *Acta Chemica Scandinavica*.

[342] Adamo C, Demartin F, Deplano P (1996). Electrochemical synthesis of tetrakis[*N*-methylbenzothiazole-2(3*H*)-selone]selenium(2
+) tetrafluoroborate: an uncommon dication containing the mixed-valence Se_5_ framework. *Chemical Communications*.

[343] Hillier AC, Liu S-Y, Sella A, Elsegood MRJ (1999). (PhTe)_3_
^−^: the anionic tellurium analogue of I_3_
^−^. *Angewandte Chemie International Edition*.

[344] Dhingra SS, Haushalter RC (1994). A novel ternary zintl anion: synthesis and structural characterization of the [Cu_4_
SbTe_12_]^3−^
anion. *Journal of the American Chemical Society*.

[345] Warren CJ, Haushalter RC, Bocarsly AB (1994). Electrochemical synthesis and structural characterization of the one-dimensional chain compound (Et_4_
N)_2_[As_2_Te_5_] and the tellurido arsenate (Me_4_N)_4_[As_4_Te_6_] ⋅ 2en. *Chemistry of Materials*.

[346] Schreiner B, Dehnicke K, Maczek K, Fenske D (1993). [K(15-Krone-5)_2_]_2_Te_8_—ein bicyclisches polytellurid. *Zeitschrift für anorganische und allgemeine Chemie*.

[347] Dhingra SS, Haushalter RC (1994). Synthesis and structure of the new gold polytelluride anion [Au_2_Te_12_]^4−^. *Inorganic Chemistry*.

[348] Jin G-X, Arikawa Y, Tatsumi K (2001). Spontaneous formation of a diamond-crown structure of Re_8_ polyselenide and a cage structure of Re_3_ polytelluride [2]. *Journal of the American Chemical Society*.

[349] Chen X, Huang X, Li J (2001). Rb_4_Hg_5_(Te_2_)_2_(Te_3_)_2_Te_3_,[Zn(en)_3_]_4_In_16_(Te_2_)_4_(Te_3_)Te_22_, and K_2_Cu_2_(Te_2_)(Te_3_): novel metal polytellurides with unusual metal-tellurium coordination. *Inorganic Chemistry*.

[350] McConnachie JM, Ansari MA, Bollinger JC, Salm RJ, Ibers JA (1993). Synthesis and structural characterization of the telluroargentate [PPh_4_]_2_[NEt_4_][AgTe_7_] and telluromercurate [PPh_4_]_2_[HgTe_7_] compounds containing the unprecedented *η*
^3^ − Te_7_
^4−^ polytelluride anion. *Inorganic Chemistry*.

[351] Smith DM, Roof LC, Ansari MA (1996). Synthesis, reactivity, and structural characterization of the nonclassical [MTe_7_]^*n−*^ anions (M = Ag,Au,*n* = 3;M = Hg,*n* = 2 ). *Inorganic Chemistry*.

[352] Ansari MA, Bollinger JC, Ibers JA (1993). Synthesis and structural characterization of the [AuTe_7_]^3−^
anion: a planar species with an unprecedented coordination mode. *Journal of the American Chemical Society*.

[353] Sekar P, Arnold FP, Ibers JA (2002). Synthesis, structure, and theoretical study of the nonclassical [CuTe_7_]^3−^
anion. *Inorganic Chemistry*.

[354] Freedman D, Emge TJ, Brennan JG (2002). Chalcogen-rich lanthanide clusters: compounds with Te^2−^,(TeTe)^2−^,TePh,TeTePh,(TeTeTe(Ph)TeTe)^5−^, and [(TeTe)_4_TePh]^9−^ ligands; single source precursors to solid-state lanthanide tellurides. *Inorganic Chemistry*.

[355] Klinkhammer KW, Bottcher P (1990). *Zeitschrift für Naturforschung, Section B*.

[356] Witthaut D, Kirschbaum K, Conrad O, Giolando DM (2000). Isolation of a catenated organotelluride anion in the sodium borohydride reduction of diphenylditelluride. *Organometallics*.

[357] Fujihara H, Nakahodo T, Furukawa N (1996). Preparation and crystal structure of a new tetracoordinated cyclic selenurane with two unsymmetrical apical ligands of oxygen and selenium: transannular hypercoordination between oxy- and diseleno-groups. *Chemical Communications*.

[358] Fujihara H, Nakahodo T, Furukawa N (1995). Synthesis of 5*H*,7*H*-dibenzo[*b*,*g*][1,5]selenoxocine from a selenonium salt of 5*H*,7*H*-dibenzo[*b*,*g*][1,5]diselenocine and first X-ray evidence for the transannular oxygen-selenium interaction. *Tetrahedron Letters*.

[359] Sheldrick WS, Devillanova FA (2006). Polychalcogenides. *Handbook of Chalcogen Chemistry*.

[360] Beck J, Hormel A, Koch M (2002). 1,2-Dichalcogenolylium ions (C_3_Cl_3_
E_3_)^+^ from equilibria involving dichalcogen dichlorides E_2_
Cl_2_(E=
S,Se,Te). *European Journal of Inorganic Chemistry*.

[361] Liaw W-F, Lai C-H, Chiou S-J (1995). Synthesis and characterization of polymeric Ag(I)-telluroether and Cu(I)-diorganyl ditelluride complexes: crystal structures of [Ag(MeTe(CH_2_
)_3_TeMe)_2_]_*n*_[BF_4_]_*n*_,[(*μ*
_2−_
MeTeTeMe)Cu(*μ* − Cl)]*_n_*, and [Ag_2_(NCCH_3_)_4_(*μ*
_2_
−(*p* − C_6_H_4_
F)TeTe(*p* − 
C_6_
H_4_F))_2_][BF_4_]_2_. *Inorganic Chemistry*.

[362] Eveland JR, Whitmire KH (1996). Synthesis and characterization of the novel iron carbonyl tellurium chloride cluster [Fe_2_
(CO)_6_(*μ*-
Cl)(*μ*-
TeCl)_2_]_2_[*η*
^2^,*μ*
_2_,*μ*
_2_
-
Te_2_
Cl_10_], and its decomposition to the zintl ion complex [Fe_2_
(CO)_6_(*η*
^2^,*μ*
_2_,*μ*
_2_
^-^Te_4_)(*μ−*TeCl_2_)]. *Angewandte Chemie International Edition*.

[363] Hauge S, Vikane O (1985). *Acta Chemica Scandinavica, Series A*.

[364] Foss O, Hermansen R, Maroy K, Moberg T (1987). *Acta Chemica Scandinavica, Series A*.

[365] Hauge S, Vikane O (1988). *Acta Chemica Scandinavica, Series A*.

[366] Husebye S, Thowsen AG (1981). *Acta Chemica Scandinavica, Series A*.

[367] Foss O, Husebye S (1966). *Acta Chemica Scandinavica*.

[368] von Deuten K, Schnabel W, Klar G (1979). *Crystal Structure Communications*.

[369] Appa Rao GVN, Seshasayee M, Aravamudan G, Radha K (1983). Crystal and molecular structure of chlorotris[bis(2-hydroxyethyl)dithiocarbamato]tellurium(IV) dihydrate. *Inorganic Chemistry*.

[370] Vikane O (1975). *Acta Chemica Scandinavica, Series A*.

[371] von Deuten K, Schnabel W, Klar G (1980). *Phosphorus and Sulfur*.

[372] Drake JE, Khasrou LN, Mislankar AG, Ratnani R (1999). Synthesis, spectroscopic studies, and structural studies of *O*,*O*-alkylene dithiophosphate and *N*,*N*-dimethyl and diethyl dithiocarbamate derivatives of halodimethyltellurium(IV). *Inorganic Chemistry*.

[373] Eide J, Foss O, Maartmann-Moe K, Maberg O, Scheie A (1987). *Acta Chemica Scandinavica, Series A*.

[374] Bergman J, Laitalainen T, Sundberg MR, Uggla R, Kivekäs R (1998). Stereospecific synthesis and crystal structure of the racemate of 1-thia-2-tellura-1(1-allyl-4-chloro)cyclopentane 2,2,2-trichloride. *Polyhedron*.

[375] Cox MJ, Tiekink ERT (1999). Crystal structure of chlorotris(dimethyldithiocarbamato)tellurium(IV), [Te(S_2_CNMe_2_)_3_
Cl]. *Zeitschrift für Kristallographie: New Crystal Structures*.

[376] Sundberg MR, Laitalainen T, Bergman J, Uggla R, Matikainen J, Kaltia S (1998). Plasticity of Cl-Te-Cl fragments. Synthesis, single-crystal X-ray, and NBO study of (1-thia-2-tellura-1-phenyl-4-chloro)cyclopentane 2,2,2-trichloride. *Inorganic Chemistry*.

[377] Foss O, Johnsen K, Maartmann-Moe K, Maroy K (1966). *Acta Chemica Scandinavica*.

[378] Drake JE, Drake RJ, Khasrou LN, Ratnani R (1996). Synthesis and spectroscopic characterization of halodimethyl(*O*-alkyl dithiocarbonato)tellurium(IV) compounds. Crystal structures of Me_2_TeCl[S_2_COEt] and Me_2_
TeI[S_2_CO(*i*−Pr)]. *Inorganic Chemistry*.

[379] Fredin KS, Maroy K, Slogvik S (1975). *Acta Chemica Scandinavica, Series A*.

[380] Alvarado-Rodríguez JG, García Gutiérrez MC, Cea-Olivares R (2000). Aspectos geométricos de fenocalcogenotelurinas. Estudio de la estructura molecular y cristalina de O(C_6_
H_4_)_2_Te(Cl)S_2_
CN(CH_2_
CH)_2_. *Revista Mexicana de Fisica*.

[381] Carmalt CJ, Norman NC, Farrugia LJ (1995). Octahedral coordination complexes of tellurium tetrachloride. *Polyhedron*.

[382] Haas A, Kasprowski J, Angermund K (1991). Synthese, Strukturen und Eigenschaften der Cyclothiaselenazenium-Kationen [Se_2_
N_2_S]_2_
^2^
^+^, [XSe_2_N_2_S]^+^, [Se_2_N_2_S]^2+^
, [S_3_SeN_5_]^+^ sowie Cl_2_Se_2_N_2_S und SeSN_2_ . TiCl_4_. *Chemische Berichte*.

[383] Hu J, Zhuang H-H, Liu S-X, Huang J-L (1998). Syntheses and structures of chalcogen-molybdenum 
clusters; [Mo_3_XSe_6_{S_2_P(OEt)_2_}_3_]Cl, [Mo_3_X(SeS)_3_{S_2_
P(OEt)_2_}_3_]I and [Mo_3_
XSe_3_{
S_2_P(OEt)_2_}_4_(py)](X=
0.65S + 0.35Se,py=
C_5_H_5_
N). *Transition Metal Chemistry*.

[384] Villa AC, Nardelli M, Tani MEV (1970). The crystal and molecular structure of *α*,*α*′-diselenobisformamidinium dichloride. *Acta Crystallographica, Section B*.

[385] Acampora LA, Elman BS, Sandman DJ (1989). Structural and magnetic studies of electrochemically crystallized halides of 1,4,5,8-tetraselenonaphthalene (TSeN). *Inorganic Chemistry*.

[386] Eriksen K, Hauge S, Marøy K (2001). Syntheses and crystal structures of di-*μ*-chloro-bis [dithiocyanatoselenate(II)] and di-*μ*-bromo-bis [dithiocyanatoselenate(II)] salts. *Phosphorus, Sulfur and Silicon and Related Elements*.

[387] Apblett A, Chivers T, Fait JF (1989). A simple synthesis of [NS]^+^[AlCl_4_
]^−^ and the insertion reaction with alkylselenium halides: X-ray structure of [N_2_S_2_
SeCl]^+^[AlCl_4_]^−^. *Chemical Communications*.

[388] Apblett A, Chivers T, Fait JF (1990). Preparation of thiazyl tetrachloroaluminate and trifluoromethanesulfonate and
reactions of the thiazyl cation with thiadiazoles and organoselenium halides: X-ray crystal
structure of [N_2_
S_2_
SeCl][AlCl_4_]. *Inorganic Chemistry*.

[389] Barclay TM, Cordes AW, Goddard JD (1997). Benzo-bridged bis(1,2,3-dithiazoles) and their selenium analogues. Preparation, molecular and electronic structures, and redox chemistry. *Journal of the American Chemical Society*.

[390] Shang M-Y, Huang J-L, Lu J-X (1984). The structure of *μ*
_3_-thio-*μ*
_3_-tris(disulfido)-chlorato-*cyclo*-tris[(diethyl dioxodithiophosphato-*S*,*S*′)molybdenum](3
*M*
*o* − *M*
*o*), C_12_
H_30_
ClMo_3_
O_6_P_3_
S_13_. *Acta Crystallographica, Section C*.

[391] Dingming W, Jianquan H, Yuhui L, Jinling H (1986). *Acta Physico-Chimica Sinica*.

[392] Klingelhöfer P, Müller U, Friebel C, Pebler J (1986). Thiochloroanionen von Molybdän(IV). Die Kristallstruktur von (NEt_4_)_3_[Mo_3_(*μ*
_3_−S)(*μ*−S_2_
)_3_Cl_6_]
Cl ⋅ CH_2_
Cl_2_ Kristallstruktur, EPR-Spektrum und magnetische Eigenschaften von (NEt_4_)_2_)
[Mo_2_(*μ*−S_2_
)(*μ*−Cl)_2_
Cl_6_]. *Zeitschrift für anorganische und allgemeine Chemie*.

[393] Zalkin A, Hopkins TE, Templeton DH (1966). The crystal structure of chlorothiodiazyl chloride, S_3_
N_2_Cl_2_. *Inorganic Chemistry*.

[394] Rabe S, Müller U (1999). Crystal structure of 4,5-dichloro-1,2,3-dithiazolium chloride, [C_2_
NS_2_Cl_2_]Cl. *Zeitschrift für Kristallographie: New Crystal Structures*.

[395] Rees CW, Sivadasan S, White AJP, Williams DJ (2002). Conversion of tetrazoles into hydrazonoyl chlorides. Novel donor-dithiazolium interactions. *Journal of the Chemical Society, Perkin Transactions 1*.

[396] Britten JF, Cordes AW, Haddon RC (2002). A 1,2,3,5-dithiadiazolyl dimeric radical cation. Preparation and solid state characterization of 1,3-[(S_2_
N_2_C)C_6_H_4_(CN_2_S_2_)]_2_[Cl]_3_. *CrystEngComm*.

[397] Fedin VP, Sokolov MN, Myakishev KG, Geras'ko OA, Fedorov VYe, Macicek J (1991). Mechanochemical synthesis of soluble complexes
containing M_3_
S_7_
^4^
^+^ and M_3_
Se_7_
^4^
^+^
fragments from polymeric M_3_
Y_7_Br_4_(M = 
Mo,W;Y = 
S,Se). The crystal structure of (PPN)_2_
W_3_S_7_Cl_6_. *Polyhedron*.

[398] Sokolov MN, Gushchin AL, Naumov DYu, Gerasko OA, Fedin VP (2005). Cluster oxalate complexes [M_3_(*μ*
_3_
− Q)(*μ*
_2_
− Q_2_)_3_(C_2_
O_4_)_3_]^2−^
and [Mo_3_(*μ*
_3_− Q)(*μ*
_2_− Q)_3_(C_2_
O_4_)_3_(H_2_
O)_3_]^2−^(M=Mo,W;Q=
S,Se): echanochemical synthesis and crystal structure. *Inorganic Chemistry*.

[399] Borgs G, Keck H, Kuchen W, Mootz D, Wiskemann R, Wunderlich H (1991). *Zeitschrift für Naturforschung, Section B*.

[400] Grundtvig F, Hordvik A (1971). *Acta Chemica Scandinavica*.

[401] Zhu H-P, Liu Q-T, Chen C-N, Deng Y-H (1998). Synthesis and structure of a [Mo_3_
S_7_]^3.5+^ complex 
[Mo_3_(*μ*
_3_
−
S)(*μ*
_2_
−S_2_)_3_(Et_2_
dtc)_3_]_2_
Cl. *Jiegou Huaxue*.

[402] Barclay TM, Beer L, Cordes AW (1999). Sterically protected 1,2,3-dithiazolyl radicals: preparation and structural characterization of 4-chloro-5-pentafluorophenyl-1,2,3-dithiazlyl. *Chemical Communications*.

[403] Xian-Ti L, Jia-Xi L, Jin-Ling H, Jian-Quan H (1990). *Chinese Journal of Structural Chemistry*.

[404] Ruangsuttinarupap S, Gross H-D, Willing W, Müller U, Dehnicke K (1986). 4-Methyl-1,2,3,5-dithiadiazoliumsalze Die Kristallstrukturen von (CH_3_
CN_2_S_2_
)_5_[CoCl_4_
]Cl_3_ und (CH_3_
CN_2_S_2_)Cl. *Zeitschrift für anorganische und allgemeine Chemie*.

[405] Virovets AV, Laege M, Krebs B (1996). Nonvalent interactions in the crystal structures of (Et_4_N)_2_[Mo_3_S_7_Br_6_] and (Et_4_
N)(H_9_O_4_)[Mo_3_
S_7_Cl_6_] clusters. *Journal of Structural Chemistry*.

[406] Geras'ko OA, Virovets AV, Dybtsev DN, Clegg W, Fedin VP (2000). Crystal structure of a supramolecular cluster adduct with cucurbituril, (H_3_
O)_4_[W_3_
S_7_Cl_6_]_2_(C_36_N_24_
O_12_
H_36_) ⋅ 8
H_2_O. *Koordinatsionnaya Khimiya*.

[407] Virovets AV, Volkov OV (2000). Specific nonbonding contacts in the crystal structure of a solid solution [Mo_3_
(*μ*
_3_
−S)(*μ*− S_2_)_3_
(S_2_
CNEt_2_)_3_
]Cl_0.53_Br_0.47_. *Journal of Structural Chemistry*.

[408] Virovets AV, Slovokhotov YuL, Struchkov YuT (1990). *Russian Journal of Coordination Chemistry*.

[409] Sellmann D, Hannakam M, Knoch F, Moll M (1993). Transition metal complexes with sulfur ligands—part XCIII. Synthesis, structure and
reactivity of [Mo^IV^(*μ*−S)
(^`bu^
S_4_,
)]_2_
(^`bu^S_4_,^2^
^−^ = 1.2-bis(di(t-butyl)-2-mercaptophenylthio)ethane(2
−)). *Inorganica Chimica Acta*.

[410] Beer L, Cordes AW, Haddon RC (2002). A *π*-stacked 1,2,3-dithiazolyl radical. Preparation and solid state characterization of (Cl_2_
C_3_NS)(ClC_2_
NS_2_). *Chemical Communications*.

[411] Pathirana HMKK, Reibenspies JH, Meyers EA, Zingaro RA (1991). Structure of di-*μ*-bromo-(tetrabromo-1_*k*_
^4^Br)bis(*N*,*N*-dimethylselenourea-2_*k*_
^2^Se)ditellurium(II,IV)-acetonitrile-methanol (2/3/1). *Acta Crystallographica, Section C*.

[412] Rudd MD, Lindeman SV, Husebye S (1996). Structural characteristics of three-coordinate arylhalide tellurium(II) complexes with chalcogen ligands. Synthesis, spectroscopic characterization and X-ray structural studies of bromo[N-methylbenzothiazole-2(3H)-selone]phenyltellurium(II), Bromophenyl[tris(dimethylamino)phosphaneselenide]-tellurium(II) and tris(dimethylamino)phosphanesulfide. *Acta Chemica Scandinavica*.

[413] Schnabel W, von Deuten K, Klar G (1981). *Crystal Structure Communications*.

[414] Herland P, Lundeland M, Maroy K (1976). *Acta Chemica Scandinavica, Series A*.

[415] Husebye S (1979). *Acta Chemica Scandinavica, Series A*.

[416] Vikane O (1975). *Acta Chemica Scandinavica, Series A*.

[417] Foss O, Maroy K (1986). *Acta Chemica Scandinavica, Series A*.

[418] Rudd MD, Lindeman SV, Husebye S (1997). Tautomeric conversion of a thiourea ligand upon formation of a hypervalent tellurium (II) complex. Synthesis, and X-ray structural studies of N-Phenyl-*N*′-(1,3-thiazol-2-yl)-thiourea and bromophenyl [1-phenyl-3-(1′,3′-thiazol-3′-ium-2′-yl)-isothioureidato]tellurium(II). *Phosphorus, Sulfur and Silicon and Related Elements*.

[419] Hauge S, Maroy K (1992). *Acta Chemica Scandinavica*.

[420] Horn E, Nakahodo T, Furukawa N (2000). Crystal structure of 1,6-dibromo-2-phenyl-1,2-diselenaacenaphthylene, BrSe(C_10_
H_5_Br)Se(C_6_
H_5_). *Zeitschrift für Kristallographie: New Crystal Structures*.

[421] Larsen S, Henriksen L (1984). *Acta Chemica Scandinavica, Series A*.

[422] Fedin VP, Sokolov MN, Geras'ko OA, Virovets AV, Podberezskaya NV, Fedorov VYe (1992). Synthesis and structure of a new selenium-bridged tungsten cluster, [W_3_
Se_7_(S_2_P(
OEt)_2_)_3_]Br. *Polyhedron*.

[423] Kienitz CO, Thöne C, Jones PG (2000). The coordination chemistry of 2,2′-dipyridyldiselenide (PySeSePy)—part 2. Complexes with manganese, copper and zinc. *Zeitschrift für Naturforschung, Section B*.

[424] Pathirana HMKK, Reibenspies JH, Meyers EA, Zingaro RA (1991). Structure of *N*
^1^,*N*
^1^
,*N*
^3^,*N*
^3^-tetramethyl-*α*,
*α*
^1^
-diselenobisformamidinium bromide. *Acta Crystallographica, Section C*.

[425] Béreau V, Ibers JA (2000). Synthesis and characterization by diffraction and ^31^P- and ^77^Se-NMR spectroscopy of [Mo_3_
(*μ*
_3_
−Se)(*μ*
_2_−Se_2_)_3_{N(SePPh_2_)_2_}_3_]Br and [Mo_3_(*μ*
_3_−Se)(*μ*
_2_−Se_2_)_3_{Se_2_
P(OCH_2_CH_3_)_2_
}_3_]Br. *Comptes Rendus de l'Academie des Sciences, Series IIC*.

[426] Hobert SE, Noll BC, DuBois MR (2001). Synthesis of a rhenium(V) polysulfide complex and a study of its reactivity with hydrogen. *Organometallics*.

[427] Fedin VP, Müller A, Filipek K (1994). Extrusion of molecular clusters from solid-state materials: synthesis by application of *γ*-irradiation. Molecular and crystal structure of (H_9_
O_4_)(Et_4_
N)[Mo_3_S_7_Br_6_]. *Inorganica Chimica Acta*.

[428] Béreau V, Pernin CG, Ibers JA (2000). Reactivity of the [Mo_3_(*μ*
_3_
−S)(*μ*
_2_
−S_2_)_3_
Br_6_]^2−^ anion toward the imidodiphosphinochalcogenido ligands [N(*Q*PPh_2_
)2]^−^[*Q*=S,Se]: synthesis and characterization of [Mo_3_
(*μ*
_3_−S)(*μ*
_2_
−S_2_)_3_{N(*Q*PPh_2_
)_2_}_3_]Br. *Inorganic Chemistry*.

[429] Fedin VP, Sokolov MN, Geras'ko OA (1990). Triangular W_3_
S_7_
^4^
^+^
and W_3_
S_4_
^4^
^+^ complexes. *Inorganica Chimica Acta*.

[430] Fedorov VE, Geras'ko OA, Mironov YuV (1995). *Journal of Structural Chemistry*.

[431] Garriga JM, Llusar R, Uriel S (2003). Synthesis and third-order nonlinear optical properties of [Mo_3_
(*μ*
_3_
−S)(*μ*
_2_
−S_2_
)_3_]^4+^
clusters with maleonitriledithiolate, oxalate and thiocyanate ligands. *Dalton Transactions*.

[432] Fedin VP, Mironov YV, Virovets AV, Podberezskaya NV, Federov VYe (1992). Synthesis and X-ray structure of the triangular cluster (Et_4_
N){[Mo_3_
(*μ*
_3_
−S)(*μ*
_2_
−S_2_)_3_
(NH_2_
Ph)_3_Br_3_]br}br. *Polyhedron*.

[433] Sokolov MN, Geras'ko OA, Solodovnikov SF, Fedin VP (2004). Synthesis and crystal structure of [Th_2_
(*μ*−SO_4_
)_2_(DMSO)_12_
]{[Mo_3_S_7_
Br_5_
(DMSO)]Br}_2_
⋅ 2DMSO ⋅ PhCN. *Journal of Structural Chemistry*.

[434] Ledesma GN, Lang ES, Vázquez-López EM, Abram U (2004). Synthesis and characterization of the first aryltellurium(II) halide complex stabilized by a Te–Te bond from a tellurium ether. *Inorganic Chemistry Communications*.

[435] Lang ES, Fernandes RM, Silveira ET, Abram U, Vázquez-López EM (1999). Structures of iodophenyltellurium(II) and diiododi-(*β*-naphtyl)tellurium(IV). *Zeitschrift für anorganische und allgemeine Chemie*.

[436] Boyle PD, Cross WI, Godfrey SM (2000). Synthesis and characterization of Ph_4_
Te_4_
I_4_, containing a Te_4_ square, and Ph_3_
PTe(Ph)I. *Angewandte Chemie International Edition*.

[437] Lin X, Chen H-Y, Chi L-S, Zhuang H-H (1998). Synthesis and crystal structures of two new trinuclear molybdenum cluster compounds containing a [Mo_3_
Te_7_]^4^
fragment. *Polyhedron*.

[438] Chen H, Lin X, Chi L, Lu C, Zhuang H, Huang J (2000). Synthesis and crystal structures of new triangle tungsten telluride compounds 
containing a [W_3_
(*μ*
_3_
−Te
)(*μ*
_2_
−Te_2_)_3_
]^4+^
cluster core: {W_3_
Te_7_[(RO)_2_
PS_2_]_3_}I(R=
Et,Pr(i)). *Inorganic Chemistry Communications*.

[439] Jones PG, Jeske J

[440] Vikane O (1975). *Acta Chemica Scandinavica, Series A*.

[441] Dakternieks D, di Giacomo R, Gable RW, Hoskins BF (1988). Investigation of organoyltellurium(IV) halide (dithiolate) complex, crystal
structure of di(2-iodo-2
*λ*
^4^-benzotellurol-2-yl diethyldithiocarbamate), [C_8_
H_8_Te(I)(S_2_
CNEt_2_)]_2_. *Journal of Organometallic Chemistry*.

[442] García-Montalvo V, Marcelo-Polo A, Montoya R, Toscano RA, Hernández-Ortega S, Cea-Olivares R (2001). Synthesis, spectroscopic characterization and structural studies of dialkyl dithiophosphinate and 
*N*,*N*-dialkyl dithio- and monothio-carbamate derivatives of 1-iodo-1,1,2,3,4,5-hexahydrotellurophene. *Journal of Organometallic Chemistry*.

[443] García-Montalvo V, Toscano RA, Badillo-Delgado A, Cea-Olivares R (2001). Synthesis, characterization and crystal structure of 1,3-dihydro-2*λ*
^4^-benzotellurole-2,2-diyl bis(*N*-piperidine-dithiocarbamate), [1,2 − C_6_H_4_(CH_2_)_2_
Te(S_2_CNC_5_H_10_)_2_] (1), 1,3 dihydro-2*λ*
^4^
-benzotellurole-2-iodo-2-yl -diethyldithiophosphinate. *Polyhedron*.

[444] Demartin F, Devillanova FA, Isaia F, Lippolis V, Verani G (1997). Reaction of *N*,*N*
′-dimethylimidazolidine-2-selone (L) with I_2_. Crystal structure of the mixed-valence (L ⋅ I_2_)(L_2_)^2+^
⋅ 2I_3_
^−^ compound. *Inorganica Chimica Acta*.

[445] Maoyu S, Jinling H, Jiaxi L (1985). *Science in China, Series B*.

[446] Shao-Fang L, Jian-Quan H, Yu-Hui L, Jin-Ling H (1987). *Acta Chimica Sinica*.

[447] Can-Zhong L, Wen T, Hong-Hui Z, Ding-Ming W (1993). *Chinese Journal of Structural Chemistry*.

[448] Maoyu S, Jinling H, Jiaxi L (1984). *Chinese Journal of Structural Chemistry*.

[449] Lu S-F, Huang J-Q, Chen H-B, Wu Q-J (1993). *Acta Chimica Sinica*.

[450] Man-Fang W, Guo-Cong G, Jin-Shun H, Hong-Hui Z, Qian-Er Z, Jia-Xi L (1994). *Chinese Journal of Structural Chemistry*.

[451] Chen J, Lu S-F, Huang Z-X, Yu R-M, Wu Q-J (2001). Synthesis and structural characterization of the novel cluster 
compound {[Mo_3_S_7_(dtp)_3_]_4_
⋅ I}{(HgI_3_)_3_} ⋅ 4H_2_
O(dtp = 
S_2_P(OC_2_H_5_
)_2_
^−^). *Chemistry - A European Journal*.

[452] Lu C-Z, Zhuang J-N, Chi L-S (1999). A quasi-layer structure of trinuclear molybdenum(IV) cluster, [Mo_3_S_7_
(S_2_CNEt_2_)_3_
]I−S_8_. *Journal of Chemical Crystallography*.

[453] Mayor-López MJ, Weber J, Hegetschweiler K (1998). Structure and reactivity of [Mo_3_
−*μ*
_3_
S−(*μ*S_2_)
_2_]^4+^
complexes. Quantum chemical calculations, X-ray structural characterization, and Raman spectroscopic measurements. *Inorganic Chemistry*.

[454] Zimmermann H, Hegetschweiler K, Keller T (1991). Preparation of complexes containing the [Mo_3_
S(S_2_
)_3_]^4+^
core and structure of tris(diethyldithiocarbamato)tris(*μ*-disulfido)(*μ*
_3_-thio)-triangulo-trimolybdenum(IV) iodide. *Inorganic Chemistry*.

[455] Aragoni MC, Arca M, Demartin F (2002). Anti-thyroid drug methimazole: X-ray characterization of two novel ionic disulfides obtained from its chemical oxidation by I_2_. *Journal of the American Chemical Society*.

[456] Čmelík R, Marek J, Pazdera P (2002). Regioselectivity of electrophilic attacks to 5-amino-3-thioxo-3H-1,2-dithiole-4-carboxylic acid functional derivatives. Elucidation of product structures. *Heterocyclic Communications*.

[457] Young CG, Kocaba TO, Yan XF (1994). Bridging disulfido complexes of molybdenum and tungsten formed by reductive sulfurization of oxo-molybdenum(VI) complexes and reductive desulfurization of thio(disulfido)-tungsten(VI) complexes. *Inorganic Chemistry*.

[458] Fedin VP, Müller A, Bogge H (1993). *Russian Journal of Inorganic Chemistry*.

[459] Demartin F, Deplano P, Devillanova FA, Isaia F, Lippolis V, Verani G (1993). Conductivity, FT-Raman spectra, and X-ray crystal structures of two 
novel [D_2_I]I_*n*_ (*n* = 
3 and D = 
*N*-methylbenzothiazole-2(3H)-selone; *n* = 
7 and D =
*N*-methylbenzothiazole-2(3H)-thione) iodonium salts. First example of I^−^⋅3
I_2_ heptaiodide. *Inorganic Chemistry*.

[460] Fox S, Stibrany RT, Potenza JA, Schugar HJ (2001). A novel mixed-valence trinickel complex containing nickel(I), nickel(II) and iodonium moieties. *Inorganica Chimica Acta*.

[461] Nosco DL, Heeg MJ, Glick MD, Elder RC, Deutsch E (1980). Coordination stabilization of organic intermediates. Crystal structure of {[(en_2_)Co(SCH_2_CH_2_NH_2_)]_2_I}(NO_3_)_5_ ⋅ 4
H_2_
O, a stable complex of iodine(I). *Journal of the American Chemical Society*.

[462] Lin GH-Y, Hope H (1972). The crystal structure of bis(thiourea)iodine(I) iodide. *Acta Crystallographica, Section B*.

[463] Boyle PD, Christie J, Dyer T (2000). Further structural motifs from the reactions of thioamides with diiodine and the interhalogens iodine monobromide and iodine monochloride: an FT-Raman and crystallographic study. *Journal of the Chemical Society, Dalton Transactions*.

[464] Daga V, Hadjikakou SK, Hadjiliadis N, Kubicki M, Dos Santos JHZ, Butler IS (2002). Synthesis, spectroscopic and structural characterization of novel diiodine adducts with the heterocyclic thioamides, thiazolidine-2-thione (tzdtH), benzothiazole-2-thione (bztzdtH) and benzimidazole-2-thione (bzimtH). *European Journal of Inorganic Chemistry*.

[465] Minkwitz R, Preut H, Sawatzki J (1988). *Zeitschrift für Naturforschung, Section B*.

[466] Seppälä E, Ruthe F, Jeske J, Du Mont W-W, Jones PG (1999). Coordination and oxidation of phosphine selenides with iodine: from cation pairs [(R_3_PSe)_2_
I^+^]_2_ to (iodoseleno)phosphonium ions [R_3_
PSeI]^+^ existing as guests in polyiodide matrices. *Chemical Communications*.

[467] Jones PG, Jeske J

[468] Kiriyama H, Mizuhashi Y, Ootani J (1986). Crystal structures of trimethylammonium hexaiodotellurate(IV) and heptaiodotellurate(IV). *Bulletin of the Chemical Society of Japan*.

[469] Boyle PD, Godfrey SM, Pritchard RG (1999). The reaction of *N*-methylbenzothiazole-2-selone and 1,1-dimethylselenourea with sulfuryl chloride and dichlorine. *Journal of the Chemical Society, Dalton Transactions*.

[470] Hauge S, Marøy K, Odegard T (1988). *Acta Chemica Scandinavica, Series A*.

[471] Krebs B, Ahlers F-P, Lührs E (1991). Synthese, Struktur und Eigenschaften der neuen Bromoselenate(II) [Se_3_
Br_8_]^2−^
,[Se_4_Br_14_]^2−^
und [Se_5_Br_12_
]^2−^ Kristallstrukturen von [Cu(i − PropCN)_4_
]_2_[Se_3_
Br_8_],[EtPh_3_
P]_2_[Se_4_Br_14_
] und [n − Prop_4_
N]_2_[Se_5_
Br_12_
]. *Zeitschrift für anorganische und allgemeine Chemie*.

[472] Janickis V, Törnroos KW, Herberhold M, Songstad J, Milius W (2002). Reaction of selenium with bromine (Se : Br =
1 : 1) in acetonitrile in the presence of tetramethylammonium bromide: synthesis and crystal structure of [(CH_3_)_4_
N]_2_[Se_16_Br_18_
], the salt of a unique bromoselenate(I) anion. *Zeitschrift für anorganische und allgemeine Chemie*.

[473] Favier F, Pascal JL, Belin C, Tillard-Charbonnel M (1997). A new pentachlorotellurate(IV): *catena*-poly[hexakis(acetonitrile)aluminium 
tris-[tetrachlorotellurate(IV)-*μ*-chloro] acetonitrile]. *Acta Crystallographica, Section C*.

[474] Lippolis V, Isaia F, Devillanova FA (2006). *Handbook of Chalcogen Chemistry*.

[475] Aragoni MC, Arca M, Devillanova FA (2003). First example of an infinite polybromide 2D-network. *Chemical Communications*.

[476] McCullough JD, Knobler C (1976). Crystal and molecular structure of 2-biphenylyltellurium triiodide C_12_
H_9_TeI_3_. *Inorganic Chemistry*.

[477] McCullough JD (1977). Crystal and molecular structure of the *β* modification of 2-biphenylyltellurium triiodide, C_12_
H_9_TeI_3_. *Inorganic Chemistry*.

[478] Appa Rao GVN, Seshasayee M, Aravamudan G, Radha K (1983). Structure of bis[bis(2-hydroxyethyl)dithiocarbamato]diiodotellurium(IV), [Te(C_5_H_10_NO_2_S_2_)_2_I_2_]. *Acta Crystallographica, Section C*.

[479] Kiriyama H, Nishizaki K (1986). Crystal structure and molecular motion of tetramethylammonium hexaiodotellurate(IV-iodine (1/1) compound. *Bulletin of the Chemical Society of Japan*.

[480] Bird PH, Kumar V, Pant BC (1980). Crystal and molecular structures of the (4-alkoxyphenyl)tellurium(IV) trihalides: (4-EtOPh)TeCl_3_, (4-EtOPh)TeBr_3_, and (4-MeOPh)TeI_3_. *Inorganic Chemistry*.

[481] Närhi SM, Oilunkaniemi R, Laitinen RS, Ahlgrén M (2004). The reactions of tellurium tetrahalides with triphenylphosphine under ambient conditions. *Inorganic Chemistry*.

[482] Kumar RK, Aravamudan G, Udupa MR, Seshasayee M, Hamor TA (1993). Structure of bis(diethyldithiocarbamato)diiodotellurium(IV). *Acta Crystallographica, Section C*.

[483] Lang ES, Manzoni de Oliveira G, Silveira ET, Burrow RA, Vázquez-López EM (2002). Crystal and molecular structure of (*α*-naphthyl)TeI_3_. *Journal of Organometallic Chemistry*.

[484] Chao GY, McCullough JD (1961). The refinement of the structure of the complex of iodine with 1,4-diselenane, C_4_
H_8_
Se_2_ ⋅ 2I_2_. *Acta Crystallographica*.

[485] Jones PG, Jeske J

[486] Kuhn N, Fawzi R, Kratz T, Steimann M, Henkel G (1996). Zur oxidation von 2-selenoimidazolinen mit iod. *Phosphorus, Sulfur and Silicon and Related Elements*.

[487] Kubiniok S, du Mont W-W, Pohl S, Saak W (1988). The reagent diphenyldiselane/iodine: no phenylselenenyl iodide but a charge transfer complex with cyclic moieties. *Angewandte Chemie International Edition*.

[488] Jeske J, du Mont W-W, Jones PG (1999). Iodophosphane selenides: building blocks for supramolecular soft-soft chain, helix, and base-pair arrays. *Chemistry - A European Journal*.

[489] du Mont W-W, Martens A, Pohl S, Saak W (1990). Reversible dismutation and coordination of bis(2,4,6-triisopropylphenyl) diselenide with iodine: a model study that relates to iodine intercalation between selenium chains. *Inorganic Chemistry*.

[490] Cristiani F, Demartin F, Devillanova FA, Isaia F, Saba G, Verani G (1992). An X-ray, spectroscopic and semiempirical quantum-mechanical study on complexes of thiones and selones with molecular diiodine. *Journal of the Chemical Society, Dalton Transactions*.

[491] Maddox H, McCullough JD (1966). The crystal and molecular structure of the iodine complex of 1-oxa-4-selenacyclohexane, C_4_
H_8_
OSe ⋅ I_2_. *Inorganic Chemistry*.

[492] Godfrey SM, Jackson SL, McAuliffe CA, Pritchard RG (1997). Reaction of R_3_PSe with I_2_
; crystal structures of Ph_3_PSel_2_
, (Me_2_
N)_3_PSel_2_
and (Et_2_N)_3_
PSel_2_, the first crystallographically characterised charge-transfer complexes of tertiary phosphine selenides with diiodine. *Journal of the Chemical Society, Dalton Transactions*.

[493] Hursthouse MB, Hibbs DE, Bricklebank N

[494] Rudd MD, Lindeman SV, Husebye S (1997). Three-centre, four-electron bonding and structural characteristics of two-coordinate iodine(I) 
complexes with halogen and chalcogen ligands. Synthesis, spectroscopic characterization and X-ray 
structural studies of (triiodo)[tris(dimethylamino)phosphaneselenide]iodine(I) and bis {(triiodo)[tri(N-morpholyl)phosphaneselenide]−
iodine(I)}/diiodine molecular complex. *Acta Chemica Scandinavica*.

[495] Godfrey SM, McAuliffe CA, Pritchard RG, Sarwar S (1997). *Journal of the Chemical Society, Dalton Transactions*.

[496] Arca M, Cristiani F, Devillanova FA (1997). Reactivity of 1,3,5-trithiacyclohexane and 1,3,5-triselenacyclohexane towards molecular diiodine. Crystal structures of the diiodine adducts. *Polyhedron*.

[497] Hope H, McCullough JD (1964). The crystal structure of the molecular complex of iodine with tetrahydroselenophene, C_4_
H_8_Se.I_2_. *Acta Crystallographica*.

[498] Bigoli F, Deplano P, Devillanova FA (1994). Reaction of imidazole-2-selone derivatives with diiodine - synthesis, structural and spectroscopic characterization of the adduct 1,1′-bis(3-methyl-4-imidazolin-2- selone)methane bis(diiodine) and of the 1st examples of I-Se-I hypervalent seleniumcompounds- 1,3-dimethyl-4-imidazolin-2-ylium diiodo selenanide and 1,2-bis(3-methyl- 4-imidazolin-2-ylium diiodo selenanide)-ethane bis(dichloromethane). *Gazzetta Chimica Italiana*.

[499] Arca M, Demartin F, Devillanova FA (1999). A new assembly of diiodine molecules at the triphenylphosphine sulfide template. *Journal of the Chemical Society, Dalton Transactions*.

[500] Rømming C (1960). The crystal structure of the 1:1 addition compound formed by benzyl sulphide and iodine. *Acta Chemica Scandinavica*.

[501] Herbstein FH, Schwotzer W (1984). Crystal structures of polyiodide salts and molecular complexes. 7. Interaction of thiones with molecular diiodine. The crystal structures of dithizone-diiodine, ethylenethiourea-bis(diiodine), bis(ethylenethiourea)-tris(diiodine), bis(dithizone)-heptakis(diiodine), and 1-(1-imidazolin-2-yl)-2-thioxoimidazolidinium triiodide-(ethylenethiourea-diiodine). *Journal of the American Chemical Society*.

[502] Bigoli F, Deplano P, Ienco A (1999). Structure and bonding of diiodine adducts of the sulfur-rich donors 1,3-dithiacyclohexane-2-thione (ptc) and 4,5-ethylenedithio-1,3-dithiole-2-thione (ttb). *Inorganic Chemistry*.

[503] Chao GY, McCullough JD (1960). The refinement of the structure of the complex of iodine with 1,4 dithiane, C_4_H_8_S_2_
.2I_2_. *Acta Crystallographica*.

[504] Herbstein FH, Ashkenazi P, Kaftory M, Kapon M, Reisner GM, Ginsburg D (1986). Propellanes LXXIX. Comparison of the geometries of dithia[*n*.3.3]propellanes (*n* = 
1,2,3) and dithia(and oxathia)[4.3.3]propellanes. Study of the influence of complexation with HgCl_2_,I_2_,CdCl_2_
and PdCl_2_ and of formation of sulfoxides on some of these compounds. Demonstration of the ‘klammer’ effect. Structures of eighteen crystals. *Acta Crystallographica, Section B*.

[505] Bricklebank N, Coles SJ, Forder SD, Hursthouse MB, Poulton A, Skabara PJ (2005). Diiodine complex of diferrocenyl(phenyl)phosphine sulfide: the structural and electrochemical behaviour of Fc_2_
(Ph)PS ⋅ I_2_. *Journal of Organometallic Chemistry*.

[506] Freeman F, Ziller JW, Po HN, Keindl MC (1988). Reactions of imidazole-2-thiones with molecular iodine and the structures of two crystalline modifications of the 1:1 1,3-dimethylimidazole-2-thione-diiodine charge-transfer complex (C_5_
H_8_I_2_
N_2_S). *Journal of the American Chemical Society*.

[507] Arca M, Demartin F, Devillanova FA (1998). Synthesis, X-ray crystal structure and spectroscopic characterization of the new dithiolene [Pd(Et_2_timdt)_2_] and of its adduct with molecular diiodine [Pd(Et_2_timdt)_2_] ⋅ I_2_
⋅ CHCl_3_
(Et_2_timdt=monoanion of 1,3-diethylimidazolidine-2,4,5-trithione). *Journal of the Chemical Society, Dalton Transactions*.

[508] Atzei D, Deplano P, Trogu EF, Bigoli F, Pellinghelli MA, Vacca A (1988). Interaction of diiodine with some tetra-substituted dithiooxamides. Crystal and molecular structure of bis(morpholinothiocarbonyl)bis(diiodine). *Canadian Journal of Chemistry*.

[509] Antoniadis CD, Corban GJ, Hadjikakou SK (2003). Synthesis and characterization of (PTU)I_2_(PTU=
6−*n*−propyl−2−thiouracil) and (CMBZT)I_2_ (CMBZT = 5-chloro-2-mercaptobenzothiazole) and possible implications for the mechanism of action of anti-thyroid drug. *European Journal of Inorganic Chemistry*.

[510] Lee L, Crouch DJ, Wright SP (2004). Supramolecular polymers of 4,5-bis(bromomethyl)-1,3-dithiole-2-thione—dihalogen adducts. *CrystEngComm*.

[511] Allshouse J, Haltiwanger RC, Allured V, DuBois MR (1994). Molecular and polymeric compounds resulting from Lewis acid interactions 
with [CpMo(*μ*−
S)N-t-Bu]_2_. *Inorganic Chemistry*.

[512] Lyon EJ, Musie G, Reibenspies JH, Darensbourg MY (1998). Sulfur site iodine adduct of a nickel thiolate complex. *Inorganic Chemistry*.

[513] Kiel G (1981). *Zeitschrift für Naturforschung, Section B*.

[514] Baker PK, Harris SD, Durrant MC, Hughes DL, Richards RL (1995). Preparation and structural characterization of the charge-transfer complex (12[ane]S_4_
.I_2_)*_∞_* (12[ane]S_4_
=1,4,7,10-tetrathiacyclododecane). *Acta Crystallographica, Section C*.

[515] Bois d'Enghien-Peteau M, Meunier-Piret J, van Meerssche M (1968). *Journal de Chimie Physique et de Physico-Chimie Biologique*.

[516] Khitrich NV, Seifullina II, Starikova ZA (2002). *Russian Journal of Inorganic Chemistry*.

[517] Ito S, Liang H, Yoshifuji M (2003). Preparation, structure, and some coordination properties of 2-chloro-3,3-diphenyl-3-thioxo-1-(2,4,6-tri-t-butylphenyl)-1,3-diphosphapropene. *Chemical Communications*.

[518] Apperley DC, Bricklebank N, Hursthouse MB, Light ME, Coles SJ (2001). Vibrational, 31P NMR and crystallographic studies of diiodine adducts of some bidentate tertiary phosphine sulfides. *Polyhedron*.

[519] Bock H, Nagel N, Seibel A (1997). *Liebigs Annalen*.

[520] Yamamoto M, Wu LP, Kuroda-Sowa T, Maekawa M, Suenaga Y, Munakata M (1997). Preparation, characterization and X-ray crystal structures of I_2_ and copper(II) complexes of 2,11-dithia[3.3]paracyclophane. *Inorganica Chimica Acta*.

[521] Apperley DC, Bricklebank N, Burns SL, Hibbs DE, Hursthouse MB, Malik KMA (1998). Crystal structure of triphenylphosphine sulfide diiodine; the first crystallographically characterised 11 molecular charge-transfer complex of a tertiary phosphine sulfide with diiodine. *Journal of the Chemical Society, Dalton Transactions*.

[522] Aragoni MC, Arca M, Demartin F (2004). A theoretical investigation of the donor ability of [M(R,R′timdt)_2_
] dithiolene complexes towards molecular diiodine (M = Ni, Pd, Pt; R,R′timdt = formally monoreduced disubstituted imidazolidine-2,4,5-trithione). *European Journal of Inorganic Chemistry*.

[523] Allen DW, Berridge R, Bricklebank N (2003). Structural and magnetic properties of a novel ferrocenyl-diiodine charge transfer complex. *Inorganic Chemistry*.

[524] Bigoli F, Deplano P, Mercuri ML, Pellinghelli MA, Trogu EF (1992). Spectrophotometric study, crystal and molecular structure of the 1:1 complex between 1,3-dithiolane-2-thione and diiodine. *Phosphorus, Sulfur, and Silicon and the Related Elements*.

[525] Blake AJ, Gould RO, Radek C, Schröder M (1993). The synthesis and low-temperature single crystal X-ray structure of the charge-transfer complex ([9]aneS_3_)_2_(I_2_)_4_([9]aneS_3_
=
1,4,7-trithiacyclononane). *Chemical Communications*.

[526] Cristiani F, Devillanova FA, Isaia F, Lippolis V, Verani G, Demartin F (1993). Spectroscopic studies and X-ray crystal structures of charge-transfer complexes of 1,4,7-trithiacyclononane with diiodine. *Heteroatom Chemistry*.

[527] Blake AJ, Cristiani F, Devillanova FA (1997). Structural and solution studies of diiodine charge-transfer complexes of thioether crowns. *Journal of the Chemical Society, Dalton Transactions*.

[528] Bigoli F, Deplano P, Mercuri ML, Pellinghelli MA, Trogu EF (1992). Synthetic, structural and spectroscopic studies of the donating properties of sulphur-rich molecules towards I_2_: X-ray structure of 1,3-dithiole-2-thione diiodine. *Phosphorus, Sulfur, and Silicon and the Related Elements*.

[529] Blake AJ, Devillanova FA, Garau A (1998). Thioether—iodine charge-transfer complexes. Synthesis and low-temperature single-crystal structures of complexes of penta-, hexa- and octa-dentate homoleptic thioether macrocycles. *Journal of the Chemical Society, Dalton Transactions*.

[530] Hartl H, Steidl S (1977). *Zeitschrift für Naturforschung, Section B*.

[531] Schollhammer P, Pétillon FY, Talarmin J, Muir KW, Fun HK, Chinnakali K (2000). Halogenation and alkylation at a Mo_2_
^III^(*μ*−S) site. Crystal structure of the metal-sulfenyl halide 
complex [Mo_2_
(*η*
^5^-C_5_Me_5_)_2_
(*μ*−SMe)_2_(*μ*−SI)(CO)_2_
]I_5_. *Inorganic Chemistry*.

[532] Bock H, Rauschenbach A, Näther C, Kleine M, Havlas Z (1996). Kristallzüchtung und Strukturbestimmung von Donator/Akzeptor-Komplexen aus 1,2,4,5-Tetrakis(alkylthio)benzolen und Brom oder Iod. *Liebigs Annalen*.

[533] Blake AJ, Li W-S, Lippolis V, Schröder M (1997). 1,4,8,11-Tetrakis(diiodine)-1,4,8,11-tetrathiacyclotetradecane. *Acta Crystallographica, Section C*.

[534] Leung W-H, Chim JLC, Hou H, Hun TSM, Williams ID, Wong W-T (1997). Oxidation reactions of dithiocarbamate complexes of ruthenium(II). *Inorganic Chemistry*.

[535] Blake AJ, Gould RO, Li W-S (1998). Silver-thioether crown complexes as templates for the synthesis of extended polyiodide networks: synthesis and X-ray crystal structures of [Ag_2_
([15]aneS_5_
)_2_]I_12_
, [Ag([18]aneS_6_)]I_7_
, [Ag([18]aneS_6_)]I_3_
, and [Ag([9]aneS_3_
)]I_5_. *Inorganic Chemistry*.

[536] Ahlsen EL, Strømme KO (1974). The crystal structure of the addition compound *N*-methylthiocaprolactam-iodine (1:1). *Acta Chemica Scandinavica, Series A*.

[537] Heuer WB, Pearson WH (1996). Synthesis and characterization of nickel-group bis(dithiocroconate) complexes and dicyanomethylene-substituted analogues. *Journal of the Chemical Society, Dalton Transactions*.

[538] Corban GJ, Hadjikakou SK, hadjiliadis N (2005). Synthesis, structural characterization, and computational studies of novel diiodine adducts with the heterocyclic thioamides *N*-methylbenzothiazole-2-thione and benzimidazole-2-thione: implications with the mechanism of action of antithyroid drugs. *Inorganic Chemistry*.

[539] Bigoli F, Deplano P, Mercuri ML (1996). Evaluation of thermodynamic parameters for highly correlated chemical systems: a spectrophotometric study of the 1:1 and 2:1 equilibria between I_2_ and 1,1′-methylenebis(3-methyl-4-imidazoline-2-thione)(mbit) and 1,1′-ethylenebis(3-methyl-4-imidazoline-2-thione)(ebit). Crystal and molecular structures of mbit.2I_2_ and ebit.2I_2_. *Journal of the Chemical Society, Dalton Transactions*.

[540] Bransford JW, Meyers EA (1978). Bis(triphenylphosphinesulfide-S-iodine, C_36_
H_30_I_6_
P_2_S_2_. *Crystal Structure Communications*.

[541] Bigoli F, Deplano P, Devillanova FA (1997). Syntheses, X-ray crystal structures, and spectroscopic properties of new nickel dithiolenes and related compounds. *Inorganic Chemistry*.

[542] Lu FL, Keshavarz M, Srdanov G, Jacobson RH, Wudl F (1989). A new preparation of 5-(alkylthio)-1,2-dithiole-3-thiones and a highly functionalized 1,3-dithiole-2-thione. *Journal of Organic Chemistry*.

[543] Srdanov G, Wudl F (1988). *Polymeric Materials: Science and Engineering*.

[544] Atzei D, Deplano P, Trogu EF (1989). Interaction of diiodine with Et_4_
todit = 4,5,6, 7-tetrathiocino [1,2−*b*:3
,4−*b*′]diimidazolyl-1,3,8,10-tetraethyl-2,9-dithione. Crystal and molecular structure of 
Et_4_
todit ⋅ _2_I_2_. *Canadian Journal of Chemistry*.

[545] Tipton AL, Lonergan MC, Stern CL, Shriver DF (1992). Structure, conductivity and Raman spectrum of 4,7,13,16-tetraoxa-1,10-dithiacyclooctadecanebis(diiodine). *Inorganica Chimica Acta*.

[546] Serpe A, Bigoli F, Cabras MC (2005). Pd-dissolution through a mild and effective one-step reaction and its application for Pd-recovery from spent catalytic converters. *Chemical Communications*.

[547] Bigoli F, Deplano P, Mercuri ML (1995). Evaluation of thermodynamic parameters on higly correlated chemical systems: a spectrophotometric study of the 1:1 and 1:2 equilibria between I_2_
and R_4_todit = 4
,5,6,7-tetrathiocino[1,2−
*b*:3
,4−*b*′]diimidazolyl-1,3,8,10-tetraalkyl-2,9-dithione; (R = 
Bu, Me (new data); Et, Ph (reinvestigation)). Crystal and molecular structure of the charge-transfer 
complex Bu_4_
todit ⋅ 2I_2_. *Canadian Journal of Chemistry*.

[548] Cristiani F, Devillanova FA, Isaia F, Lippolis V, Verani G, Demartin F (1995). Charge transfer complexes of benzoxazole-2(3*H*)-thione and benzoxazole-2(3*H*)-selone with diiodine: X-ray crystal structure of benzoxazole-2(3*H*)-thione bis(diiodine). *Polyhedron*.

[549] Lee JQ, Sampson ML, Richardson JF, Noble ME (1995). Halogenation at a dimolybdenum(V) and ditungsten(V) sulfur bridge: metallosulfenyl
halides M_2_(*μ*−SX) and [M_2_(*μ*−SX_3_)]n. Charge-transfer interactions. *Inorganic Chemistry*.

[550] Blake AJ, Devillanova FA, Garau A (1999). Structural and spectroscopic studies of charge-transfer adducts formed between IBr and thioether crowns. *Journal of the Chemical Society, Dalton Transactions*.

[551] McCullough JD, Knobler C, Baker C, Hope H (1971). Crystal and molecular structure of the iodine monobromide complex of 1,4-dithiane, C_4_H_8_S_2_
.2IBr. *Inorganic Chemistry*.

[552] Skabara PJ, Bricklebank N, Berridge R (2000). Crystal engineering towards highly ordered polymeric structures of 1,3-dithiole-2-thione—dihalogen adducts. *Journal of the Chemical Society, Dalton Transactions*.

[553] Asseily GA, Davies RP, Rzepa HS, White AJP (2005). A solid-state structural and theoretical study on the 1:1 addition compounds of thioethers with dihalogens and interhalogens I-X(X=
I,Br,Cl). *New Journal of Chemistry*.

[554] Arca M, Devillanova FA, Garau A (1998). 31P CP-MAS NMR, vibrational, and X-ray characterization of the adducts of triphenylphosphine sulfide with ICl and IBr. *Zeitschrift für anorganische und allgemeine Chemie*.

[555] Demartin F, Devillanova FA, Garau A, Isaia F, Lippolis V, Verani G (1999). Reactions of *N*-methylbenzothiazole-2(3*H*)-thione (1) and -selone (2) with ICl synthesis and X-ray crystal structures of the charge-transfer adducts 1 ⋅ ICl (I) and 2 ⋅ ICl (II). *Polyhedron*.

[556] Knobler C, McCullough JD (1968). Crystal and molecular structure of the iodine monochloride complex of 1-oxa-4-selenacyclohexane, C_4_H_8_OSe.ICl. *Inorganic Chemistry*.

[557] Jeske J, du Mont W-W, Ruthe F, Jones PG, Mercuri LM, Deplano P (2000). Properties of chalcogen-chalcogen bonds, 23
novel mesityltellurium cations from selenenation and tellurenation reactions of dimesityl telluride in the presence of the Br_2_
/AgSbF_6_ reagent. *European Journal of Inorganic Chemistry*.

[558] Hague S, Maroy K (1998). *Acta Chemica Scandinavica*.

[559] Nakanishi W, Hayashi S, Yamaguchi S, Tamao K (2004). First Br_4_
four centre—six electron and Se_2_
Br_5_ seven centre–ten electron bonds in nonionic bromine adducts of selenanthrene. *Chemical Communications*.

[560] Hammerschmidt A, Beckmann I, Läge M, Krebs B (2004). A novel crown-ether stabilized oxonium halogenochalcogenate(IV): [H_7_
O_3_(Bis-dibromo-dibenzo-30-crown-10] [Se_2_
Br_9_] ⋅ 1.5CH_2_
Cl_2_. *Zeitschrift für Naturforschung, Section B*.

[561] Hasche S, Reich O, Beckmann I, Krebs B (1997). Stabilisierung von Oxohalogeno- und Halogenochalkogen(IV)-Säuren durch Protonenakzeptoren - Darstellung, Struktur und Eigenschaften von [C_4_
H_10_
NO]_2_[SeOCl_4_], [C_4_
H_10_NO]_2_[Se_2_Br_10_] und [(CH_3_)_2_CHC(NH_2_)(OH)][Te_3_
Cl_13_] ⋅ (CH_3_)_2_CHCN. *Zeitschrift für anorganische und allgemeine Chemie*.

[562] Regelmann B, Klinkhammer KW, Schmidt A (1997). (CH_3_)_2_
SBr_2_
^−^ einige Reaktionen und Strukturen. *Zeitschrift für anorganische und allgemeine Chemie*.

[563] Bock H, Havlas Z, Rauschenbach A, Näther C, Kleine M (1996). Structurally recognizable electron density transfer in the donor–acceptor complex {1,2,4,5-tetra(thioethyl) benzenebromine2}infity. *Chemical Communications*.

[564] Allegra G, Wilson GE, Benedetti E, Pedone C, Albert R (1970). Structure of a halosulfonium salt. The 1:1 adduct of thiophane with bromine. *Journal of the American Chemical Society*.

[565] Haas A, Kasprowski J, Pryka M (1992). *Chemische Berichte*.

[566] Gysling HJ, Luss HR, Gardner SA (1980). Organotellurium(IV) complexes: synthesis and molecular structure of 2,6-diacetylpyridine (C,N,O) tellurium(IV) trichloride. *Journal of Organometallic Chemistry*.

[567] Pietikainen J, Laitinen RS, Konu J, Valkonen J (2001). *Zeitschrift für Naturforschung, Section B*.

[568] Teijido MV, Zukerman-Schpector J, Camillo RL (2003). Dichloro[(E)-2-chloro-1-vinyl-cyclohexanol](4-methoxyphenyl)Te(IV). A case of conformational
polymorphism. *Zeitschrift für Kristallographie*.

[569] Kuhn N, Abu-Rayyan A, Eichele K, Piludu C, Steimann M (2004). Weak interionic interactions in 2-haloimidazolium hexahalotellurates(IV) [1]. *Zeitschrift für anorganische und allgemeine Chemie*.

[570] Boese R, Haas A, Hoppmann E, Merz K, Olteanu A (2002). Preparation of acyclic and heterocyclic tellurathianitrogen compounds: contributions to a better understanding of complex reaction pathways. *Zeitschrift für anorganische und allgemeine Chemie*.

[571] Hinrichs W, Mandak D, Klar G (1982). *Crystal Structure Communications*.

[572] Zukerman-Schpector J, Camillo RL, Comasseto JV, Santos RA, Caracelli I (1999). Trichloro[(*Z*)-2-chloro-1,2-diphenylvinyl]tellurium(IV). *Acta Crystallographica, Section C*.

[573] Sato S, Ishida H, Nagae M, Kashino S, Furukawa Y, Weiss A (1998). Cationic motions and crystal structures of 1,3,5-trimethylpyridinium hexachlorometallates [(CH_3_)_2_
C_5_H_3_N(CH_3_)]_2_MCl_6_ (M is Sn and Te) studied by ^1^H NMR and X-ray diffraction. *Journal of Molecular Structure*.

[574] Srivastava PC, Bajpai S, Lath R, Bajpai SM, Kumar R, Butcher RJ (2004). Molecular aggregates, zig-zag 2D-stairs, -ribbons and 3D-supramolecular networks of cyclic telluranes assisted by intermolecular Te ⋯ Cl and Te ⋯ Br secondary bonding. *Polyhedron*.

[575] Abriel W (1986). *Zeitschrift für Naturforschung, Section B*.

[576] Chadha RK, Drake JE, Khan MA (1983). Structure of (*p*-bromophenyl)dichloro(phenyl)tellurium(IV), (C_6_
H_5_)(C_6_
H_4_
Br)TeCl_2_. *Acta Crystallographica, Section C*.

[577] Huang C-K, O'Brien DH, Irgolic KJ, Meyers EA (1982). *Crystal Structure Communications*.

[578] Zukerman-Schpector J, Camillo RL, Comasseto JV, Cunha RLOR, Lemos FCD, Caracelli I (1999). Acetonyldichloro[(*Z*)-2-chloro-1-methyl-2-phenylethenyl]tellurium(IV). *Acta Crystallographica, Section C*.

[579] Hinrichs W, Mandak D, Klar G (1982). *Crystal Structure Communications*.

[580] Alcock NW, Harrison WD (1983). An aryltellurium mixed halide anion. Preparation and crystal and molecular structure of [NBun_4_][PhTeCl_3_I]. *Journal of the Chemical Society, Dalton Transactions*.

[581] Chadha RK, Drake JE, Hencher JL (1983). Structure of bis(*p*-bromophenyl)tellurium(IV) dichloride. *Canadian Journal of Chemistry*.

[582] Jones RH, Hamor TA (1984). X-ray study of bonding in crystalline bis(4-hydroxy-3-methylphenyl)tellurium(IV) dichloride (*β*-isomer). *Journal of Organometallic Chemistry*.

[583] Chadha RK, Drake JE (1984). Structure of dichlorobis(*p*-methoxyphenyl)tellurium(IV), [TeCl_2_(C_7_H_7_
O)_2_]. *Acta Crystallographica, Section C*.

[584] Bergman J, Sidén J, Maartmann-Moe K (1984). Structure elucidation of a zwitterionic 2-oxazoline obtained by cyclofunctionalization of n-acetyldiallylamine with tellurium tetrachloride. *Tetrahedron*.

[585] Cameron TS, Amero RB, Cordes RE (1980). *Crystal Structure Communications*.

[586] Achampong A, Parkins AW (1997). Reaction of tellurium tetrachloride with cyclohexene and the crystal structure of racemic bis(*trans*-2-chlorocyclohexyl)tellurium dichloride. *Journal of the Chemical Society, Dalton Transactions*.

[587] Chadha RK, Drake JE (1984). Reaction of benzyltrimethylsilane with tellurium(IV) chloride. Structure of bis(*p*-trimethylsilyltolyl)tellurium dichloride. *Journal of Organometallic Chemistry*.

[588] Buscher K, Heuer S, Krebs B (1981). *Zeitschrift für Naturforschung, Section B*.

[589] Raston CL, Secomb RJ, White AH (1976). ‘Tellurium acetylacetonates’: crystal structures of 1,1-dichloro-1-telluracyclohexane-3,5-dione and 1-telluracyclohexane-3,5-dione. *Journal of the Chemical Society, Dalton Transactions*.

[590] Chadha RK, Drake JE, Khan MA (1984). Crystal structure of diethylammonium tetrachloro(*p*-phenoxyphenyl)tellurate. *Canadian Journal of Chemistry*.

[591] Ahmed MAK, McWhinnie WR, Hamor TA (1985). Tellurated azobenzenes: the crystal and molecular structure of (2-phenylazophenyl-*C*,7
*N*′)tellurium(IV) trichloride. *Journal of Organometallic Chemistry*.

[592] Chivers T, Enright GD, Sandblom N, Schatte G, Parvez M (1999). Synthesis and reactions of *tert*-butylimidotellurium dihalides: X-ray structures of [Cl_2_Te(*μ*−
N^t^Bu)_2_
TeCl_2_]_3_ and (^t^BuO)_2_
Te(*μ*−
N^t^Bu)_2_
Te(O^t^Bu)_2_. *Inorganic Chemistry*.

[593] Pohl S, Saak W, Krebs B (1985). *Zeitschrift für Naturforschung, Section B*.

[594] Caracelli I (2004). Crystal structure of bis(benzyltriethylammonium) hexachlorotellurate(IV), [C_7_
H_7_(C_2_H_5_)_3_N]_2_(TeCl_6_). *Zeitschrift für Kristallographie: New Crystal Structures*.

[595] Milne J, Philippot E, Maurin M (1984). *Revue de Chimie Minerale*.

[596] Chakravorty R, Irgolic KJ, Meyers EA (1985). Trichloro(2-phenylthiophenyl)tellurium. *Acta Crystallographica, Section C*.

[597] Castellano EE, Zukerman-Schpector J, Ferreira JTB, Comassetto JV (1986). Structure of dichloro[(4,4-dimethyl-5-oxo-2,3,4,5-tetrahydro-2-furyl)methyl](4-methoxyphenyl)tellurium(IV). *Acta Crystallographica, Section C*.

[598] Chadha RK, Drake JE (1985). Crystal structure of dimeric *p*-phenoxyphenyltellurium(IV) trichloride. *Journal of Organometallic Chemistry*.

[599] Michelet A, Toffoli P, Rodier N (1986). Hexachlorotellurate(IV) de bis(triméthyl-2,4,6 pyridinium). *Acta Crystallographica, Section C*.

[600] Abriel W, Friedrich C (1985). *Zeitschrift für Naturforschung, Section B*.

[601] Christofferson GD, Sparks RA, McCullough JD (1958). The crystal structure of *α*-dimethyltellurium dichloride. *Acta Crystallographica*.

[602] Ziolo RF, Troup JM (1983). Experimental observation of the tellurium(IV) bonding and lone-pair density in dimethyltellurium dichloride by x-ray diffraction techniques. *Journal of the American Chemical Society*.

[603] Bergman J, Engman L (1979). Tellurium in organic synthesis V. X-ray structure of 8-ethoxy-4-cyclooctenyltellurium trichloride and its 
relevance to the TeO_2_-oxidation of alkenes. *Journal of Organometallic Chemistry*.

[604] Narhi SM, Oilunkaniemi R, Laitinen RS, Ahlgren M (2004). Bis(triphenyltelluronium) hexachlorotellurate. *Acta Crystallographica, Section E*.

[605] Abriel W (1986). Symmetry rules for the stereochemistry of the lone-pair electrons in Te*X*
_6_
^2^
^−^
(*X* = 
C1,Br,I) and the structures of 1,2-ethanediammonium hexachlorotellurate(IV) and 1,2-ethanediammonium hexachlorostannate(IV). *Acta Crystallographica, Section B*.

[606] Hitchcock PB, Huang Q-G, Lappert MF, Wei X-H (2004). Lanthanide metal amides revisited; the use of the 2,2,6,6-tetramethylpiperidinato (TMP) ligand. *Journal of Materials Chemistry*.

[607] Fernandes RM, de Oliveira GM, Lang ES, Vázquez-López EM (2004). Complex tellurium salts with supramolecular bidimensional lattices: synthesis and X-ray characterization of (2-
Br-
C_5_NH_5_)_2_[TeX_6_](X=
Cl,Br). *Zeitschrift für anorganische und allgemeine Chemie*.

[608] Husebye S, Meyers EA, Zingaro RA, Comasseto JV, Petragnani N (1987). Structure of dichloro(*p*-methoxyphenyl)(8-oxo-7-oxabicyclo[4.3.0]non-5-yl)tellurium(IV)-chloroform (1/0·27). *Acta Crystallographica, Section C*.

[609] Valle G, Russo U, Calogero S (1980). The crystal structure of *bis*-(NNN′N′-tetramethyl)-*α*
*α*′-dithiobisformamidinium hexachlorotellurate(IV): possible effects of hydrogen bondings on the distortion of the hexachlorotellurate(IV) ion. *Inorganica Chimica Acta*.

[610] Russo U, Calogero S, Valle G (1980). *Crystal Structure Communications*.

[611] Batchelor RJ, Einstein FWB, Jones CHW, Sharma RD (1987). X-ray crystal structures of bis(dichlorophenyltelluro)methane and bis(trichlorotelluro)methane. *Organometallics*.

[612] Chadha RK, Drake JE (1986). Crystal structure of bis(*p*-phenoxyphenyl) tellurium dichloride. *Journal of Chemical Crystallography*.

[613] de Matheus M, Torres L, Piniella JF, Briansó JL, Miravittles C (1991). Structure of dichlorobis(*p*-phenoxyphenyl)tellurium(IV). *Acta Crystallographica, Section C*.

[614] Borchers D, Weiss A (1987). *Berichte der Bunsen-Gesellschaft für Physikalische Chemie*.

[615] Zukerman-Schpector J, Haiduc I, Dabdoub MJ (2002). *Zeitschrift für Kristallographie*.

[616] Herrara C, Zingaro RA, Meyers EA (1993). Structure of dimethylammonium N,N,N′,N′-tetramethylformamidinium hexachlorotellurate(IV). *Acta Crystallographica, Section C*.

[617] Wu Y, Ding K, Wang Y, Zhu Y, Yang L (1994). Transmetallation reaction of Schiff-base-type arylmercury compounds with 4-ethoxyphenyltellurium(IV) trichloride and the crystal structure of (4-ethoxyphenyl)[(2-benzylideneamino-5-methyl)phenyl]tellurium(IV) 
dichloride. *Journal of Organometallic Chemistry*.

[618] Borecka B, Cameron TS, Malik MA, Smith BC (1994). Stereospecific addition of aryltellurium(IV) trichlorides to 3-methylcyclohexene: the X-ray crystal structure of *p*-anisyl(trans-2-chloro-*trans*-3-mnethyl-1-cyclohexyl)tellurium(IV) dichloride. *Canadian Journal of Chemistry*.

[619] Zukerman-Schpector J, Camillo RL, Dabdoub MJ, Begnini ML, Caracelli I (1999). Butyldichloro(phenylethynyl)tellurium(IV). *Acta Crystallographica, Section C*.

[620] Zukerman-Schpector J, Camillo RL, Comasseto JV (1999). Dichloro[(*E*)-2-chloro-1-(2-hydroxyprop-2-yl)vinyl](4-methoxyphenyl)tellurium(IV). *Acta Crystallographica, Section C*.

[621] von Deuten K, Schnabel W, Klar G (1980). *Crystal Structure Communications*.

[622] Zukerman-Schpector J, Camillo RL, Caracelli I, Comasseto JV, Cunha RLOR (2002). *Revue Roumaine de Chimie*.

[623] Klapótke TM, Krumm B, Mayer P, Polborn K, Ruscitti OP (2001). Spectroscopic and structural studies on polyfluorophenyl tellurides and tellurium(IV) dihalides. *Inorganic Chemistry*.

[624] Beckmann J, Dakternieks D, Duthie A, Smith NA (2003). Secondary bonding in *para*-substituted diphenyltellurium
dichlorides (*p* −XC_6_H_4_)_2_
TeCl_2_ (X=H, Me, MeO) probed by ^125^Te MAS NMR spectroscopy. Crystal and molecular structure of (*p* −MeC_6_H_4_)_2_TeCl_2_. *Journal of Organometallic Chemistry*.

[625] Mirochnik AG, Bukvetskii BV, Storozhuk TV, Karasev VE (2003). *Russian Journal of Inorganic Chemistry*.

[626] Kandasamy K, Kumar S, Singh HB, Wolmershauser G (2003). Influence of both steric effects and Te⋯N intramolecular nonbonded interactions on the stabilization of organotellurium compounds incorporating [2-[1-(3,5-dimethylphenyl)-2- naphthyl]-4,5-dihydro-4,4-dimethyloxazole]. *Organometallics*.

[627] Lang ES, de Oliveira GM, Fernandes RM, Vázquez-López EM (2004). Supramolecular assembling of complex tellurium salts: synthesis and crystal structures of {Cs[PhTeCl_4_] ⋅ CH_3_OH} and Cs[PhTeBr_4_
]. *Zeitschrift für anorganische und allgemeine Chemie*.

[628] Hey E, Ergezinger C, Dehnicke K (1989). *Zeitschrift für Naturforschung, Section B*.

[629] Abid KY, Al-Salim NI, Greaves M, McWhinnie WR, West AA, Hamor TA (1989). Synthesis and reactions of 1,6-bis(2-butyltellurophenyl)-2,5-diazahexa-1,5-diene and related compounds. The crystal and molecular structures of 2-(butyldichlorotelluro)benzaldehyde and bis[2-(hydroxyiminomethyl)phenyl] ditelluride. *Journal of the Chemical Society, Dalton Transactions*.

[630] Zukerman-Schpector J, Castellano EE, Oliva G, Comasseto JV, Stefani HA (1991). Structure of dichloro[(*Z*)-2-chloro-2-*p*-tolylvinyl](*p*-methoxyphenyl)tellurium(VI). *Acta Crystallographica, Section C*.

[631] Massa W, Lau C, Mühlen M, Neumüller B, Dehnicke K (1998). Communication [Te_6_N_8_ (TeCl_4_)_4_] - tellurium nitride stabilized by tellurium tetrachloride. *Angewandte Chemie International Edition*.

[632] Drews T, Seppelt K (1991). Fe(OTeF_5_)_3_, preparation, structure, and reactivity. *Zeitschrift für anorganische und allgemeine Chemie*.

[633] James MA, Knop O, Cameron TS (1992). Crystal structures of (*n* − *Pr*
_4_N)_2_SnCl_6_, (*n* − *Pr*
_4_N) [TeCl_4_(OH)], (*n* − *Pr*
_4_N)_2_[Te_2_Cl_10_] (nominal), and (*n* − *Pr*
_4_N)_2_ [Se_2_O_2_Cl_6_], with observations on Z_2_L_10_
^2*n*−^ and Z_2_L_8_
^*n*−^ dimers in genera. *Canadian Journal of Chemistry*.

[634] Kozawa K, Uchida T (1993). Structure of benzo[*a*]phenothiazine pentachlorotellurate(IV). *Acta Crystallographica, Section C*.

[635] Beckmann J, Dakternieks D, Duthie A, Mitchell C, Schürmann M (2005). Observation of Te ⋯ *π* and X ⋯ X bonding in *para*-substituted diphenyltellurium dihalides, (*p* − Me_2_NC_6_H_4_)(*p* − YC_6_H_4_)TeX_2_ (X = CI, Br, I;Y=H, EtO, Me_2_N)^−^. *Australian Journal of Chemistry*.

[636] Munzenberg J, Noltemeyer M, Roesky HW (1989). *Chemische Berichte*.

[637] du Bois A, Abriel W (1990). *Zeitschrift für Naturforschung, Section B*.

[638] Roesky HW, Mazzah A, Hesse D, Noltemeyer M (1991). *Chemische Berichte*.

[639] Abe M, Detty MR, Gerlits OO, Sukumaran DK (2004). 21-telluraporphyrins. 3. Synthesis, structure, and spectral properties of a 21,21-dihalo-21-telluraporphyrin. *Organometallics*.

[640] Munzenberg J, Roesky HW, Noltemeyer M, Besser S, Herbst-Irmer R (1993). *Zeitschrift für Naturforschung, Section B*.

[641] Kushch PP, Konovalikhin SV, Shilov GV, Atovmyan LO, Khannanova TA, Lyubovskaya RN (2000). *Russian Chemical Bulletin*.

[642] Barton AJ, Levason W, Reid G, Tolhurst V-A (2000). Synthesis and properties of ditelluroether complexes of osmium, *trans*-[OsCl_2_ (L − L)_2_] and *trans*-[OsCl(PPh_3_)(L − L)_2_]
PF_6_(L − L = *o* − C_6_ H_4_ (TeMe)_2_,
RTe(CH_2_)_3_ TeR (R=Ph or Me)). *Polyhedron*.

[643] Chivers T, Fedorchuk C, Schatte G, Brask JK (2002). Syntheses and X-ray structures of boraamidinate complexes of lithium, phosphorus, and tellurium. *Canadian Journal of Chemistry*.

[644] Zukerman-Schpector J, Haiduc I, Camillo RL, Comasseto JV, Cunha RLOR, Jorge A (2002). Supramolecular self-assembly through telluriumhalogen secondary bonds: a hexagonal grid of Te_2_Cl_2_ and Te_6_Cl_6_ rings in the solid state structure of 1,1,3-trichloro-2,4,5,6-tetrahydro-1*H*-1*λ*
^4^-benzo[b]tellurophene. *Canadian Journal of Chemistry*.

[645] Zukerman-Schpector J, Camillo RL, Comasseto JV, Cunha RLOR, Caracelli I (2000). Benzyltriethylammonium 2,2,2,4-tetrachloro-2,5-dihydro-1,2*λ*
^5^-oxatellurole. *Acta Crystallographica, Section C*.

[646] Russo U, Valle G, Calogero S (1980). Crystal and molecular structure of bis(*NN*′ -dimethylformamidine) disulphide hexachlorotellurate. *Journal of the Chemical Society, Dalton Transactions*.

[647] Chivers T, Schatte G (2002). Pyramidal inversion isomers in the solid-state structure of the tricyclic antimony-tellurium imido complex TeSb_2_Cl_2_(NtBu)_4_. *Inorganic Chemistry*.

[648] Gockel S, Haas A, Probst V, Boese R, Müller I (2000). Contributions to bis(perfluoroalkyl) chalkogenide chemistry: preparation of 
(R_f_)_2_ SeO[R_f_ = C_2_F_5_, (CF_3_)_2_CF, *n* − C_4_F_9_],(R′ _*f*_)_2_TeX_2_[X=F,CI : R′ _*f*_ = *n* − C_4_F_9_;X = Br : R′ _*f*_ = *n* − C_3_F_7_, *n* − C_4_F_9_], (CF_3_)_2_ Te (NSO)_2_, and (C_2_F_5_)_2_ Te(OH)NO_3_. *Journal of Fluorine Chemistry*.

[649] Balde L, Julien R, Morgant G (2001). Crystal structure of 5-azoniaoctane-1,8-diammonium hexachloro tellurate(IV)–hydrogen chloride (1/1), (TeCI_6_)(C_7_N_3_H_21_) ⋅ HCI. *Zeitschrift für Kristallographie: New Crystal Structures*.

[650] Laitalainen T, Sundberg MR, Uggla R, Bergman J (1997). Stereoselective synthesis, molecular structure and NBO analyses of *cis*-3,5-di(chloromethyl)-1,4-oxatellurane(IV) 4,4-dichloride. *Polyhedron*.

[651] Reich O, Hasche S, Bonmann S, Krebs B (1998). [H_3_O ⋅ dibenzo-18 – crown – 6)][Te_2_Br_9_] and [H_5_O_2_][Te_2_CI_9_] ⋅ 2C_4_H_8_O_2_: two new oxonium halotellurates (IV) containing a novel type of [Te_2_X_9_] anions. *Zeitschrift für anorganische und allgemeine Chemie*.

[652] Liaw W-F, Chiou S-J, Lee G-H, Peng S-M (1998). A discrete chlorotellurate [CI_4_Te − Mn(CO)_5_]^−^: coordinative addition of the
metalloanion [Mn(CO)_5_]^−^ to TeCl_4_. *Inorganic Chemistry*.

[653] Fleischer H, Dienes Y, Schollmeyer D (2002). Tellurium(IV) tetraalkoxides and chlorotellurium(IV) alkoxides derived from *β*-donor alcohols. *European Journal of Inorganic Chemistry*.

[654] Viossat B, Khodadad P, Rodier N (1981). Structure cristalline de l'hexachlorotellurate(IV) d'hydroxydiméthylsoufrediméthylsulfoxyde (1/2) [(CH_3_)_2_SOH]_2_(TeCI_6_) ⋅ 2(CH_3_)_2_SO. *Journal of Molecular Structure*.

[655] Pietikäinen J, Maaninen A, Laitinen RS, Oilunkaniemi R, Valkonen J (2002). Halogenation of tellurium by SO_2_CI_2_. Formation and crystal structures of (H_3_O)[Te_3_CI_13_] ⋅ 1/2SO_2_, [(C_4_H_8_O)_2_H][TeCI_5_] ⋅ (C_4_H_8_O), [(Me_2_SO)_2_H]_2_[TeCI_6_] and [Ni(NCCH_3_)_6_][Te_2_CI_10_]. *Polyhedron*.

[656] Alcock NW, Harrison WD (1982). Secondary bonding. Part 7. Crystal and molecular structures of diphenyltellurium dichloride and phenyltellurium trichloride. *Journal of the Chemical Society, Dalton Transactions*.

[657] Einstein FWB, Jones T (1982). Structure of phenyltellurium trichloride. *Acta Crystallographica, Section B*.

[658] Singh HB, Sudha N, Butcher RT (1992). Synthesis and characterization of novel chiral ortho-tellurated complexes derived from [(*S*)-1-(dimethylamino)ethyl]benzene: crystal and molecular structure of {2-[(*S*)-1-(dimethylamino)ethyl]phenyl}tellurium trichloride. *Inorganic Chemistry*.

[659] Collins PH, Webster M (1974). Crystal and molecular structure of tetraphenylarsonium aquotetrachlorohydroxotellurate(IV). *Journal of the Chemical Society, Dalton Transactions*.

[660] Krebs B, Paulat V (1979). Darstellung und Eigenschaften trimerer Chlorotellurate(IV). Kristallstruktur von (C_6_H_5_)_3_CTe_3_CI_13_. *Zeitschrift für Naturforschung, Section B*.

[661] Zukerman-Schpector J, Castellano EE, Comasseto JV, Santos RA (1993). Structure of dichloro[(2,4-dimethyl-5-oxo-2,3,4,5-tetrahydro-2-furyl)methyl]-(4-methoxyphenyl)tellurium(IV). *Journal of Chemical Crystallography*.

[662] Korp JD, Bernal I, Turley JC, Martin GE (1980). Crystal and molecular structure of phenoxatellurin 10,10-dichloride. *Inorganic Chemistry*.

[663] Waśkowska A, Janczak J, Czapla Z (1993). Crystal structure of diguanidine hexachlorate tellurate(IV). *Journal of Alloys and Compounds*.

[664] Sundberg MR, Uggla R, Laitalainen T, Bergman J (1994). Influence of secondary bonding on the intradimer distance of trichloro(ethane-1,2-diolato-*O*, *O*′)tellurate(IV). *Journal of the Chemical Society, Dalton Transactions*.

[665] Dabdoub MJ, Justino A, Guerrero PG, Zukerman-Schpector J (1998). Unexpected reaction of 1-butyltelluro-4-phenyl-1-buten-3-yne under Rupe reaction conditions. *Organometallics*.

[666] Khodadad P, Viossat B, Toffoli P, Rodier N (1979). *Acta Crystallographica, Section B*.

[667] Baldé L, Julien R, Silvestre J-P, Jouan M (2001). Crystal structure of cadaverine (1,5-pentanediamine)hexachlorotellurate (IV), [C_5_N_2_H_16_][TeCI_6_]. *Zeitschrift für Kristallographie: New Crystal Structures*.

[668] Zukerman-Schpector J, Haiduc I, Camillo RL, Comasseto JV, Cunha RLOR, Caracelli I (2001). Acetonyldichloro[(Z)-2-chloro-2-phenylvinyl]tellurium(IV), helical chains of metal complexes. *Acta Crystallographica, Section C*.

[669] Willey GR, Aris DR, Aemaeg W, Errington W (2001). Ligand oxidation of small-ring aza- and thia-macrocycles involving C—H activation: crystal structures of [MeN(CH_2_NMe)_2_CH]_2_[MX_6_] ⋅ MeCN (M=Te, X=CI, Br; M=Sn, X=Br and [C_6_H_11_S_3_]_2_TeBr_6_] ⋅ MeCN). *Inorganica Chimica Acta*.

[670] Takaguchi Y, Horn E, Furukawa N (1996). Preparation and X-ray structure analysis of 1,1,5,5,9,9-hexachloro-1,5,9-tritelluracyclododecane (Cl_6_([12]aneTe_3_)) and its redox behavior. *Organometallics*.

[671] Marsh RE (2005). Space group *p*: an update. *Acta Crystallographica, Section B*.

[672] Ryan JM, Xu Z (2004). [C_6_H_5_NH(CH_3_)_2_]_2_
Te_2_I_10_: secondary I—I bonds build up a 3D network. *Inorganic Chemistry*.

[673] Beckmann J, Dakternieks D, Duthie A, Mitchell C (2005). An orthorhombic polymorph of dichlorobis[4-(dimethylamino)phenyl]tellurium. *Acta Crystallographica, Section E*.

[674] Ishida H, Kashino S (1998). Bis(dimethylammonium) hexachlorotellurate(IV). *Acta Crystallographica, Section C*.

[675] Singh HB, Sudha N, West AA, Hamor TA (1990). Orthotellurated derivatives of *N*, *N*-dimethylbenzylamine: crystal and molecular structures of [2-(dimethylaminomethyl)phenyl]tellurium(IV) tribromide and [2-(butyldichlorotelluro)benzyl]dimethylammonium chloride. *Journal of the Chemical Society, Dalton Transactions*.

[676] Lau C, Neumüller B, Dehnicke K (1996). Synthese und kristallstruktur des tellur-nitridchlorids [Te_11_N_6_CI_26_]. *Zeitschrift für anorganische und allgemeine Chemie*.

[677] Zingaro RA, Pathirana HMKK, Reibenspies JH, Meyers EA (1991). Reactions of tellurium tetrahalides with glycols. *Phosphorus, Sulfur and Silicon and the Related Elements*.

[678] Zukerman-Schpector J, Caracelli I, Dabdoub MJ, Dabdoub VB, Pereira MA (1996). (*Z*)-1-(dichloro-*p*-methoxyphenyltelluro)-1-phenyl-2-thiophenylethene. *Acta Crystallographica, Section C*.

[679] Cameron TS, Amero RB, Cordes RE (1980). *Crystal Structure Communications*.

[680] Asahara M, Taomoto S, Tanaka M, Erabi T, Wada M (2003). Dependence of the rotational barrier of the Ar-group in RArTeX_2_ on the R-group [Ar = 2,6 −(MeO)_2_C_6_H_3_; R = Me, Et, *i*-*Pr*; X = Cl, Br, I]. *Dalton Transactions*.

[681] Fleischer H, Mathiasch B, Schollmeyer D (2002). Adducts of tellurium tetrachloride with allyl alcohol and allyl acetate: 1,2- vs 1,3-addition and structure and dynamics of Te—O interactions in different phases. *Organometallics*.

[682] Pietikainen J, Laitinen RS, Valkonen J (1999). Preparation and crystal structure of [(Me_3_Si)_2_N]_2_ TeCI_2_. *Acta Chemica Scandinavica*.

[683] Roesky HW, Münzenberg J, Bohra R, Noltemeyer M (1991). Syntheses and crystal structures of compounds containing short Te—N bonds. *Journal of Organometallic Chemistry*.

[684] Tamura R, Shimizu H, Ono N, Azuma N, Suzuki H (1992). New carbon-carbon bond formation reactions using bis(acylmethyl)- and bis[(alkoxycarbonyl)methyl]tellurium dichlorides. *Organometallics*.

[685] Ishida H, Kashino S (1992). Structure of *tert*-butylammonium hexachlorotellurate(IV). *Acta Crystallographica, Section C*.

[686] Zukerman-Schpector J, Castellano EE, Comasseto JV, Stefani HA (1988). Structure of dichloro(*p*-methoxyphenyl)(2-oxocyclohexyl)tellurium(IV). *Acta Crystallographica, Section C*.

[687] Koch H-J, Roesky HW, Besser S, Herbst-Irmer R (1993). Synthese und Struktur des ersten Tellur-haltigen Borazin-Derivats und einer Tellur-haltigen Bor—Stickstoff-Spiro-Verbindung. *Chemische Berichte*.

[688] Dakternieks D, O'Connell J, Tiekink ERT (2000). Synthesis and crystal structures of the monomeric organotellurium(IV) trihalides: *trans*-2-ethoxy-cyclohexyl-tellurium(IV) trichloride, trichloro(2-chlorobicyclo[2.2.1]hept-7-yl)-*λ*
^4^-tellurane, and mesityltellurium(IV) tribromide. *Journal of Organometallic Chemistry*.

[689] Lang ES, Fernandes RM, Peppe C, Burrow RA, Vázquez-López EM (2003). Tellurium-halogen secondary bonding in the crystal structures of [Q]^+^[PhTeCl_4_]^−^ (Q=C_5_NH_6_, 2-Br-C_5_NH_5_, {2-Br-C_5_NH_5_} {CO(NH_3_)_4_CI_2_}). *Zeitschrift für anorganische und allgemeine Chemie*.

[690] Fleischer H, Schollmeyer D (2004). Spectroscopic investigation of the system TeCl_4_
/[NEt_4_]PF_6_ in solution and the crystal structure of [NEt_4_]_2_[Te_2_Cl_10_]. *Zeitschrift für Naturforschung, Section B*.

[691] Naumann D, Ehmanns L, Tebbe K-F, Crump W (1993). Kristallstruktur-Untersuchungen von Bis(pentafluorphenyl)tellurdihalogeniden C_6_F_5_TeHal_2_ (Hal = Cl, Br). *Zeitschrift für anorganische und allgemeine Chemie*.

[692] Chivers T, Doxsee DD, Gao X, Parvez M (1994). Preparations and X-ray structures of compounds containing the four-membered ring. *Inorganic Chemistry*.

[693] Haas A, Pryka M (1995). *Chemische Berichte*.

[694] Zukerman-Schpector J, Comasseto JV, Stefani HA (1995). Dichloro[(*Z*)-2-chloro-2-phenylvinyl](4-methoxyphenyl)tellurium(IV). *Acta Crystallographica, Section C*.

[695] Folkerts H, Dehnicke K, Magull J (1995). *Zeitschrift für Naturforschung, Section B*.

[696] Farran J, Alvarez-Larena A, Piniella JF, Germain G, Torres-Castellanos L (1995). *Zeitschrift für Kristallographie*.

[697] Meyers EA, Zingaro RA, Comasseto JV, Stefani HA, Chieffi A (1995). *Zeitschrift für Kristallographie*.

[698] Borecka B, Cameron TS, Malik MA, Smith BC (1995). Stereospecific reactions of aryltellurium(IV) trichlorides with 3-cyclohexene-1-methanol and 3-cyclohexene-1,1-dimethanol: the X-ray crystal structure of 2′, 4′-dimethoxyphenyl(*trans*-6-oxabicyclo[3.2.1]oct-4-yl)tellurium(IV) dichloride. *Canadian Journal of Chemistry*.

[699] Meyers EA, Junk T, Irgolic KJ (1995). *Zeitschrift für Kristallographie*.

[700] Lau C, Neumüller B, Hiller W (1996). Se_2_NBr_3_, Se_2_NCl_5_, Se_2_NCl^−^
_6_: new nitride halides of selenium(III) and selenium(IV). *Chemistry - A European Journal*.

[701] Krebs B, Hucke M, Hein M, Schaffer A (1983). *Zeitschrift für Naturforschung, Section B*.

[702] Krebs B, Schaffer A, Hucke M (1982). *Zeitschrift für Naturforschung, Section B*.

[703] Maaninen A, Boeré RT, Chivers T, Parvez M (1999). Preparation and X-ray structure of 4-N,N′-bis(trimethylsilyl)-amino-3,5-diisopropylphenylselenium trichloride. *Zeitschrift für Naturforschung, Section B*.

[704] Wudl F, Zellers ET (1980). 1,1-Dichloro-2,5-bis(*N*-chlorothioimino)-3,4-dicyanoselenophene. *Journal of the American Chemical Society*.

[705] Roesky HW, Weber K-L, Seseke U (1985). Structural and nuclear magnetic resonance studies of short selenium-nitrogen bonds. *Journal of the Chemical Society, Dalton Transactions*.

[706] Müller U, Eckhoff B (1999). Crystal structure of bis(tetramethylammonium)-hexachloroselenate(IV) - Acetonitrile (1/1), [N(CH_3_)_4_]_2_[SeCl_6_] ⋅ CH_3_CN, a structure related to epasolite. *Zeitschrift für Kristallographie: New Crystal Structures*.

[707] Amendola A, Gould ES, Post B (1964). The crystal structure of 1,4-diselenane tetrachloride. *Inorganic Chemistry*.

[708] Krebs B, Rieskamp N, Schaffer A (1986). *Zeitschrift für anorganische und allgemeine Chemie*.

[709] Wang B-C, Cordes AW (1970). Crystal structure of dipyridinium(II) oxytetrachloroselenate(IV),C_10_H_8_N_2_H_2_
^2+^SeOCl_4_
^2−^. Highly coordinated selenium compound. *Inorganic Chemistry*.

[710] Cordes AW, Oakley RT, Reed RW (1986). Structure of 1,1-dichloro-3,5-diphenyl-4*H*-1,2,4,6-selenatriazine. *Acta Crystallographica, Section C*.

[711] Marsden CJ, Sheldrick GM, Taylor R (1977). *Acta Crystallographica, Section B*.

[712] Thompson MD, Berlin KD, Smith GS, van der Helm D, Muchmore SW, Fidelis KA (1986). *Organic Preparations and Procedures International*.

[713] Privett AJ, Craig SL, Jeter DY, Cordes AW, Oakley RT, Reed RW (1987). Structure of *N*-(*N*-chlorobenzimidoyl)benzamidinium decachlorodiselenate(IV) acetonitrile solvate. *Acta Crystallographica, Section C*.

[714] Herberhold M, Keller M, Kremnitz W (1998). Phenylselenolato complexes of cyclopentadienylrhodium: structural variety in the solid state and in solution. *Zeitschrift für anorganische und allgemeine Chemie*.

[715] Czado W, Maurer M, Müller U (1998). Chloroselenate mit zwei- und vierwertigem Selen: ^77^Se-NMR-Spektren, Synthese und Kristallstrukturen von (PPh_4_)_2_SeCl_6_ ⋅ 2CH_2_Cl_2_, (NMe_3_Ph)_2_SeCl_6_, (K-18-Krone-6)_2_SeCl_6_ ⋅ 2CH_3_CN, PPh_4_Se_2_Cl_9_. *Zeitschrift für anorganische und allgemeine Chemie*.

[716] Vogler S, Dehnicke K (1992). *Zeitschrift für Naturforschung, Section B*.

[717] Gillespie RJ, Kent JP, Sawyer JF (1990). Reactions of S_4_N_4_ and S_3_
N_3_
Cl_3_ with selenium chlorides. The preparations and crystal structures of SeS_2_
N_2_Cl_2_, (S_5_
N_5_)(SeCl_5_), and the disordered materials (Se_*x*_S_3_
_−_
_*x*_
N_2_Cl)(SbCl_6_). *Inorganic Chemistry*.

[718] Akabori S, Takanohashi Y, Aoki S, Sato S (1991). Correlation between the structure and reactivity of the selenide dihalide of the new reducing reagent NaBH_4_−R_2_SeX_2_ on the highly selective reduction of amides. X-ray molecular structure of bis-(2-chloroethyl)selenium dichloride. *Journal of the Chemical Society, Perkin Transactions 1*.

[719] Geiser U, Schlueter JA, Dudek JD, Williams JM (1996). Structure of bis(ethylenedithio)tetrathiafulvalenium dichlorocyanoselenate (
2:1), (BEDT − TTF)_2_Cl_2_SeCN. *Molecular Crystals and Liquid Crystals Science and Technology, Section A*.

[720] McCullough JD, Hamburger G (1942). The crystal structure of diphenylselenium dichloride. *Journal of the American Chemical Society*.

[721] Lindqvist I, Nahringbauer G (1959). The crystal structure of SeOCl_2_ ⋅ 2C_5_H_5_N. *Acta Crystallographica*.

[722] Stammler HG, Weiss J

[723] Hauge S, Janickis V, Marøy K (1998). Crystal structures of phenyltrimethylammonium salts of hexabromodiselenate(II), [C_6_H_5_(CH_3_)3N]_2_[Se_2_Br_6_], hexachlorodiselenate(II), [C_6_H_5_(CH_3_)3N]_2_[Se_2_Cl_6_], and a mixed bromo-chlorodiselenate (II), [C_6_H_5_(CH_3_)3N]_2_[Se_2_Br_5_
Cl]. *Acta Chemica Scandinavica*.

[724] McCullough JD, Marsh RE (1950). The crystal structure of di-*p*-tolyselenium dichloride and di-*p*-tolylselenium dibromide. *Acta Crystallographica*.

[725] Neumüller B, Lau C, Dehnicke K (1996). Die Kristallstrukturen von SeCl_3_
^+^SbCl_6_
^−^, SeBr_3_
^+^
GaBr_4_
^−^
, PCl_4_
^+^
SeCl_5_
^−^
, und PPh_4_SeCl_4_
^2−^⋅2CH_3_
CN. *Zeitschrift für anorganische und allgemeine Chemie*.

[726] Fenske D, Ergezinger C, Dehnicke K (1989). *Zeitschrift für Naturforschung, Section B*.

[727] Ahlers F-P, Lührs E, Krebs B (1991). Synthese, Struktur und Eigenschaften der neuen trinuklearen Halogenoselenate(IV) [Se_3_Cl_13_
]^−^ und [Se_3_Br_13_]^−^ Kristallstrukturen von [Ph_3_C][Se_3_Cl_13_] und [Ph_3_C][Se_3_Br_13_]. *Zeitschrift für anorganische und allgemeine Chemie*.

[728] Heckmann G, Wolmershauser G (1993). *Chemische Berichte*.

[729] Folkerts H, Dehnicke K, Magull J, Goesmann H, Fenske D (1994). Phosphaniminato-trichloroselenate(II): synthese und kristallstrukturen von [SeCl(NPPh_3_)_2_]^+^SeCl_3_
^−^ und [Me_3_SiN(H)PMe_3_]_2_
^+^[Se_2_Cl_6_]^2−^. *Zeitschrift für anorganische und allgemeine Chemie*.

[730] Martin LD, Perozzi EF, Martin JC (1979). Sulfuranes. 39. Syntheses and structure studies of stable difluoro- and dichlorosulfuranes. Apicophilicity orders in sulfuranes. *Journal of the American Chemical Society*.

[731] Baenziger NC, Buckles RE, Maner RJ, Simpson TD (1969). Crystal structure of the chlorine complex of bis(*p*-chlorophenyl) sulfide. *Journal of the American Chemical Society*.

[732] Kuhn N, Bohnen H, Fahl J, Bläser D, Boese R (1996). On the reaction of l,3-diisopropyl-4,5-dimethylimidazol-2-ylidene with sulfur halides and sulfur oxygen halides. *Chemische Berichte*.

[733] Lang ES, Burrow RA, Braga AL, Dornelles L (2000). Crystal structure of (2E)-3-bromo-2-[bromo(chloro)phenyl-*λ*
^4^-tellanyl]-2-propen-1-ol, BrCH=
C(TeBrClPh)(CH_2_OH). *Zeitschrift für Kristallographie: New Crystal Structures*.

[734] Dahan F, Lefebvre-Soubeyran O (1976). *Acta Crystallographica, Section B*.

[735] Chauhan AKS, Kumar A, Srivastava RC, Beckmann J, Duthie A, Butcher RJ (2004). Synthesis and reactivity of para-substituted benzoylmethyltellurium(II and IV) compounds: observation of intermolecular C-H-O hydrogen bonding in the crystal structure of (p − MeOC_6_H_4_ COCH_2_)_2_TeBr_2_. *Journal of Organometallic Chemistry*.

[736] Kuhn N, Abu-Rayyan A, Eichele K, Schwarz S, Steimann M (2004). Weak interionic interactions in 2-bromoimidazolium derivatives. *Inorganica Chimica Acta*.

[737] Hazell AC (1972). *Acta Chemica Scandinavica*.

[738] Alcock NW, Harrison WD (1982). *Acta Crystallographica, Section B*.

[739] Knobler C, McCullough JD (1977). Crystal and molecular structure of 2-biphenylyltellurium tribromide, C_12_
H_9_TeBr_3_. *Inorganic Chemistry*.

[740] Schnabel W, von Deuten K, Klar G (1982). *Phosphorus and Sulfur*.

[741] Chitsaz S, Neumüller B, Dehnicke K (1999). Crystal structure of bis(bromotriphenyl)-arsenic(V)-hexabromotellurate(IV), [Ph_3_
AsBr]_2_[TeBr_6_]. *Zeitschrift für Naturforschung, Section B*.

[742] Detty MR, Luss HR (1983). 12-Te-5 pertelluranes from 1,6-dioxa-6a-tellurapentalenes. Synthesis, structure, and reactivity. *Journal of Organic Chemistry*.

[743] Behmel P, Jones PG, Sheldrick GM, Ziegler M (1980). Untersuchungen zur molekülstruktur von Carbon-säureamid-Verbindungen der Säure H_2_TeBr_6_. *Journal of Molecular Structure*.

[744] Srivastava PC, Sinha A, Bajpai S, Schmidt HG, Noltemeyer M (1999). Dimethyl tellurium (IV) derivatives: synthesis, spectroscopic characterisation and structures of Me_2_TeBr_2_ and Me_2_
Te(OCOC_6_H_5_)_2_. *Journal of Organometallic Chemistry*.

[745] Borgias BA, Scarrow RC, Seidler MD, Weiner WP (1985). *Acta Crystallographica, Section C*.

[746] Hammerschmidt A, Bonmann S, Läge M, Krebs B (2004). Novel halogenochalcogeno(IV) acids: [H_3_
O(benzo-18
-crown-6)]_2_[Te_2_Br_10_] and [H_5_
O_2_(dibenzo-24
-crown-8
)]_2_[Te_2_Br_10_]. *Zeitschrift für anorganische und allgemeine Chemie*.

[747] Detty MR, Luss HR, McKelvey JM, Geer SM (1986). 12-Te-5 pertelluranes from 1,2-oxatellurolyl-1-ium halides. Synthesis, structure, and reactivity. The quest for delocalization in 10-Te-3 telluranes and 12-Te-5 pertelluranes of thiathiophthene structure. *Journal of Organic Chemistry*.

[748] Christofferson GD, McCullough JD (1958). The crystal structure of diphenyltellurium dibromide. *Acta Crystallographica*.

[749] Beckmann J, Dakternieks D, Duthie A, Mitchell C (2004). Dibromodiphenyltellurium(IV). *Acta Crystallographica, Section E*.

[750] Cameron TS, Amero RB, Chan C, Cordes RE (1980). *Crystal Structure Communications*.

[751] Al-Salim N, West AA, McWhinnie WR, Hamor TA (1988). 2-Pyridyl- and quinolin-2-yl-functionalised organyltellurium ligands. The stabilisation of diorganyl tritellurides. The crystal and molecular structures of 2-(2-pyridyl)phenyltellurium(IV) tribromide, dimethyldithiocarbamato[2-(2-pyridyl)phenyl]tellurium(II), and *p*-ethoxyphenyl 2-(2-pyridyl)phenyl telluride. *Journal of the Chemical Society, Dalton Transactions*.

[752] Janickis V, Herberhold M, Milius W (2003). Synthesis and crystal structure of bis(methyltriphenylphosphonium) hexabromotellurate(IV)-bis{dibromoselenate(II)}, [PMePH_3_]_2_[TeBr_6_(SeBr_2_)_2_], the salt of a mixed-valence bromotellurate(IV)-selenate(II) anion. *Zeitschrift für anorganische und allgemeine Chemie*.

[753] Lang ES, de Oliveira GM, Fernandes RM, Vázquez-López EM (2003). Synthesis and characterization of the first [Q]^+^
[PhTeX_4_]^−^ complex salt (Q = 2-Br-C_5_
NH_5_; Ph = C_6_
H_5_; X=Br, I) exhibiting a polymeric chain structure. *Inorganic Chemistry Communications*.

[754] Abriel W, du Bois A (1989). Structure of bis(tetraphenylarsonium) hexabromotellurate(IV). *Acta Crystallographica, Section C*.

[755] Farran J, Alvarez-Larena A, Piniella JF, Germain G, Torres-Castellanos L (1995). Dibromobis(4-methoxyphenyl)tellurium(IV). *Acta Crystallographica, Section C*.

[756] Janickis V, Nečas M, Novosad J, Dušek M, Petříček V (2002). Commensurate and incommensurate structures of the hexabromotellurate(IV) bis{dibromodiselenate(I)} ion - [(C_2_
H_5_)_*n*_(C_6_H_5_)_4_
_−_
*_n_*P]_2_[TeBr_6_
(Se_2_Br_2_)_2_], 
*n*=
0,1. *Acta Crystallographica, Section B*.

[757] Kunnari SM, Oilunkaniemi R, Laitinen RS, Ahlgrén M (2001). An unexpected tetrahydrofuran ring opening: synthesis and structural characterization of Ph_3_
PO(CH_2_)_4_TeBr_4_. *Journal of the Chemical Society, Dalton Transactions*.

[758] Mallikaratchy P, Norman RE, Fronczek FR, Junk T (2003). Tribromo(3,5-dimethyl-2-nitro-phenyl-*κ*
^2^
*C*
^1^,*O*)tellurium(IV), bromo(3,5-dimethyl-2-nitro-phenyl-*κ*
^2^
*C*
^1^,*O*)tellurium(II) and bromo(3,5-dimethyl-2-nitroso-phenyl-*κ*
^2^
*C*
^1^,*O*)tellurium(II). *Acta Crystallographica, Section C*.

[759] Krebs B, Büscher K (1980). Dimere Halogenotellurate(IV): Darstellung und Kristallstruktur von [(C_6_
H_5_)_4_P]_2_Te_2_Br_10_. *Zeitschrift für anorganische und allgemeine Chemie*.

[760] Janickis V, Herberhold M, Necas M, Milius W (2003). Synthesis and crystal structure of Bis(methyltriethylammonium) Hexabromotellurate(IV)-tris{dibromodiselenate(i)}, [NMeEt_3_]_2_
_*n*_[TeBr_6_(Se_2_Br_2_)_3_]_*n*_, containing a chain-polymeric mixed-valence bromotellurate(IV)-selenate(I) anion. *Zeitschrift für anorganische und allgemeine Chemie*.

[761] Eveland JR, Whitmire KH (1997). Synthesis and characterization of the carbide cubane cluster [Fe_3_(Co)_9_Te_4_(*μ*
_3_−CTeBr_4_
)] with an unusual tetrahedral CTe_4_ unit. *Angewandte Chemie International Edition*.

[762] Menon SC, Singh HB, Patel RP, Das K, Butcher RJ (1997). Synthesis and reactivity of chiral tellurium azomethines: pseudopolymorphism of [o-((((1S,2R)-2-hydroxy-2-phenyl-1-methylethyl)amino)-methinyl)phenyl] tellurium(IV) bromide. *Organometallics*.

[763] Zukerman-Schpector J, Stefani HA, Silva DDO (1998). Dibromo[(*Z*)-2-bromo-2-(hydroxymethyl)-vinyl](*n*-butyl)tellurium(IV). *Acta Crystallographica, Section C*.

[764] Chadha RK, Nguyen T (1990). Structure of dibromo(2-methoxycyclohexyl)phenyltellurium. *Acta Crystallographica, Section C*.

[765] Knobler C, McCullough JD (1972). Crystal and molecular structure of 1-thia-4-telluracyclohexane 4,4-dibromide, C_4_
H_8_
STeBr_2_. *Inorganic Chemistry*.

[766] Dahan F, Lefebvre-Soubeyran O (1976). *Acta Crystallographica, Section B*.

[767] Klapötke TM, Krumm B, Mayer P, Piotrowski H, Ruscitti OP (2002). Chlorination and bromination of dialkyl tellurides. *Zeitschrift für anorganische und allgemeine Chemie*.

[768] Reich O, Hasche S, Büscher K, Beckmann I, Krebs B (1996). Neue Oxonium-bromochalkogenate(IV) - Darstellung, Struktur und Eigenschaften von [H_3_O][TeBr_5_] ⋅ 3C_4_H_8_O_2_ und [H_3_O]_2_[SeBr_6_]. *Zeitschrift für anorganische und allgemeine Chemie*.

[769] Berg RW, Nielsen K (1979). *Acta Chemica Scandinavica, Series B*.

[770] Chauhan AKS, Kumar A, Srivastava RC, Butcher RJ (2002). Synthesis and characterization of monomeric diorganotellurium dihalides: crystal and molecular structures of diphenacyltellurium dibromide and - diiodide. *Journal of Organometallic Chemistry*.

[771] Hauge S, Janickis V, Marøy K (1999). Reaction of TeBr_4_ with SbBr_3_ in the presence of [C_6_
H_5_(CH_3_)_3_
N]Br: crystal structures of [C_6_
H_5_(CH_3_)_3_N]_2_[Te_2_Br_10_] and [C_6_H_5_
(CH_3_)_3_
N][SbTeOBr_6_]. *Acta Chemica Scandinavica*.

[772] Bakshi PK, Cameron TS, Ali MES, Malik MA, Smith BC (1993). Reactions of *trans*-2-alkoxy-1-cycloalkyltellurium(IV) trihalides with N-substituted anilines: the X-ray crystal structure of *p*-*N*-ethylanilino(*trans*-2-ethoxy-1-cyclohexyl)tellurium(IV) dibromide. *Inorganica Chimica Acta*.

[773] Dakternieks D, O'Connell J, Tiekink ERT (2000). Crystal structure of dibromo[(1,1-dibromo-1-mesityl-*λ*
^4^-telluranyl)methyl]-mesityl-*λ*
^4^-tellurane, [MesTeBr_2_]_2_CH_2_. *Zeitschrift für Kristallographie: New Crystal Structures*.

[774] Lu W-M, Wang Y-P, Huang XJ, Sun JIA (2001). Molecular structures of dibromo[(E)-2-bromo-2-phenylvinyl]-(phenyl)tellurium(IV) and dibromo [(Z)-2-bromo-2-phenyl-vinyl] (p-tulyl)tellurium (IV) hydrate methanolate. *Chinese Journal of Chemistry*.

[775] Janickis V, Songstad J, Tornroos KW (2001). Syntheses and crystal structure of bis (phenyltrimethyl ammonium) hexabromotellurate (IV)bis{dibromodiselenate(I)}, [C_6_H_5_(CH_3_)_3_N]_2_[TeBr_6_(Se_2_Br_2_)_2_]. *Chemija*.

[776] Klapötke TM, Krumm B, Mayer P, Naumann D, Schwab I (2004). Fluorinated tellurium(IV) azides and their precursors. *Journal of Fluorine Chemistry*.

[777] Baker L-J, Rickard CEF, Taylor MJ (1995). Crystal structure determination and vibrational spectra of (*t*−BuNH_3_)_2_[TeBr_6_] and comparisons with other solids containing [TeCl_6_]^2−^
or [TeBr_6_]^2−^
ions. *Polyhedron*.

[778] Devillanova FA, Deplano P, Isaia F (1998). Crystal structure and vibrational characterization of the reaction products of *N*-methylthiazolidine-2(3H)-selone (1) and *N*-methylbenzothiazole-2(3H)-selone (2) with Br_2_. *Polyhedron*.

[779] Krebs B, Schaffer A, Pohl S (1984). *Zeitschrift für Naturforschung, Section B*.

[780] Takada H, Metzner P, Philouze C (2001). First chiral selenium ylides used for asymmetric conversion of aldehydes into epoxides. *Chemical Communications*.

[781] Berges P, Hinrichs W, Klar G (1986). *Journal of Chemical Research*.

[782] Abriel W (1987). *Zeitschrift für Naturforschung, Section B*.

[783] Godfrey SM, Jackson SL, McAuliffe CA, Pritchard RG (1998). Reaction of tertiary phosphine selenides, R_3_PSe (R = Me_2_N, Et_2_N or C_6_H_11_), with dibromine. The first reported examples of 1 : 1 addition. *Journal of the Chemical Society, Dalton Transactions*.

[784] Hammerschmidt A, Beckmann I, Läge M, Krebs B (2005). A novel halogenochalcogeno(IV)acid: [H_3_O(Dibromo-benzo-15
-Krone-5)]_2_[SeBr_6_]. *Zeitschrift für anorganische und allgemeine Chemie*.

[785] Hauge S, Maroy K, Odegard T (1988). *Acta Chemica Scandinavica, Series A*.

[786] Krebs B, Luhrs E, Ahlers F-P (1989). Bromoselenates(II,IV), a novel type of mixed valence compounds. *Angewandte Chemie International Edition*.

[787] Hauge S, Janickis V, Marøy K (1998). Syntheses and crystal structures of salts of hexabromotetraselenate(I) and hexabromoselenate(IV)bis{dibromodiselenate(I)}. *Acta Chemica Scandinavica*.

[788] Aragoni MC, Arca M, Demartin F (2001). Mechanistic aspects of the reaction between Br_2_ and chalcogenone donors (LE; E = S, Se): competitive formation of 10-E-3, T-shaped 1:1 molecular adducts, charge-transfer adducts, and [(LE)_2_]^2+^
dications. *Chemistry - European Journal*.

[789] Takanohashi Y, Tabata N, Tanase T, Akabori S (1993). Bis(2-bromoethyl) selenium dibromide as the selenium-introducing reagent: one-pot preparation of 2,5-bis(alkoxymethyl)tetrahydroselenophenes by the cyclization of 1,5-hexadiene. *Journal of Organometallic Chemistry*.

[790] McCullough JD, Hamburger G (1941). The crystal structure of diphenylselenium dibromide. *Journal of the American Chemical Society*.

[791] Williams DJ, Vanderveer D, Crouse BR (1997). *Main Group Chemistry*.

[792] Jung A, Wolmershäuser G (1997). Bromination of “poly(1,4-diselenobenzene)”. *Zeitschrift für Naturforschung, Section B*.

[793] Hauge S, Janickis V, Marøy K (1998). Crystal structures of phenyltrimethylammonium salts of tetrabromoselenate(II) bromide, [C_6_H_5_(CH_3_)_3_N]_2_[SeBr_4_] ⋅ [C_6_H_5_(CH_3_)_3_N] Br and a mixed tetra(bromo/chloro)selenate(II). *Acta Chemica Scandinavica*.

[794] Battelle L, Knobler C, McCullough JD (1967). Crystal and molecular structure of 1-thia-4-selenacyclohexane-4,4-dibromide, C_4_H_8_SSeBr_2_. *Inorganic Chemistry*.

[795] Tanohashi Y, Tabata N, Tanase T, Akabori S (1993). Selenium transfer reagent: one-step alkoxyselenation of cyclohexene with bis(2-bromoethyl)selenium dibromide. *Journal of the Chemical Society, Perkin Transactions 1*.

[796] Nakanishi W, Hayashi S (2000). Inter-element linkage in 1,2- and 1,4-bis(arylselanyl)benzenes with halogens. *Journal of Organometallic Chemistry*.

[797] Geiser U, Hau Wang H, Schlueter JA (1994). Synthesis, structure, and properties of the organic conductor (BEDT-TTF)_2_Br_2_SeCN. *Inorganic Chemistry*.

[798] Miura M, Takanohashi Y, Habata Y, Akabori S (1994). Reactivities of bis(2-bromoethyl)selenium dibromide and its related compounds: formation of hypervalent T-shaped coordinated selenium compounds by reaction with pyridine and its derivatives. *Tetrahedron Letters*.

[799] Miura M, Takanohashi Y, Habata Y, Akabori S (1995). New synthesis of hypervalent T-shaped coordination compounds of selenium by the reaction of bis(2-bromoethyl)selenium dibromide with pyridine and its derivatives. *Journal of the Chemical Society, Perkin Transactions 1*.

[800] Arduengo AJ, Burgess EM (1977). Tricoordinate hypervalent sulfur compounds. *Journal of the American Chemical Society*.

[801] Hauge S, Vikane O (1983). *Acta Chemica Scandinavica, Series A*.

[802] Aragoni MC, Arca M, Blake AJ (2001). 1,2-Bis(3-methyl-imidazolin-2-ylium iodobromoselenanide)ethane: oxidative addition of IBr at the Se atom of a > C = Se group. *Angewandte Chemie International Edition*.

[803] Ledesma GN, Lang ES, Abram U (2004). 2,4,6-Triphenylphenyltellurium(IV) triiodide - supramolecular self-assembling in organotellurium triiodides. *Journal of Organometallic Chemistry*.

[804] McCullough JD (1975). Crystal and molecular structure of dibenzotellurophene diiodide C_12_H_8_Tel_2_. *Inorganic Chemistry*.

[805] Singh HB, McWhinnie WR, Hamor TA, Jones RH (1984). Synthesis and chemistry of 1,3-dihydrotellurolo[3,4-*b*]quinoxaline and derivatives: crystal and molecular structure of 1,3-dihydro-2,2-di-iodo-2*λ*
^4^-tellurolo[3,4-*b*]quinoxaline-2,3-bis(iodomethyl)quinoxaline (1 : 1). *Journal of the Chemical Society, Dalton Transactions*.

[806] Alcock NW, Harrison WD (1984). Secondary bonding. Part 12. Aryltellurium iodides: crystal and molecular structures of *cis*- and *trans*-phenyltellurium(IV) tri-iodide and two modifications of diphenyltellurium(IV) di-iodide. *Journal of the Chemical Society, Dalton Transactions*.

[807] Chao GY, McCullough JD (1962). The crystal structure of di-*p*-chlorodiphenyltellurium diiodide. *Acta Crystallographica*.

[808] McCullough JD, Knobler C, Ziolo RF (1985). Crystal and molecular structure of the *β* modification of 1,1-diiodo-3,4-benzo-1-telluracyclopentane, *β* − C_8_H_8_Tel_2_. Comparative study of secondary bonding systems and colors in organotellurium iodides. *Inorganic Chemistry*.

[809] Knobler C, Ziolo RF (1979). Organotellurium diiodides. The molecular structure of the *α* modification of I,I-Diiodo-3,4-benzo-I-telluracyclopentane, *α*
^−^C_8_H_8_Tel_2_. *Journal of Organometallic Chemistry*.

[810] Chan LYY, Einstein FWB (1972). *Journal of the Chemical Society, Dalton Transactions*.

[811] L'Haridon P, Jedrzejczak H, Szwabski S (1979). *Acta Crystallographica, Section B*.

[812] Pritzkow H (1979). Crystal and molecular structure of dimethyltellurium tetraiodide, (CH_3_)_2_Tel_4_. *Inorganic Chemistry*.

[813] Srivastava PC, Bajpai S, Bajpai S (2004). Telluranes: potential synthons for charge-transfer complexes (involving hypervalent Te-I bonds) and serendipitous synthesis of the first triphenyl methyl phosphonium salts containing [C_4_H_8_Tel_4_]^2−^ and[Tel_6_]^2−^ anions. *Journal of Organometallic Chemistry*.

[814] Hesford MJ, Hill NJ, Levason W, Reid G (2004). Synthesis and properties of the ditelluroethers *m*- and *p*-C_6_H_4_(CH2TeMe)_2_ and their Te(IV) derivatives: crystal structures of PhTeI_2_(CH_2_)_3_Tel_2_Ph, *m*-C_6_H_4_
(CH_2_Tel_2_Me)_2_ and *p*-C_6_H_4_(CH_2_Tel_2_Me)_2_. *Journal of Organometallic Chemistry*.

[815] Jones PG, Jeske J

[816] Kumar RK, Aravamudan G, Sivakumar K, Fun H-K (1999). Tetraethylammonium (*N*,*N*-diethyldithio-carbamato-*S*,*S*
^′^)tetraiodotellurate(IV). *Acta Crystallographica, Section C*.

[817] Hu N-H, Jin Z-S, Li Z-S (1991). Structure of hexamethylenetetratellurafulvalene diiodide. *Acta Crystallographica, Section C*.

[818] Einstein F, Trotter J, Williston CS (1967). The crystal structure of *β*-dimethyltellurium di-iodide. *Journal of the Chemical Society - A*.

[819] Farran J, Alvarez-Larena A, Capparelli MV, Piniella JF, Germain G, Torres-Castellanos L (1998). Two polymorphs of bis(4-methoxyphenyl)-tellurium(IV) diiodide. *Acta Crystallographica, Section C*.

[820] Hesford MJ, Levason W, Matthews ML, Orchard SD, Reid G (2003). *Dalton Transactions*.

[821] Hope H, Knobler C, McCullough JD (1973). Crystal and molecular structure of 1-oxa-4-telluracyclohexane 4,4-diiodide, C_4_H_8_OTeI_2_. *Inorganic Chemistry*.

[822] McCullough JD (1973). Crystal and molecular structure of phenoxatellurin 10,10-diiodide, C_12_H_8_OTeI_2_. *Inorganic Chemistry*.

[823] Srivastava PC, Bajpai S, Lath R, Butcher RJ (2000). Secondary bonds induced supramolecular assemblies in the crystals of 1,1,2,3,4,5-hexahydro-1,1-diiodotellurophene; 1,1,2,3,4,5,6-heptahydro-1,1-diiodotellurane and 1,3-dihydro-2*λ*
^4^-benzotellurole-2,2-diyl diiodide. *Journal of Organometallic Chemistry*.

[824] Knobler C, McCullough JD, Hope H (1970). Crystal and molecular structure of 1-thia-4-telluracyclohexane 4,4-diiodide, C_4_H_8_STeI_2_. *Inorganic Chemistry*.

[825] Al-Rubaie AZ, Uemura S, Masuda H (1991). New cyclic tellurides. Synthesis, reaction and ligand properties of 2,2,6,6-tetramethyl-1-oxa-4-tellura-2,6-disilacyclohexane (C_6_H_16_OSi_2_Te). X-Ray structure determination of C_6_H_16_OSi_2_Tel_2_. *Journal of Organometallic Chemistry*.

[826] du Mont W-W, Meyer H-U, Kubiniok S, Pohl S, Saak W (1992). *Chemische Berichte*.

[827] Srivastava PC, Schmidt H-G, Roesky HW (1995). *Zeitschrift für Naturforschung, Section B*.

[828] Kuhn N, Kratz T, Henkel G (1996). (1,3-Diethyl-1,3-dihydro-4,5-dimethyl-2H-imidazol-2-yliden)-diiodtellur(II) [1]. *Zeitschrift für Naturforschung, Section B*.

[829] Kumar RK, Aravamudan G, Udupa MR, Seshasayee M, Selvam P, Yvon K (1996). A novel mixed ligand Te(IV) complex comprising three halides and a dithiocarbamate; synthesis and crystal structure of triiododiethyldithiocarbamatotellurium(IV), Te{(C_2_H_5_)_2_NCS_2_}I_3_. *Polyhedron*.

[830] Kuhn N, Kratz T, Henkel G (1994). *Chemische Berichte*.

[831] Maartmann-Moe K, Songstad J (1982). *Acta Chemica Scandinavica, Series A*.

[832] Aragoni MC, Arca M, Demartin F (2004). First ICN adduct with a selenium donor (R = Se): is it an ionic [RSeCN]^+^I^−^ or a “T-shaped” R(I)SeCN hypervalent compound?. *European Journal of Inorganic Chemistry*.

